# Self-reported medication adherence instruments and their applicability in low-middle income countries: a scoping review

**DOI:** 10.3389/fpubh.2023.1104510

**Published:** 2023-07-13

**Authors:** Qisty A. Khoiry, Sofa D. Alfian, Job F. M. van Boven, Rizky Abdulah

**Affiliations:** ^1^Department of Pharmacology and Clinical Pharmacy, Faculty of Pharmacy, Universitas Padjadjaran, Jatinangor, Indonesia; ^2^Center of Excellence for Pharmaceutical Care Innovation, Faculty of Pharmacy, Universitas Padjadjaran, Jatinangor, Indonesia; ^3^Department of Clinical Pharmacy and Pharmacology, University of Groningen, University Medical Centre Groningen, Groningen, Netherlands; ^4^Medication Adherence Expertise Centre of The Northern Netherlands (MAECON), Groningen, Netherlands

**Keywords:** self-reported instruments, patient-reported medication, medication adherence, chronic diseases, low-middle income countries

## Abstract

**Introduction:**

Medication non-adherence is an important public health issue, associated with poor clinical and economic outcomes. Globally, self-reported instruments are the most widely used method to assess medication adherence. However, the majority of these were developed in high-income countries (HICs) with a well-established health care system. Their applicability in low- and middle-income countries (LMICs) remains unclear. The objective of this study is to systematically review the applicability of content and use of self-reported adherence instruments in LMICs.

**Method:**

A scoping review informed by a literature search in Pubmed, EBSCO, and Cochrane databases was conducted to identify studies assessing medication adherence using self-reported instruments for patients with five common chronic diseases [hypertension, diabetes, dyslipidemia, asthma, or Chronic Obstructive Pulmonary Disease (COPD)] in LMICs up to January 2022 with no constraints on publication year. Two reviewers performed the study selection process, data extraction and outcomes assessment independently. Outcomes focused on LMIC applicability of the self-reported adherence instruments assessed by (i) containing LMIC relevant adherence content; (ii) methodological quality and (iii) fees for use.

**Findings:**

We identified 181 studies that used self-reported instruments for assessing medication adherence in LMICs. A total of 32 distinct types of self-reported instruments to assess medication adherence were identified. Of these, 14 self-reported instruments were developed in LMICs, while the remaining ones were adapted from self-reported instruments originally developed in HICs. All self-reported adherence instruments in studies included presented diverse potential challenges regarding their applicability in LMICs, included an underrepresentation of LMIC relevant non-adherence reasons, such as financial issues, use of traditional medicines, religious beliefs, lack of communication with healthcare provider, running out of medicine, and access to care. Almost half of included studies showed that the existing self-reported adherence instruments lack sufficient evidence regarding cross cultural validation and internal consistency. In 70% of the studies, fees applied for using the self-reported instruments in LMICs.

**Conclusion:**

There seems insufficient emphasis on applicability and methodological rigor of self-reported medication adherence instruments used in LMICs. This presents an opportunity for developing a self-reported adherence instrument that is suitable to health systems and resources in LMICs.

**Systematic review registration:**

https://www.crd.york.ac.uk/prospero/, identifier: CRD42022302215.

## 1. Introduction

Medication adherence is a dynamic process that evolves over time and with prolonged treatment (1). As such, patients with chronic diseases are more likely to have poor medication adherence. Poor adherence to chronic medication is associated with worsened disease control, increased cost, increased hospitalization rates, decreased quality of life, and increased mortality (2–5). Research showed that if 25% of non-adherent people become adherent, this could save $13.7 billion annually and avert 7 million hospitalizations, in the USA alone (6). Moreover, the World Health Organization highlighted adherence as a key indicator for the quality of care (7). Therefore, addressing poor medication adherence is one of the most important factors that contribute to achieving therapy goals in chronic disease management (8). Notably, adherence to chronic medication in low-and-middle-income countries (LMICs) is poorer than in high-income countries (HICs) (9, 10).

Various methods have been used to assess medication adherence, including pill count (11, 12), prescription records and claim-reviewing (13, 14), electronic monitoring devices (15–17), and self-reported instruments (18, 19). Self-reported instruments are cheap, easy to use, and practical because they can highlight the underlying concerns leading to medication non-adherence (20, 21). Particularly, self-reported instruments have become the preferred choice to assess medication adherence in LMICs due to limited resources and logistics (21, 22). However, most of the self-reported medication adherence instruments have been developed in HICs with a well-established health care system. Therefore, these instruments may have several drawbacks when used in LMICs, such as lack of local applicability and the extra costs of obtaining a license (23, 24).

Current self-reported adherence instruments assess different reasons for non-adherence such as patients' behaviors, perceptions, and beliefs that are considered as non-intentional (e.g., forgetfulness) (25, 26) and intentional (e.g., a conscious decision after balancing the pros and cons of a medication) (26, 27), and patients' experiences (e.g., condition-related factors, socioeconomic-related factors, interaction with healthcare professionals, and therapy-related factors) (25, 26, 28). Adherence is a complicated phenomenon affected by the interaction of multiple-factors (7), however there are currently no instruments that capture all potential reasons for non-adherence (28). Importantly, due to the complexities of adherence behavior, accurate self-report instruments should capture particular characteristics and non-adherence barriers (29). Evidence from a previous scoping review showed that low medication adherence across multiple LMICs was driven by the same factors and having similar reasons such as ignorance, unfavorable attitudes, and unfavorable beliefs (30). Notably, information on self-reported adherence instruments' local applicability in LMIC is needed.

Systematic reviews about self-reported instruments on medication adherence have been conducted by a number of previous studies, focusing on their general performance (21, 28, 31, 32). However, to date, there is no comprehensive review that assessed the applicability of self-reported instruments for medication adherence in LMICs. The objective of this study is to systematically review the applicability of content and use of self-reported instruments in LMICs.

## 2. Method

### 2.1. Study design

This scoping review was reported in accordance with the Preferred Reporting Items for Systematic reviews and Meta-Analyses extension for Scoping Reviews (PRISMA-ScR) guidelines (33) (Supplementary material 1). The protocol was registered at PROSPERO with number registration CRD42022302215.

### 2.2. Information sources and search strategy

Three electronic databases (PubMed, EBSCO, and Cochrane) were searched up to January 2022 with no constraints on publication year to identify studies assessing medication adherence for five common chronic diseases: hypertension, asthma, COPD, diabetes mellitus, and/or hyperlipidemia. Hand-searching was considered necessary to identify relevant articles that had been unindexed and to ensure that relevant studies were not ignored by a snowballing process which involved checking references of included studies for additional relevant studies. This review adopted the definition of adherence as the process by which patients take their medications as prescribed, composing of initiation (moment when the first dose was taken), implementation (the actual dose of the patient from the start to the last dose), and discontinuation (end of treatment) (34). The PCC mnemonic: participants (chronic disease patients), concepts (the applicability of self-reported instruments for medication adherence), and context (low middle income countries) was used to develop search terms (35). The full search strategy using a combination of medical subject heading terms and text words can be found in the Supplementary material 2.

### 2.3. Eligibility criteria

Articles, regardless paid (subscription) or free (open access), were eligible if they met the following inclusion criteria: (1) experimental and observational studies focusing on medication adherence as a primary outcome using a self-reported instrument; (2) performed among patients with hypertension, diabetes mellitus, hyperlipidemia, asthma, and/or COPD; (3) published in English, and (4) conducted in LMICs. Of note, we defined LMICs using the World Bank's, 2021 Gross National Income (GNI) per capita by range USD 1,046–4,095 (36).

Articles were excluded if: (1) no peer-reviewed article; (2) reviews, case reports, conference proceedings, opinion pieces, letters to the editor, and commentaries.

### 2.4. Selection process

One author (QAK) conducted the potential eligibility evaluation based on screening the titles and abstracts. The full texts of potentially eligible articles were retrieved and assessed by QAK. An independent second person (SDA) conducted further independent verification of the abstract and full-text screening. Any disagreements among the reviewers (QAK and SDA) were resolved using consensus.

### 2.5. Data extraction process

Relevant data from the selected articles were extracted by QAK and verified by SDA. For data extraction, a standardized form with predefined and piloted data extraction criteria was used which was manually extracted in Microsoft Excel 2010 and backed up on Google Drive.

### 2.6. Data items

In short, the following data items were extracted:

1. Study characteristics: first author, year of publication, country of the study, the aim of the study, study design, type of medication, study period, response rate of self-reported instrument, population (type of chronic disease), sample size, and adherence phases (initiation, implementation, discontinuation).
2. Characteristics of the self-reported medication adherence instruments used: instrument name, number of items, type of scoring, original language, country of development, and psychometric properties (validity or reliability value), and whether a fee for using applies.
3. The applicability of the self-reported medication adherence instruments in LMICs as further defined below.

### 2.7. Synthesis methods

We first synthesized general information of the LMIC studies that used a self-reported adherence instrument, including type of instrument used, disease area, country and study design.

Secondly, we summarized the use, content (e.g., inclusion of adherence phases) and quality of the self-reported instruments' use (e.g., response rate) in LMIC studies.

Third, the applicability of the self-reported instruments was defined by: (1) incorporating different factors for non-adherence grouped according to the WHO categories: patient related factors, medication related factors, healthcare provider and healthcare system related factors, and societal related factors) (7) taking LMIC relevant medication adherence issues into account, (2) was it formally translated and back-translated in a professional manner, (3) whether the instrument used was validated in their own country considering cultural adaptation, and (4) whether a fee applies for using self-reported instruments in LMICs. One reviewer (QAK) read all the included studies, annotated them, and identified and categorized the applicability, which were further verified by SDA.

### 2.8. Methodological quality properties assessment methods

One author (QAK) independently reviewed qualifying studies for methodological quality based on their study design. An independent second author (SDA) conducted further independent verification. Any disagreements among the reviewers (QAK and SDA) were resolved using consensus.

For observational studies, we used the Newcastle-Ottawa Scale (NOS) quality assessment for cohort (37) and cross-sectional studies (38) using the star rating system, where each study was evaluated for sample selection, comparability of the groups and the outcome assessment. The scores translate into an overall rating of good, fair or poor study quality using the Agency for Health care Research and Quality (AHRQ)-developed thresholds. Studies were considered as good quality if they have 3 or 4 stars in the selection domain AND 1 or 2 stars in the comparability domain AND 2 or 3 stars in the outcome/exposure domain, they were considered as fair quality if they have 2 stars in selection domain AND 1 or 2 stars in comparability domain AND 2 or 3 stars in outcome/exposure domain, and they were considered as poor quality if they have 0 or 1 star in selection domain OR 0 stars in comparability domain OR 0 or 1 stars in outcome/exposure domain (39). For cross-sectional studies, studies that scored a total of 9–10 points were considered as very good studies, those with 7–8 points were considered as good studies, those with 5–6 points were considered as moderate studies, and those with 4 points or less were considered as unsatisfactory studies.

The Joanna Briggs Institute Critical Appraisal Tools was used to assess the quality of randomized controlled studies (40) and quasi experimental studies (41). Studies were categorized as “high quality” if they met at least 75% of these standards, “moderate” if they met between 50 and 75% of relevant standards, and “low” if <50%.

The 5-point Mixed Method Appraisal Tool was also used to assess the quality of mixed method studies (42). Studies were categorized as “high quality” if they met at least 75% of these standards, “moderate” if they met between 50 and 75% of relevant standards, and “low” if <50%.

Furthermore, we evaluated the quality of standard measurement properties using the Consensus-Based Standards for the Selection of Health Measurement Instruments (COSMIN) checklist (43). Since the COSMIN checklist is a modular tool, it may not be necessary to complete the whole checklist when evaluating the quality of studies (44). Therefore, we only assessed internal consistency and cross-cultural validity. In the COSMIN checklist assessment, we followed all translation and validation that might be done previously in small studies before these included studies. The total score is obtained by taking the lowest response option of any item per measurement property, with possible scores on a four-point scale of inadequate, doubtful, adequate, or very good.

## 3. Results

### 3.1. Studies identified

**Figure 1:**
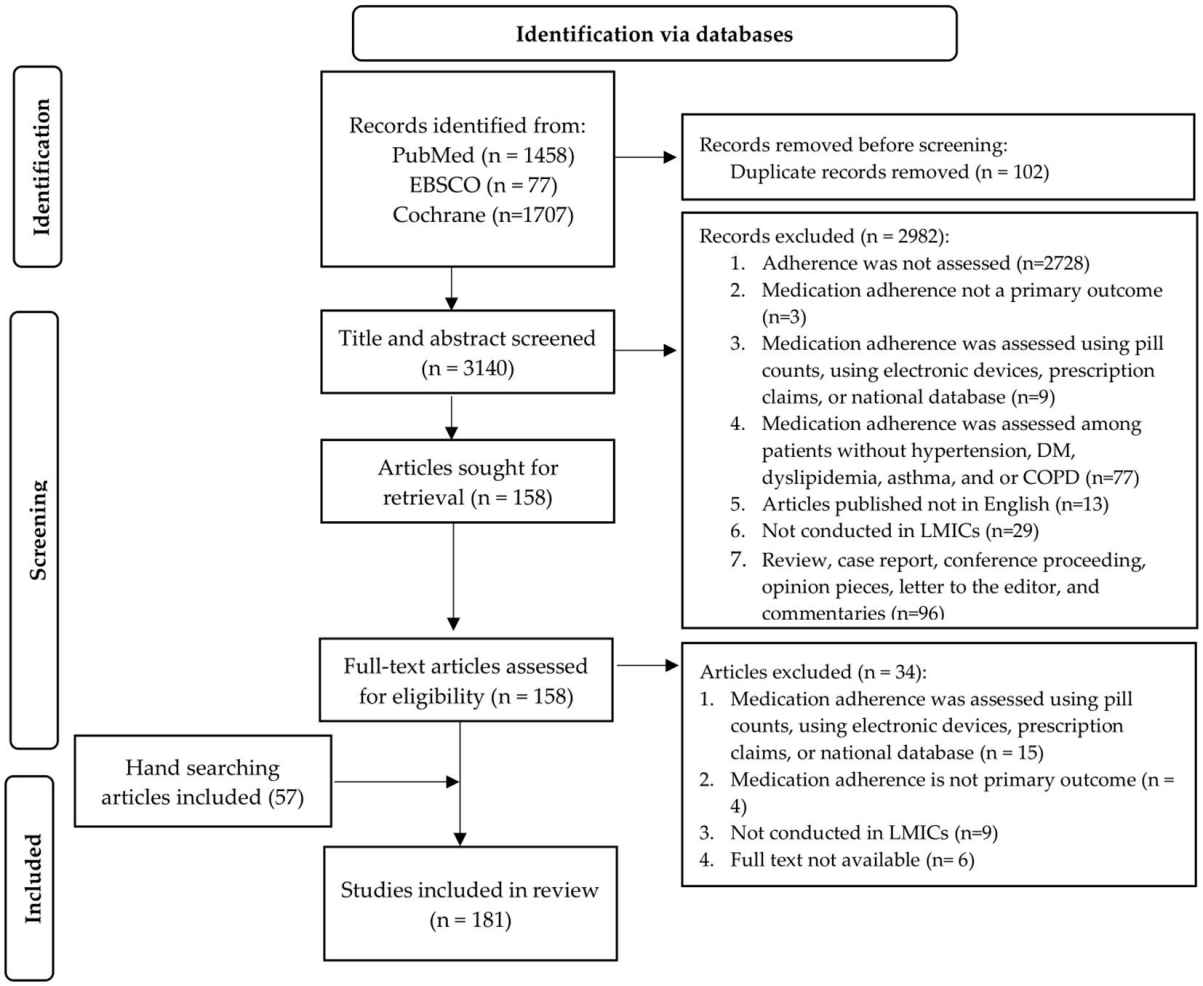
Flow diagram of the study selection process.

A total of 3,242 records were identified through the systematic search, and 102 duplicates were removed. After screening the abstracts and titles, 2,982 articles were excluded, and 158 full text articles were assessed for eligibility. Then, 57 articles were added as a result of hand-searching the literature. A total of 34 full-text articles were excluded because adherence was not assessed by self-reported instruments (*n* = 15), were not conducted in LMIC (*n* = 9), full text was not available (*n* = 6), and adherence was not the primary outcome (*n* = 4). Finally, 181 articles met the selection criteria, and were included in this scoping review. The study selection process is illustrated in a PRISMA flow chart (Figure 1).

### 3.2. Studies' general characteristics

Table 1 summarizes the general characteristics of the included studies published from 1995 (45) to 2022 (46). Half of the studies included (90/181; 49.7%) assessed medication adherence in patients with diabetes mellitus and patients with hypertension (77/181; 42.5%). Six studies assessed adherence in asthma patients, three studies assessed in COPD patients, and one study assessed it in dyslipidemia patients. Four studies assessed multiple types of chronic diseases. While most instruments were disease agnostic, three self-reported instruments were disease specific, such as the Adherence Report Scale for Asthma (MARS-A) for asthma, Self-Care Inventory (SCI) for diabetes mellitus, and Therapeutic Adherence Scale for Hypertensive Patients (TASHP) for hypertension.

**Table 1:** Study characteristics (total N = 181).

**Characteristic**	**Number of studies**	**Percentage (%)**
**Study design**		
Cross sectional	160	88.4
Randomized controlled trial (RCT)	15	8.3
Quasi experimental	3	1.7
Cohort	2	1.1
Mixed method study	1	0.6
**Study period**		
N/A	19	10.5
1–3 months	65	35.9
4–6 months	55	30.4
7–9 months	18	9.9
10–12 months	13	7.2
>12 months	11	6.1
**Type of self-reported instruments**		
ARMS	1	0.6
BMQ	2	1.1
DAI	5	2.8
Hill-Bone Medication Adherence Scale	8	4.4
MALMAS	1	0.6
MARS-A	1	0.6
MARS-10	4	2.2
MARS-5	5	2.8
MASES	1	0.6
MASES-SF	1	0.6
MTA	4	2.2
MAQ	2	1.1
MCQ	3	1.7
MMAS-4	45	24.9
MMAS-6	1	0.6
MMAS-7	1	0.6
MMAS-8	78	43.1
SCID	1	0.6
Self-Reported Compliance Test	1	0.6
TASHP	1	0.6
MMAS-4 and BMQ	1	0.6
MMAS-4 and MAQ	1	0.6
No Name	13	7.2
**Response rate of self-reported instrument**		
< 60%	3	1.7
>60%−70%	3	1.7
>70%−80%	7	3.9
>80%−90%	28	15.5
>90%	59	32.6
N/A	81	44.8
**Population (type of chronic disease)**		
Diabetes mellitus (DM)	90	49.7
Hypertension (HT)	77	42.5
Asthma	6	3.3
COPD	3	1.7
Dyslipidemia	1	0.6
DM and HT	3	1.7
DM, HT, and asthma	1	0.6
**Sample size**		
≤ 100	14	7.7
101–200	52	28.7
201–300	38	21.0
301–400	40	22.1
401–500	20	11.0
>500	17	9.4
**Adherence phases**		
Initiation or implementation	9	5.0
Implementation	172	95.0

ARMS, Adherence to Refills and Medications Scale; BMQ, Brief Medication Questionnaire; DAI, Drug Attitude Inventory; MALMAS, Malaysian Medication Adherence Scale; MARS-10, Medication Adherence Report Scale-10; MARS-5, Medication Adherence Report Scale-5; MASES, Medication Adherence Self-Efficacy Scale; MASES-SF, Medication Adherence Self-Efficacy Scale-Short Form; MTA, Measure Treatment Adherence; MAQ, Medication Adherence Questionnaire; MCQ, Medication Compliance Questionnaire; MMAS-4, Morisky Medication Adherence Scale-4; MMAS-6, Morisky Medication Adherence Scale-6; MMAS-7, Morisky Medication Adherence Scale-7; MMAS-8, Morisky Medication Adherence Scale-8; SCID, Self Care Inventory Diabetes; TASHP, Therapeutic Adherence Scale For Hypertensive Patients.

**Figure 2:**
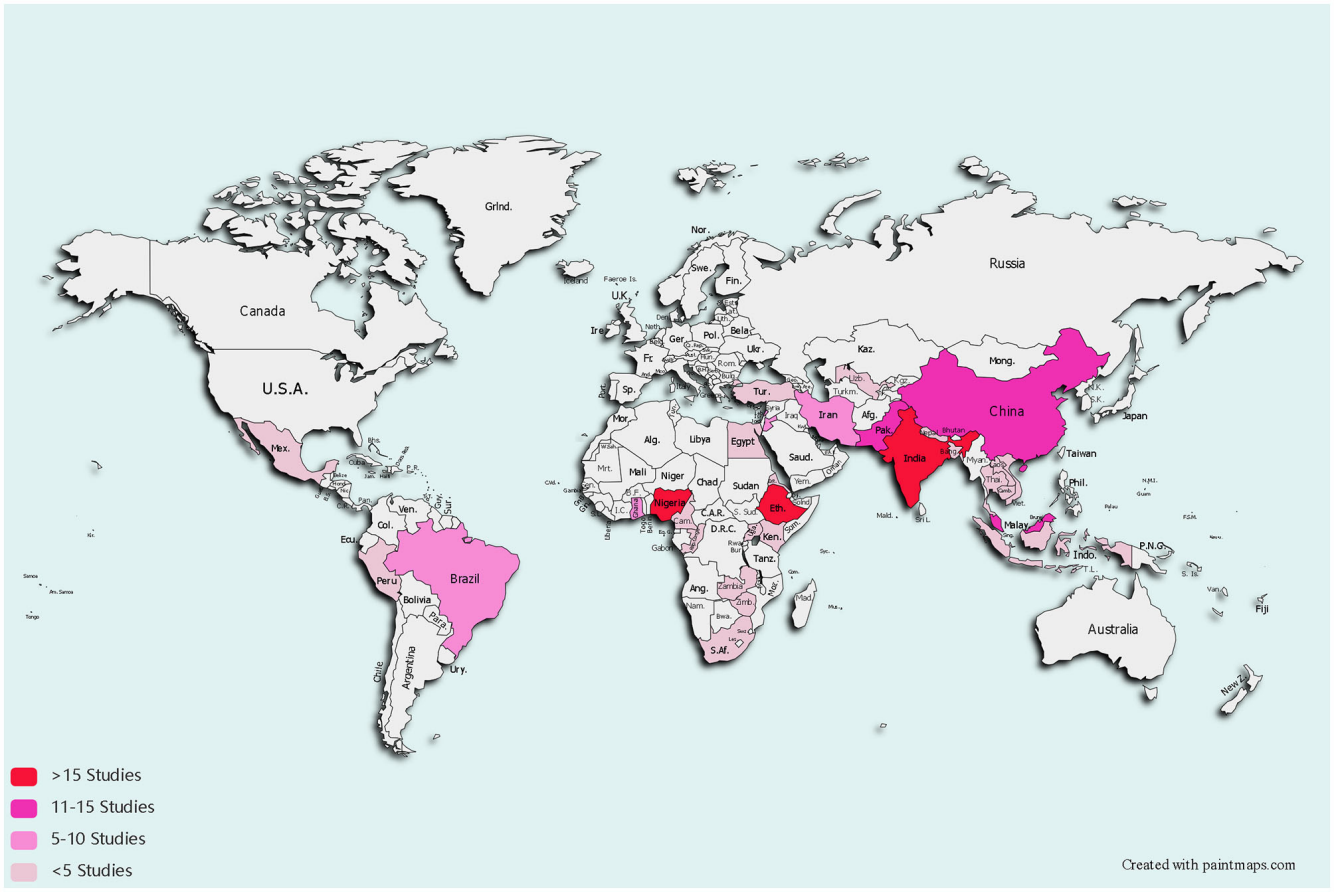
Low middle income countries where self-reported medication adherence instruments were studied.

The majority of studies were conducted in Ethiopia (27/181), India (17/181), and Nigeria (16/181) (Supplementary material 3). Two studies were conducted in two countries simultaneously, such as in Lebanon and Jordan or Ghana and Nigeria. Figure 2 shows the country coverage of the included studies as well as the number of studies. A pink gradient color represents the number of studies, a light color suggests a small number of studies, and the darker the color gradation indicates the greater the number of studies.

### 3.3. Self-reported instruments' use, content, and quality in LMIC

We reviewed 181 eligible studies that focused on self-reported instruments to assess medication adherence in patients living in LMIC with hypertension, diabetes mellitus, dyslipidemia, asthma, or COPD. The sample sizes of the studies ranged from 29 (47) to 1,698 (48). The study period of the studies varied widely, ranging from 1 month (49–52) to 3 years (48). Almost half of the studies included (81/181; 44.8%) did not report a response rate. Of the reported response rate, the rate ranged from 50.8% (49) to 100% (46, 50, 53–59).

Nine studies included people in the initiation and implementation phase of medication adherence (60–68) and the remaining part only included the implementation phase of medication adherence. No studies assessed discontinuation of medication (Supplementary material 4).

Of the 181 studies included, 15 studies performed across 10 countries used LMIC developed self-reported instruments, and the remaining 166 studies (across 32 countries) in LMICs applied existing self-reported instruments. A total of 32 distinct types of self-reported instruments to assess medication adherence were identified (Supplementary material 5). The most common self-reported adherence instruments to assess medication adherence were the MMAS-8 (78/181; 43.1%) and the MMAS-4 (45/181; 24.9%) (Supplementary material 6). Two studies were conducted using a combination of self-reported adherence instruments, such as a combination of the MMAS-4 and BMQ or a combination of MMAS-4 and MAQ. Of the 32 self-reported instruments identified, there were 14 self-reported instruments developed by LMICs (there are two studies utilizing the same instrument), while the other 18 self-reported instruments were adapted from original self-reported instruments developed in HICs (Supplementary material 8).

### 3.4. Self-reported adherence instruments' applicability in LMIC

A significant challenge regarding the self-reported instrument applicability in LMICs was that only five studies developed self-reported instruments with modifications for the local context or for the native population in LMICs or patients with low literacy in LMICs (69–73). Additionally, there were six studies using adapted self-reported instruments from HIC to address issues in LMIC such as financial barriers and access to care (64, 74–78) (Supplementary material 7).

Regarding patient related factors, it was shown that just under 25% of self-reported instruments assessed traveling and financial issues. There was no adapted self-reported from HIC that assessed the use of traditional medicine and only 7.1% of developed self-reported instruments from LMICs assessed the use of traditional medicine.

Healthcare provider and system related factors demonstrated a low percentage of self-reported instruments considering lack of communication with healthcare provider, access to care, and running out of medication.

**Figure 3:**
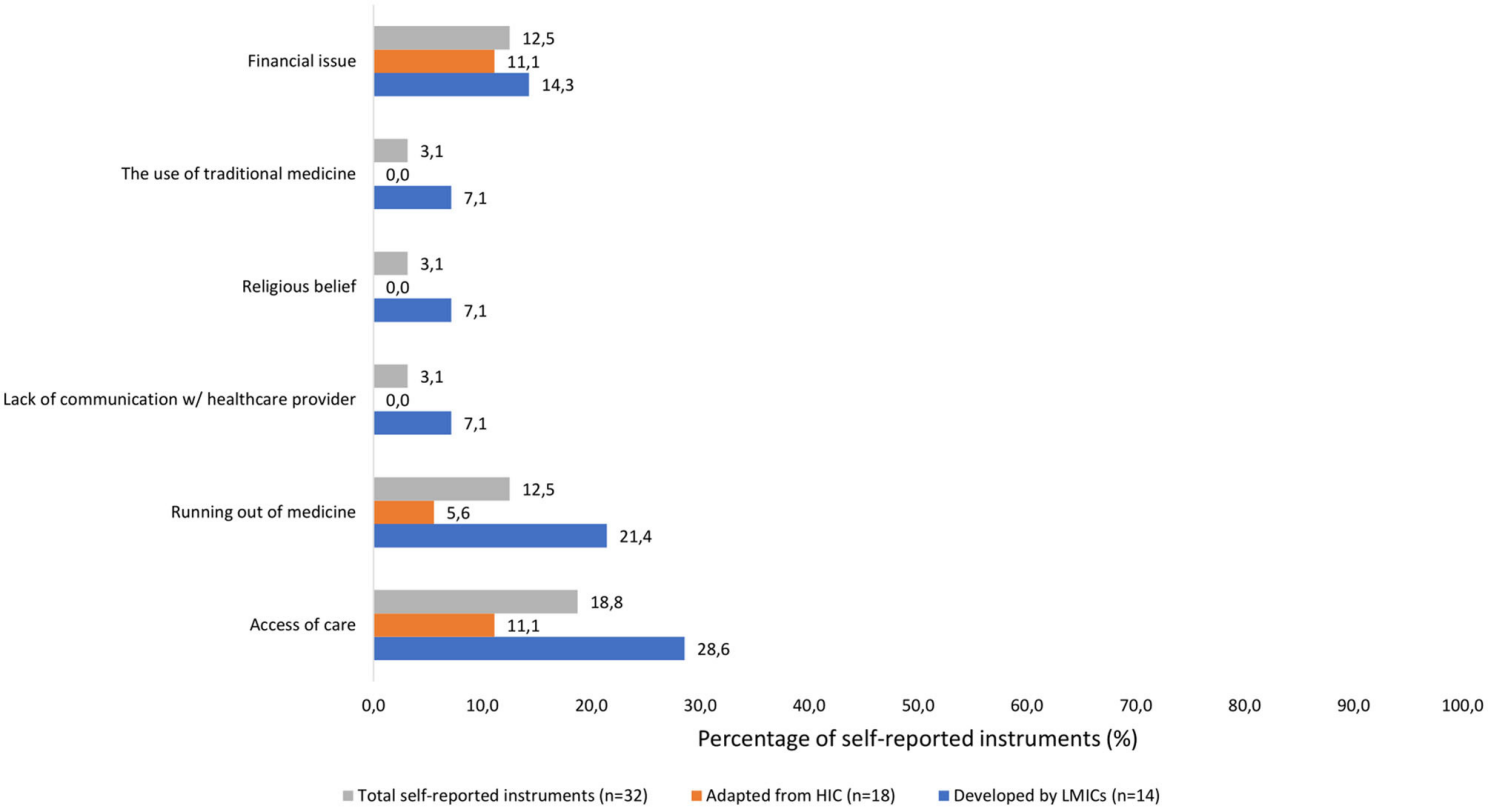
LMIC related medication adherence issues.

Similarly, social factors represented < 25%. None of the adapted self-reported measures from HICs examined religious beliefs as a reason for non-adherence, and only 7.1% of the developed self-reported instruments from LMICs did so (Figure 3).

### 3.5. Methodological quality properties

All studies included that used cohort, quasi, and mixed-method design studies were considered as good category according to the quality appraisal checklist (Supplementary material 9). Twelve randomized controlled trials were considered as high quality while three other RCTs were considered as moderate quality. The majority of the cross-sectional studies were considered as moderate quality (65 studies) and good quality (58 studies). There were 37 cross sectional studies that were considered as unsatisfactory quality since there were no descriptions of sampling strategy, no justification for the sample size, no description of the response rate, and no validated measurement tool.

**Figure 4:**
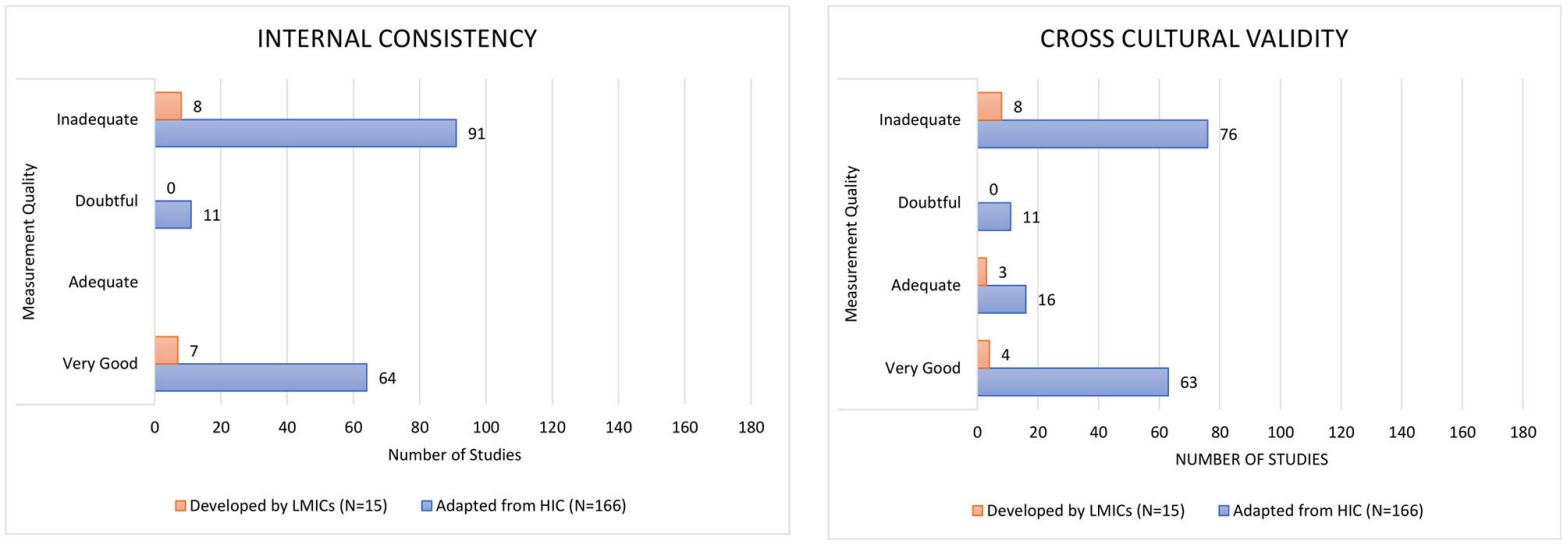
Methodological quality of included studies.

According to the COSMIN checklist, half of the studies were rated as inadequate for internal consistency (54.7%) since there were no reported Cronbach alpha values or item-total correlation calculated (Figure 4). Nearly half of the studies were rated inadequate for cross-cultural validation (46.4%) as samples were not similar regarding relevant characteristics across groups or the approach was not appropriate, i.e., they did not follow the guidelines developed for translation and cross-cultural adaptation questionnaires (79, 80). There were four studies which verbally translated the instrument into the local language for some of the participants, when needed (Supplementary material 10).

### 3.6. Fee applied for using self-reported instruments in LMICs

One hundred twenty-six studies (70%) identified the license fee requirement of the self-reported instrument as a challenge. Among the self-reported instruments that request extra fees for license are the MMAS-8 (78/181, 43.1%), MMAS-4, (47/181; 26.0%), and ARMS (1/181; 0.6%).

## 4. Discussion

### 4.1. Main findings

Most of the 32 self-reported adherence instruments applied in 181 LMIC studies include some patient, medication, healthcare provider and system, and societal factors related to non-adherence. HICs and LMICs both face a wide range of challenges, though the specific issues they encounter can differ quite significantly. Some of the challenges that are common in both HICs and LMICs include being busy, memory difficulties, traveling, lack of knowledge, low necessity, high concern, polypharmacy, and experiencing adverse effects. In LMICs, there is often a greater emphasis on the use of traditional medicine, religious belief, and lack of communication with the healthcare provider. Additionally, financial issues, access to care, and running out of medicines may act as barrier to medication adherence in LMICs. However, these LMIC specific issues are only represented in the 14 identified self-reported instruments developed in LMICs. Notably, still 166 out of the 181 studies (91.7%) in LMICs applied an instrument developed in a HIC with just over half of studies (99/181 studies, 54.7%) having inadequate internal consistency and almost half (84/181 studies, 46.4%) having inadequate cross cultural validation. Around 70% of the studies indicated that a fee applied for using the instrument.

### 4.2. Interpretation

Our findings highlight the differences in applicability of self-reported adherence instruments in HIC vs. LMIC. Indeed, some problems, such as financial hardship, usually occur more often in LMICs than in HICs, and do significantly contribute to medication non-adherence (81, 82). Despite the fact that these were among the most frequent risk factors for non-adherence in LMICs, only a few instruments have incorporated this issue. Financial issues relate to transportation costs and other basic living expenses like housing, food, and school cost, all competing with the cost of medical treatment (83). In several previous studies it has been reported that patients with low income are more at risk for non-adherence than those with high income (84–86). Even though patients with low income in HICs may have similar relative risk, its absolute impact is higher in LMICs given the proportion of people live in poverty in LMICs is higher than in HICs and social security systems are less developed (87).

Another LMIC specific issue involved traditional medicines that were used by a large portion of the population in LMICs (88), and this has contributed to medication non-adherence (81). Using traditional medicine has become a growing phenomenon because most people believe contemporary medication is ineffective and harmful to their kidneys (89, 90). As a result, people tend to refuse conventional medication and prefer using traditional herbals. Indeed, traditional medicine use is prevalent in LMICs as well as in HICs, with 80% of people in the world utilizing traditional medicine (91). However, health-seeking behaviors regarding traditional medicine use differ between HICs and LMICs (92, 93). In HICs, traditional medicines are utilized as a supplemental therapy to conventional medicine, whereas in LMICs, traditional medicines are often the primary treatment (94). Moreover, multiple studies in LMIC settings have demonstrated that using traditional medicine might be a barrier to seek for treatment in the conventional healthcare system (95–97).

In LMICs, religion plays a significant role in daily life and it could be a challenging issue regarding non-adherence to medication due to the beliefs about the super power of God (98). People who place a high value on religion tend to share the view that God can and does perform miracles and has absolute control over everything in their lives (99). Misinterpretation of religious beliefs has generated the belief that religion-related healing is superior to conventional medication. Several studies in LMICs have found that religiosity can be a barrier to medication adherence. For example, patients with chronic diseases stop taking their medication because they believe their pastors' prayers will cure them (100, 101).

LMIC studies also showed that poor communication of healthcare providers with patients was linked to low medication adherence (102, 103). Inadequate and infrequent patient-provider contact and patient education regarding the medication affected the understanding of many patients. These patients claimed that during consultations, their doctors did not inquire about medication adherence or provided insufficiently detailed instructions on how to take their prescriptions (104). Therefore, communication between the health professional and the patient during the course of the medical encounter should play a critical role in improving medication adherence.

Several studies conducted in LMICs reported “running out of medicine” as reason for non-adherence (105–107). In LMICs, it is often found that lack of access and affordability to medicines is a barrier to good health. Several gaps in local health systems impede the delivery of medicines to millions of people including purchasing procedures, tax and tariff laws, markups along the supply chain, and the poor effectiveness of national drug regulatory agencies (108). A systematic review has shown the scarce availability and affordability of essential medicines for chronic diseases in some LMIC with many not reaching the WHO target of 80% availability (109). A possible explanation for running out of medicines was caused by the limited availability of medication in primary health care (105). Patients were supposed to get all their prescription drugs from primary care and most of them refused to go to pharmacies to pay out-of-pocket (107). Lack of access to healthcare is frequently used as an euphemism for low utilization of available services, which is often found in LMICs (110). Patients who do not have easy access to healthcare may be less likely to follow their treatment plan and less likely to adhere to their medications as prescribed (111).

While content of many self-reported instruments, i.e., the inclusion of LMIC specific barriers, was already deemed a shortcoming, also several contextual and process factors that require attention were identified. For example, only half of the studies reported a translation process when relevant, whereas a translation process based on guidelines is required for those translated to a local language (112). The translation process is critical in order to maintain conceptual, content, semantic, and construct equivalences between the two languages and cultures, which is required to get credible measurement results (113). Translation and cultural adaptation would guarantee that the questionnaire's responses are reflected and analyzed in a consistent manner (79). This is particularly relevant for LMICs since the self-reported instruments are often the only source of adherence information.

Beyond translation, we observed that only a small number of self-reported adherence instruments have been validated. Furthermore, psychometric properties when adapting or developing self-reported instruments in LMICs were often not reported. When a study does not have sufficient psychometric properties, the results of the study cannot be trusted, and the quality of the self-reported adherence instrument remains unclear (44). It has been noted in a recent systematic review that in almost half of included studies, the existing self-reported adherence instruments lack sufficient evidence to meet validity criteria (114). Moreover, no reporting of response rates or having a low response rate were observed in almost a half of included studies, which could increase the likelihood of selection bias and decrease the external validity (115). Indeed, the true prevalence of non-adherence may be underestimated if there is a high rate of non-response, especially if the non-response is associated with the outcome or if non-responders significantly differ from responders (116).

Finally, many self-reported adherence instruments require fees in exchange for licenses to use them in research. This hampers more adherence research in low-resource settings where these fees are unaffordable. At the same time, LMIC heavily rely on self-reported instruments in absence of for example electronic monitoring of adherence using more advanced technologies. Having self-reported instruments available free of charge is therefore essential.

### 4.3. Strengths and limitations

Some strengths and limitations of our review should be mentioned. This is the first systematic literature review analyzing the use of self-reported adherence instruments and their applicability in LMICs. It could guide selection of adherence self-reported instruments most relevant for measuring medication adherence in LMICs. This review extends previous literature on adherence and emphasizes the challenges of implementing these self-reported instruments in LMICs. A limitation is that potential publication bias may exist due to the exclusion of studies not published in English and limited inclusion of gray literature. Also, while three databases were extensively searched, some studies may have not been included in these three databases. Furthermore, due to the broad inclusion criteria, significant heterogeneity in study design, duration and sample size was found, making direct comparisons between studies challenging.

### 4.4. Recommendations for future research and practice

Our findings highlight that it is necessary to develop an adherence self-reported instrument that can be adapted to the local context, the health systems and resources available in LMIC in order to precisely and accurately capture medication non-adherence (117). As such, healthcare providers can obtain better insight into potential non-adherence issues and address them during patient counseling to avoid complications of diseases and unnecessary costs.

## 5. Conclusion

The applicability of self-reported instruments in LMICs was deemed suboptimal. Main issues that need improvement: the inclusion of LMICs relevant issues should be increased, proper translation into the local language and formal cross-cultural validation should be performed, and fees applied for using self-reported instruments in LMICs should be lowered or removed. Nonetheless, because of the methodological shortcomings observed in some included studies, the findings of this study call for the development of a well-validated self-reported adherence instrument that can be universally applied to context, health systems and resources in LMICs.

## Data availability statement

The original contributions presented in the study are included in the article/Supplementary material, further inquiries can be directed to the corresponding author.

## Author contributions

QAK conducted the literature searches and wrote the first draft of this manuscript. QAK and SDA conducted screening and data extraction. QAK, SDA, JvB, and RA contributed to the revision of the manuscript. All authors contributed to development of the review protocol, interpretation of findings, revising the manuscript, and approved the final manuscript.
