# Supplementary material for: Self-reported medication adherence instruments and their applicability in low-middle income countries: a scoping review
Source: Front Public Health. 2023 Jul 13;11:1104510. doi: 10.3389/fpubh.2023.1104510 (PMC10374330; doi:10.3389/fpubh.2023.1104510)
Supplement: Supplementary file 1 [file Data_Sheet_1.PDF]

Supplementary 1. PRISMA-SCR Checklist

Preferred Reporting Items for Systematic reviews and Meta-Analyses extension for Scoping Reviews (PRISMA-ScR) Checklist

SECTION	ITEM	PRISMA-ScR CHECKLIST ITEM	REPORTED ON PAGE #
**TITLE**			
Title	1	Identify the report as a scoping review.	Page 1
**ABSTRACT**			
Structured summary	2	Provide a structured summary that includes (as applicable): background, objectives, eligibility criteria, sources of evidence, charting methods, results, and conclusions that relate to the review questions and objectives.	Page 2
**INTRODUCTION**			
Rationale	3	Describe the rationale for the review in the context of what is already known. Explain why the review questions/objectives lend themselves to a scoping review approach.	Page 4
Objectives	4	Provide an explicit statement of the questions and objectives being addressed with reference to their key elements (e.g., population or participants, concepts, and context) or other relevant key elements used to conceptualize the review questions and/or objectives.	Page 5
**METHODS**			
Protocol and registration	5	Indicate whether a review protocol exists; state if and where it can be accessed (e.g., a Web address); and if available, provide registration information, including the registration number.	Page 5
Eligibility criteria	6	Specify characteristics of the sources of evidence used as eligibility criteria (e.g., years considered, language, and publication status), and provide a rationale.	Page 5
Information sources*	7	Describe all information sources in the search (e.g., databases with dates of coverage and contact with authors to identify additional sources), as well as the date the most recent search was executed.	Page 5
Search	8	Present the full electronic search strategy for at least 1 database, including any limits used, such that it could be repeated.	Page 5
Selection of sources of evidence†	9	State the process for selecting sources of evidence (i.e., screening and eligibility) included in the scoping review.	Page 6

SECTION	ITEM	PRISMA-ScR CHECKLIST ITEM	REPORTED ON PAGE #
Data charting process‡	10	Describe the methods of charting data from the included sources of evidence (e.g., calibrated forms or forms that have been tested by the team before their use, and whether data charting was done independently or in duplicate) and any processes for obtaining and confirming data from investigators.	Page 6
Data items	11	List and define all variables for which data were sought and any assumptions and simplifications made.	Page 6
Critical appraisal of individual sources of evidence§	12	If done, provide a rationale for conducting a critical appraisal of included sources of evidence; describe the methods used and how this information was used in any data synthesis (if appropriate).	NA
Synthesis of results	13	Describe the methods of handling and summarizing the data that were charted.	Page 7
**RESULTS**			
Selection of sources of evidence	14	Give numbers of sources of evidence screened, assessed for eligibility, and included in the review, with reasons for exclusions at each stage, ideally using a flow diagram.	Page 7
Characteristics of sources of evidence	15	For each source of evidence, present characteristics for which data were charted and provide the citations.	Page 9
Critical appraisal within sources of evidence	16	If done, present data on critical appraisal of included sources of evidence (see item 12).	NA
Results of individual sources of evidence	17	For each included source of evidence, present the relevant data that were charted that relate to the review questions and objectives.	Page 10
Synthesis of results	18	Summarize and/or present the charting results as they relate to the review questions and objectives.	Page 10-13
**DISCUSSION**			
Summary of evidence	19	Summarize the main results (including an overview of concepts, themes, and types of evidence available), link to the review questions and objectives, and consider the relevance to key groups.	Page 13-16
Limitations	20	Discuss the limitations of the scoping review process.	Page 16
Conclusions	21	Provide a general interpretation of the results with respect to the review questions and objectives, as well as potential implications and/or next steps.	Page 16
**FUNDING**			

SECTION	ITEM	PRISMA-ScR CHECKLIST ITEM	REPORTED ON PAGE #
Funding	22	Describe sources of funding for the included sources of evidence, as well as sources of funding for the scoping review. Describe the role of the funders of the scoping review.	Page 17

Supplementary 2. Search Term Strategies

Database	Search Terms
**PubMed**	• **Concept 1 = Self-Report Questionnaire**<br>"self report*"[MeSH Terms] OR "self-report questionnaire"[Title/Abstract] OR "self-report questionnaire"[Text Word] OR "patient reported outcome measures*"[Title/Abstract] OR "patient reported outcome measures*"[Text Word] OR "self-report instrument"[Title/Abstract] OR "self-report instrument"[Text Word] OR "self-report scale"[Title/Abstract] OR "self-report scale"[Text Word] OR "self-report survey"[Title/Abstract] OR "self-report survey"[Text Word]<br><br>• **Concept 2 = Medication Adherence**<br>"Medication Adherence"[MeSH Terms] OR "Medication Adherence"[Title/Abstract] OR "Medication Adherence"[Text Word] OR "Medication Nonadherence"[Title/Abstract] OR "Medication Nonadherence"[Text Word] OR "Medication Persistence"[Title/Abstract] OR "Medication Persistence"[Text Word] OR "Medication Compliance"[Title/Abstract] OR "Medication Compliance"[Text Word] OR "Drug Compliance"[Title/Abstract] OR "Drug Compliance"[Text Word] OR "Patient compliance"[MeSH Terms] OR "Patient compliance"[Title/Abstract] OR "Patient compliance"[Text Word] OR "complian*"[Title/Abstract] OR "noncomplian*"[Title/Abstract] OR "adher*"[Title/Abstract] OR "nonadher*"[Title/Abstract] OR "comply"[Title/Abstract] OR "complies"[Title/Abstract] OR "complying"[Title/Abstract] OR "concordance"[Title/Abstract] OR "discontinu*"[Title/Abstract] OR "continu*"[Title/Abstract] OR "persist*"[Title/Abstract] OR "treatment refusal"[Title/Abstract]<br><br>• **Concept 3 = Chronic Disease**<br>"Chronic Disease"[Mesh] OR "Chronic Disease"[tiab] OR "Hypertension" [Mesh] OR "Hypertension" [tiab] OR "Diabetes Mellitus" [Mesh] OR "Diabetes Mellitus" [tiab] OR "Hyperlipidemias" [Mesh] OR "Hyperlipidemia"[tiab] OR "asthma"[Mesh] OR "asthma"[tiab] OR "COPD"[tiab] OR "chronic obstructive pulmonary disease"[tiab]<br><br>• **Concept 4 = Low Middle Income Countries**<br>"Developing Countries"[Mesh] OR "Developing

	Countries"[Title/Abstract] OR "developing" [Title/Abstract] OR "low middle income countries"[Title/Abstract] OR "Third world countries"[Title/Abstract] "low resources countries" [Title/Abstract] OR "limited resource" [Title/Abstract] OR "low resource" [Title/Abstract] OR "constrained resource" [Title/Abstract] OR "restricted resource" [Title/Abstract] OR "emerging country" OR "emerging countries" [Title/Abstract] OR "Africa" [Title/Abstract] OR "Asia, Southeastern" [Mesh] OR "Asia, Central" [Mesh] OR "Asia, Western" [Mesh] OR "Mongolia"[Mesh] OR "Caribbean Region"[Mesh] OR "Central America"[Mesh] OR "South America"[Mesh] OR "Transcaucasia"[Mesh] OR "Europe, Eastern"[Mesh] OR "Pacific Islands"[Mesh] OR "Afghan*"[Title/Abstract] OR "Guinea*"[Title/Abstract] OR "Peru" [Title/Abstract] OR "Alban*"[Title/Abstract] OR "Philippin*"[Title/Abstract] OR "Algeria*"[Title/Abstract] OR "Haiti*"[Title/Abstract] OR "Romania*"[Title/Abstract] OR "Samoa*"[Title/Abstract] OR "Hondura*"[Title/Abstract] OR "Rwand*"[Title/Abstract] OR "Angola*"[Title/Abstract] OR "Hungar*"[Title/Abstract] OR "Argentin*"[Title/Abstract] OR "India"[Title/Abstract] OR "Sao Tome"[Title/Abstract] OR "Armeni*"[Title/Abstract] OR "Indonesia*"[Title/Abstract] OR "Senegal*"[Title/Abstract] OR "Azerbaijan"[Title/Abstract] OR "Iran*"[Title/Abstract] "Serbia*"[Title/Abstract] OR "Banglad*"[Title/Abstract] OR "Iraq*"[Title/Abstract] OR "Seychell*"[Title/Abstract] OR "Belarus*"[Title/Abstract] OR "Jamaic*"[Title/Abstract] OR "Sierra Leon*"[Title/Abstract] OR "Beliz*"[Title/Abstract] OR "Jordan*"[Title/Abstract] OR "Solomon"[Title/Abstract] OR "Benin*"[Title/Abstract] OR "Kazakhstan*"[Title/Abstract] OR "Somalia*"[Title/Abstract] OR "Bhutan*"[Title/Abstract] OR "Kenya*"[Title/Abstract] OR "South Africa*"[Title/Abstract] OR "Bolivia*"[Title/Abstract] OR "Kiribati"[Title/Abstract] OR "South Sudan*"[Title/Abstract] OR "Bosnia*"[Title/Abstract] OR "Herzegovina"[Title/Abstract] OR "Korea*"[Title/Abstract] OR "Sri Lank*"[Title/Abstract] OR "Bostwan*"[Title/Abstract] OR "Kosovo"[Title/Abstract] OR "St.Lucia"[Title/Abstract] OR "Brazil*"[Title/Abstract] OR "Kyrgyz*"[Title/Abstract] OR "St. Vincent"[Title/Abstract] OR "Grenadin*"[Title/Abstract] OR "Bulgaria*"[Title/Abstract] OR "Burkina Faso"[Title/Abstract] OR "Burund*"[Title/Abstract] OR "Cabo Verde"[Title/Abstract] OR "Cambodia*"[Title/Abstract] OR "Cameroon*"[Title/Abstract] OR "Central Africa*"[Title/Abstract] OR "Chad"[Title/Abstract] OR "China"[Title/Abstract] OR "Colombia*"[Title/Abstract] OR "Comoros*"[Title/Abstract] OR "Congo"[Title/Abstract] OR "Costa Rica"[Title/Abstract] OR "Cote D'ivoire"[Title/Abstract] OR "Cuba"[Title/Abstract] OR "Djibouti"[Title/Abstract] OR "Dominica*"[Title/Abstract] OR "Ecuador*"[Title/Abstract] OR

	"Egypt*"[Title/Abstract] OR "El Salvador*"[Title/Abstract] OR "Equatorial Guinea" [Title/Abstract] OR "Eritrea"[Title/Abstract] OR "Eswatini"[Title/Abstract] OR "Ethiopia"[Title/Abstract] OR "Fiji"[Title/Abstract] OR "Gabon"[Title/Abstract] OR "Gambia"[Title/Abstract] OR "Ghana"[Title/Abstract] OR "Grenada"[Title/Abstract] OR "Guatemala"[Title/Abstract] OR "Guinea"[Title/Abstract] OR "Guinea-bissau"[Title/Abstract] OR "Guyana"[Title/Abstract] OR "Lao"[Title/Abstract] OR "Lebanon"[Title/Abstract] OR "Lesotho"[Title/Abstract] OR "Liberia"[Title/Abstract] OR "Libya"[Title/Abstract] OR "Madagascar"[Title/Abstract] OR "Malawi"[Title/Abstract] OR "Malaysia"[Title/Abstract] OR "Maldives"[Title/Abstract] OR "Mali"[Title/Abstract] OR "Marshall island*"[Title/Abstract] OR "Mauritan*"[Title/Abstract] OR "Mauritiu*"[Title/Abstract] OR "Mexico"[Title/Abstract] OR "Micronesia"[Title/Abstract] OR "Moldova*"[Title/Abstract] OR "Montenegro"[Title/Abstract] OR "Morocco"[Title/Abstract] OR "Mozambique"[Title/Abstract] OR "Myanmar"[Title/Abstract] OR "Namibia"[Title/Abstract] OR "Nepal"[Title/Abstract] OR "Nicaragua"[Title/Abstract] OR "Niger*"[Title/Abstract] OR "North macedonia"[Title/Abstract] OR "Pakistan"[Title/Abstract] OR "Panama"[Title/Abstract] OR "Papua new guinea"[Title/Abstract] OR "Paraguay"[Title/Abstract] OR "Russia*"[Title/Abstract] OR "Suriname"[Title/Abstract] OR "Syrian*"[Title/Abstract] OR "Tajikist*"[Title/Abstract] OR "Tanzania*"[Title/Abstract] OR "Thailand"[Title/Abstract] OR "Timor-leste"[Title/Abstract] OR "Togo"[Title/Abstract] OR "Tonga"[Title/Abstract] OR "Tunisia"[Title/Abstract] OR "Turkey"[Title/Abstract] OR "Turkmenistan"[Title/Abstract] OR "Tuvalu"[Title/Abstract] OR "Uganda"[Title/Abstract] OR "Ukraine"[Title/Abstract] OR "Uzbekistan"[Title/Abstract] OR "Vanuatu"[Title/Abstract] OR "Vietnam"[Title/Abstract] OR "West bank and gaza"[Title/Abstract] OR "Yemen"[Title/Abstract] OR "Zambia"[Title/Abstract] OR "Zimbabwe"[Title/Abstract]
**EBSCO**	• **Concept 1 = Self-Report Questionnaire**<br>TI ("Self-report questionnaire" OR "Patient Report Outcome Measure" OR "self-report instrument" OR "self-report scale" OR "self-report survey") OR AB ("Self-report questionnaire" OR "Patient Reported Outcome Measure" OR "self-report instrument" OR "self-report scale" OR "self-report survey") OR TX ("Self-report questionnaire" OR "Patient Reported Outcome Measure" OR "self-report instrument" OR "self-report scale" OR "self-report survey")<br><br>• **Concept 2 = Medication Adherence**

	TI (“Medication Adherence” OR “Medication Nonadherence” OR “Medication Persistence” OR “Medication Compliance” OR “Drug Compliance” OR “Medication Nonadherence “ OR “Patient compliance” OR "complian*" OR "noncomplian*" OR "adher*" OR "nonadher*" OR "comply" OR "complies" OR "complying" OR "concordance" OR "proportion of days covered" OR "medication possession ratio" OR "possession rate*" OR "refill" OR "discontinu*" OR "continu*" OR "persist*" OR "treatment refusal" OR "switch*" OR "addition" OR "medication gap*" OR "treatment gap*" OR "day gap*" OR "gaps refill*" OR "daily polypharmacy possession ratio*" OR "MPR" OR "PDC" OR "medication acquisition") OR AB (“Medication Adherence” OR “Medication Nonadherence” OR “Medication Persistence” OR “Medication Compliance” OR “Drug Compliance” OR “Medication Nonadherence “ OR “Patient compliance” OR "complian*" OR "noncomplian*" OR "adher*" OR "nonadher*" OR "comply" OR "complies" OR "complying" OR "concordance" OR "proportion of days covered" OR "medication possession ratio" OR "possession rate*" OR "refill" OR "discontinu*" OR "continu*" OR "persist*" OR "treatment refusal" OR "switch*" OR "addition" OR "medication gap*" OR "treatment gap*" OR "day gap*" OR "gaps refill*" OR "daily polypharmacy possession ratio*" OR "MPR" OR "PDC" OR "medication acquisition")<br><br>• **Concept 3 = Chronic Disease**<br>TI (“Chronic Disease” OR “Hypertension” OR “Diabetes Mellitus” OR “Hyperlipidemia” OR “asthma” OR “COPD” OR “Chronic obstructive pulmonary disease”) OR AB (“Chronic Disease” OR “Hypertension” OR “Diabetes Mellitus” OR “Hyperlipidemia” OR “asthma” OR “COPD” OR “Chronic obstructive pulmonary disease”)<br><br>• **Concept 4 = Low Middle Income Countries**<br>TI (“Developing Countries” OR “low middle income countries” OR “Third world countries” OR “low resources countries” OR “limited resource” OR “low resource” OR “constrained resource” OR “restricted resource” OR “delimited resource” OR “restrained resource” OR “emerging country” OR “emerging countries” OR “Africa” OR “Asia, Southeastern” OR “Asia, Central” OR “Asia, Western” OR “Mongolia” OR “Caribbean Region” OR “Central America” OR “South America” OR “Transcaucasia” OR “Europe, Eastern” OR “Pacific Islands” OR “Afghan*” OR “Guinea* OR “Peru” OR “Alban*” OR “Philippin*” OR “Algeria*” OR “Haiti*” OR “Romania*” OR “Samoa*” OR “Hondura*” OR “Rwand*” OR “Angola*” OR “Hungar*” OR “Argentin*” OR “India” OR

	“Sao Tome” OR “Armeni*” OR “Indonesia*” OR “Senegal*” OR “Azerbaijan” OR “Iran*” “Serbia*” OR “Banglad*” OR “Iraq*” OR “Seychell*” OR “Belarus*” OR “Jamaic*” OR “Sierra Leon*” OR “Beliz*” OR “Jordan*” OR “Solomon” OR “Benin*” OR “Kazakhstan*” OR “Somalia*” OR “Bhutan*” OR “Kenya*” OR “South Africa*” OR “Bolivia*” OR “Kiribati” OR “South Sudan*” OR “Bosnia*” OR “Herzegovin” OR “Korea*” OR “Sri Lank*” OR “Bostwan*” OR “Kosovo” OR “St.Lucia” OR “Brazil*” OR “Kyrgyz*” OR “St. Vincent” OR “Grenadin*” OR “Bulgaria*” OR “Burkina Faso” OR “Burund*” OR “Cabo Verde” OR “Cambodia*” OR “Cameroon*” OR “Central Africa*” OR “Chad” OR “China” OR “Colombia*” OR “Comoros*” OR “Congo” OR “Costa Rica” OR “Cote D’ivoire” OR “Cuba” OR “Djibouti” OR “Dominica*” OR “Ecuador*” OR “Egypt*” OR “El Salvador*” OR “Equatorial Guinea” OR “Eritrea” OR “Eswatini” OR “Ethiopia” OR “Fiji” OR “Gabon” OR “Gambia” OR “Ghana” OR “Grenada” OR “Guatemala” OR “Guinea” OR “Guinea-bissau” OR “Guyana” OR “Lao” OR “Lebanon” OR “Lesotho” OR “Liberia” OR “Libya” OR “Madagascar” OR “Malawi” OR “Malaysia” OR “Maldives” OR “Mali” OR “Marshall island*” OR “Mauritan*” OR “Mauritiu*” OR “Mexico” OR “Micronesia” OR “Moldova*” OR “Montenegro” OR “Morocco” OR “Mozambique” OR “Myanmar” OR “Namibia” OR “Nepal” OR “Nicaragua” OR “Niger*” OR “North macedonia” OR “Pakistan” OR “Panama” OR “Papua new guinea” OR “Paraguay” OR “Russia*” OR “Suriname” OR “Syrian*” OR “Tajikist*” OR “Tanzania*” OR “Thailand” OR “Timor-leste” OR “Togo” OR “Tonga” OR “Tunisia” OR “Turkey” OR “Turkmenistan” OR “Tuvalu” OR “Uganda” OR “Ukraine” OR “Uzbekistan” OR “Vanuatu” OR “Vietnam” OR “West bank and gaza” OR “Yemen” OR “Zambia” OR “Zimbabwe”) OR AB (“Developing Countries” OR “low middle income countries” OR “Third world countries” OR “low resources countries” OR “limited resource” OR “low reseource” OR “constrained resource” OR “restricted resource” OR “delimited reseource” OR “restrained resource” OR “emerging country” OR “emerging countries” OR “Africa” OR “Asia, Southeastern” OR “Asia, Central” OR “Asia, Western” OR “Mongolia” OR “Caribbean Region” OR “Central America” OR “South America” OR “Transcaucaisa” OR “Europe, Eastern” OR “Pacific Islands” OR “Afghan*” OR “Guinea* OR “Peru” OR “Alban*” OR “Philippin*” OR “Algeria*” OR “Haiti*” OR “Romania*” OR “Samoa*” OR “Hondura*” OR “Rwand*” OR “Angola*” OR “Hungar*” OR “Argentin*” OR “India” OR “Sao Tome” OR “Armeni*” OR “Indonesia*” OR “Senegal*” OR “Azerbaijan” OR “Iran*” “Serbia*” OR “Banglad*” OR “Iraq*” OR “Seychell*” OR “Belarus*” OR “Jamaic*” OR

	“Sierra Leon*” OR “Beliz*” OR “Jordan*” OR “Solomon” OR “Benin*” OR “Kazakhstan*” OR “Somalia*” OR “Bhutan*” OR “Kenya*” OR “South Africa*” OR “Bolivia*” OR “Kiribati” OR “South Sudan*” OR “Bosnia*” OR “Herzegovin” OR “Korea*” OR “Sri Lank*” OR “Bostwan*” OR “Kosovo” OR “St.Lucia” OR “Brazil*” OR “Kyrgyz*” OR “St. Vincent” OR “Grenadin*” OR “Bulgaria*” OR “Burkina Faso” OR “Burund*” OR “Cabo Verde” OR “Cambodia*” OR “Cameroon*” OR “Central Africa*” OR “Chad” OR “China” OR “Colombia*” OR “Comoros*” OR “Congo” OR “Costa Rica” OR “Cote D’ivoire” OR “Cuba” OR “Djibouti” OR “Dominica*” OR “Ecuador*” OR “Egypt*” OR “El Salvador*” OR “Equatorial Guinea” OR “Eritrea” OR “Eswatini” OR “Ethiopia” OR “Fiji” OR “Gabon” OR “Gambia” OR “Ghana” OR “Grenada” OR “Guatemala” OR “Guinea” OR “Guinea-bissau” OR “Guyana” OR “Lao” OR “Lebanon” OR “Lesotho” OR “Liberia” OR “Libya” OR “Madagascar” OR “Malawi” OR “Malaysia” OR “Maldives” OR “Mali” OR “Marshall island*” OR “Mauritan*” OR “Mauritiu*” OR “Mexico” OR “Micronesia” OR “Moldova*” OR “Montenegro” OR “Morocco” OR “Mozambique” OR “Myanmar” OR “Namibia” OR “Nepal” OR “Nicaragua” OR “Niger*” OR “North macedonia” OR “Pakistan” OR “Panama” OR “Papua new guinea” OR “Paraguay” OR “Russia*” OR “Suriname” OR “Syrian*” OR “Tajikist*” OR “Tanzania*” OR “Thailand” OR “Timor-leste” OR “Togo” OR “Tonga” OR “Tunisia” OR “Turkey” OR “Turkmenistan” OR “Tuvalu” OR “Uganda” OR “Ukraine” OR “Uzbekistan” OR “Vanuatu” OR “Vietnam” OR “West bank and gaza” OR “Yemen” OR “Zambia” OR “Zimbabwe”)
**Cochrane**	• **Concept 1 = Self-Report Questionnaire**<br>MeSH descriptor: [Self Report] explode all trees OR MeSH descriptor: [Patient Reported Outcome Measures] explode all trees OR Mesh descriptor: [Self-Assessment] explode all trees OR (self-report NEXT(questionnaire* OR instrument* OR scale* OR survey*)):ti,ab,kw OR (self-report NEXT(questionnaire* OR instrument* OR scale* OR survey*)) OR (patient NEXT(report*) NEXT (outcome*) NEXT (measures*)):ti,ab,kw OR (patient NEXT(report*) NEXT (outcome*) NEXT (measures*))<br><br>• **Concept 2 = Medication Adherence**<br>MeSH descriptor: [Medication Adherence] explode all trees OR (Medication NEXT(adherence* OR nonadherence* OR persistence*)):ti,ab,kw OR (Medication NEXT(adherence* OR nonadherence* OR persistence*)) OR ((drug OR medication OR patient*) NEXT compliance*):ti,ab,kw OR (possession NEXT

	(rate)):ti,ab,kw<br><br>• **Concept 3 = Chronic Disease**<br>MeSH descriptor: [Chronic Disease] explode all trees OR (Chronic NEXT(disease*)):ti,ab,kw OR MeSH descriptor: [hypertension] explode all trees OR (hypertension*):ti,ab,kw OR MeSH descriptor: [hyperlipidemia] explode all trees OR (hyperlipidemia *):ti,ab,kw OR MeSH descriptor: [Diabetes Mellitus] explode all trees OR (Diabetes NEXT(Mellitus)):ti,ab,kw OR OR MeSH descriptor: [asthma] explode all trees OR (asthma):ti,ab,kw OR MeSH descriptor: [COPD] explode all trees OR (COPD):ti,ab,kw<br><br>• **Concept 4 = Low Middle Income Countries**<br>MeSH descriptor: [Developing Countries] explode all trees OR ((developing OR low-middle-income OR third-world OR emerging) NEXT country*):ti,ab,kw OR ((low OR limited OR constrained OR delimited OR restricted OR restrained) NEXT resource):ti,ab,kw OR “Africa”:ti,ab,kw OR ((Southeastern OR Central OR Western) NEXT Asia):ti,ab,kw OR ((Central OR South) NEXT America):ti,ab,kw OR (“Mongolia” OR “Caribbean” OR “Transcaucasia” OR “Afghan*” OR “Guinea* OR “Peru” OR “Alban*” OR “Philippin*” OR “Algeria*” OR “Haiti*” OR “Romania*” OR “Samoa*” OR “Hondura*” OR “Rwand*” OR “Angola*” OR “Hungar*” OR “Argentin*” OR “India” OR “Sao Tome” OR “Armeni*” OR “Indonesia*” OR “Senegal*” OR “Azerbaijan” OR “Iran*” “Serbia*” OR “Banglad*” OR “Iraq*” OR “Seychell*” OR “Belarus*” OR “Jamaic*” OR “Sierra Leon*” OR “Beliz*” OR “Jordan*” OR “Solomon” OR “Benin*” OR “Kazakhstan*” OR “Somalia*” OR “Bhutan*” OR “Kenya*” OR “South Africa*” OR “Bolivia*” OR “Kiribati” OR “South Sudan*” OR “Bosnia*” OR “Herzegovin” OR “Korea*” OR “Sri Lank*” OR “Bostwan*” OR “Kosovo” OR “St.Lucia” OR “Brazil*” OR “Kyrgyz*” OR “St. Vincent” OR “Grenadin*” OR “Bulgaria*” OR “Burkina Faso” OR “Burund*” OR “Cabo Verde” OR “Cambodia*” OR “Cameroon*” OR “Central Africa*” OR “Chad” OR “China” OR “Colombia*” OR “Comoros*” OR “Congo” OR “Costa Rica” OR “Cote D’ivoire” OR “Cuba” OR “Djibouti” OR “Dominica*” OR “Ecuador*” OR “Egypt*” OR “El Salvador*” OR “Equatorial Guinea” OR “Eritrea” OR “Eswatini” OR “Ethiopia” OR “Fiji” OR “Gabon” OR “Gambia” OR “Ghana” OR “Grenada” OR “Guatemala” OR “Guinea” OR “Guinea-bissau” OR “Guyana” OR “Lao” OR “Lebanon” OR “Lesotho” OR “Liberia” OR “Libya” OR “Madagascar” OR “Malawi” OR “Malaysia” OR “Maldives” OR “Mali” OR “Marshall island*” OR “Mauritan*”

	OR “Mauritiu*” OR “Mexico” OR “Micronesia” OR “Moldova*” OR “Montenegro” OR “Morocco” OR “Mozambique” OR “Myanmar” OR “Namibia” OR “Nepal” OR “Nicaragua” OR “Niger*” OR “North macedonia” OR “Pakistan” OR “Panama” OR “Papua new guinea” OR “Paraguay” OR “Russia*” OR “Suriname” OR “Syrian*” OR “Tajikist*” OR “Tanzania*” OR “Thailand” OR “Timor-leste” OR “Togo” OR “Tonga” OR “Tunisia” OR “Turkey” OR “Turkmenistan” OR “Tuvalu” OR “Uganda” OR “Ukraine” OR “Uzbekistan” OR “Vanuatu” OR “Vietnam” OR “West bank and gaza” OR “Yemen” OR “Zambia” OR “Zimbabwe”):ti,ab,kw

Supplementary 3. Country of Study (total N=181)

Country of study	Number of Studies	Percentage (%)
Ethiopia	27	14,9
India	18	9,9
Nigeria	16	8,8
China	15	8,3
Pakistan	15	8,3
Malaysia	11	6,1
Brazil	10	5,5
Palestine	8	4,4
Ghana	7	3,9
Iran	6	3,3
Cameroon	4	2,2
Jordan	4	2,2
Lebanon	4	2,2
Nepal	4	2,2
Turkey	4	2,2
Egypt	3	1,7
Indonesia	3	1,7
Mexico	3	1,7
Bangladesh	2	1,1
Kenya	2	1,1
Cambodia	1	0,6
Congo	1	0,6
Eritrea	1	0,6
Laos	1	0,6
Peru	1	0,6
South Africa	1	0,6
Sri Lanka	1	0,6
Thailand	1	0,6
Uganda	1	0,6
Uzbekistan	1	0,6
Vietnam	1	0,6
Zambia	1	0,6
Zimbabwe	1	0,6
Lebanon and Jordan	1	0,6
Ghana and Nigeria	1	0,6

Supplementary 4. Study Characteristics

Year	Authors	Country of study	Aim of study	Study design	Study period	Type self-reported instrument	Response rate of self-reported instrument	Population (type of chronic disease)	Sample size	Type of Medication	Adherence phases
2013	Zyoud, S.H	Palestine	to investigate the factors associated with adherence to antihypertensive therapy among hypertensive patients and to assess the relationship between antihypertensive medication adherence and treatment satisfaction	cross-sectional	3 months	MMAS-8	96.4%	hypertension patients	410	antihypertensive	implementation
2014	Sweileh, W.M	Palestine	to assess medication adherence and its potential association with beliefs and diabetes –related knowledge in patients with type II DM	cross-sectional	3 months	MMAS-8	N/A	T2DM patients	405	antidiabetic	implementation
2020	Shakarneh, J.K	Palestine	to evaluate medication adherence in patients with dyslipidaemia in association with patient beliefs about medicines	cross-sectional	6 months	MMAS-4	84.1%	dyslipidemia patients	220	antihyperlipidemic drugs	implementation
2015	Al-Ramahi, R	Palestine	To assess adherence of Palestinian hypertensive patients to therapy and to investigate the effect of a range of demographic and psychosocial variables on medication adherence	cross-sectional	3 months	MMAS-8	N/A	hypertension patients	384	antihypertensive	implementation

2020	Khdour, M.R	Palestine	To examine the mean differences between patient beliefs about medicine with reference to adherence and glycaemic control	cross-sectional	3 months	MMAS-4	N/A	T2DM patients	380	antidiabetic	implementation
2017	Elsous, A.	Palestine	to evaluate the adherence to anti-diabetic medications among patients with type 2 diabetes mellitus (DM)	cross-sectional	3 months	MMAS-4	90%	T2DM patients	372	antidiabetic	implementation
2020	Salama, H.M.	Egypt	to assess the effect of beliefs about medicines on adherence to medications in diabetic patients	cross-sectional	4 months	MMAS-8	N/A	T2DM patients	89	antidiabetic	implementation
2014	Abebe, S.M.	Ethiopia	to assess the magnitude of medication adherence and factors associated with it among adult persons with diabetes in northwest Ethiopia	cross-sectional	2 months	MMAS-8	96.01%	diabetic patients	407	antidiabetic	implementation
2014	Malik, A.	Uzbekistan	to study hypertension-related knowledge, practice and drug adherence of inpatients, and to examine an association between the knowledge regarding hypertension with BP control status and drug adherence.	cross-sectional	4 months	MMAS-4	93%	hypertension patients	226	anti hypertensive	initiation and implementation
2011	Jamous, R.M.	Palestine	to assess hypoglycaemic medication adherence and its association with treatment satisfaction.	cross-sectional	3 months	MMAS-8	99.2%	diabetic patients	130	antidiabetes	implementation

			Setting								
2020	Sarkodie, E.	Ghana	to determine factors that influence adherence to oral antihypertensive drugs among patients attending two district hospitals in the Volta Region of Ghana	cross-sectional	3 months	MMAS-8	N/A	hypertension patients	370	anti hypertensive	implementation
2017	Nazir, S.U.R	Pakistan	to find the relationship between general Health Related Quality of Life (HRQoL) and compliance to the treatment among type 2 diabetes mellitus patients (T2DM) in Sargodha, Pakistan.	cross-sectional	4 months	MMAS-8	N/A	T2DM patients	392	antidiabetic	initiation and implementation
2019	Turan, G.B	Turkey	to determine the effect of social support on drug treatment adherence in patients with hypertension.	cross-sectional	6 months	Medication Adherence Self-Efficacy Scale (MASES)	N/A	hypertension patients	259	anti hypertensive	implementation
2019	Nguyen, T.	Vietnam	to evaluate the impact of a pharmaceutical care program led by pharmacists in the improvement of medication adherence and quality of life for COPD patients in Vietnam.	cross-sectional	12 months	MMAS-8	50%	COPD patients	211	COPD medications	implementation

2021	Cai, Q.	China	to investigate the relationship between illness perceptions, medication beliefs, and self-reported adherence to inhaled corticosteroid (ICS) therapy in adult Chinese patients with asthma	cross-sectional	12 months	Medication Adherence Report Scale for Asthma (MARS-A)	N/A	asthma patients	234	ICS medication	implementation
2017	Erku, D.A	Ethiopia	to evaluate whether a pharmacist-led medication therapy management, compared to the usual care, could enhance medication adherence and reduce hospital admission in patients with type 2 diabetes mellitus	randomized controlled trial	6 months	MMAS-8	84.2%	T2DM patients	127	anti diabetic	implementation
2018	Teshome, D.F.	Ethiopia	to assess the prevalence and associated factors of optimal BP control among hypertensive patients attending at a district hospital.	cross-sectional	3 months	MMAS-4	90%	hypertension patients	416	anti hypertensive	implementation
2019	Pan, J.	China	To assess the adherence level of antihypertensive treatment and identify any associated risk factors in a sample of hypertensive patients from China.	cross-sectional	6 months	therapeutic adherence scale for hypertensive patients (TASHP)	97.6%	hypertension patients	500	anti hypertensive	implementation
2018	Ajayi, D.T	Nigeria	to determine the association between treatment satisfaction and medication adherence among hypertensive patients in Ibadan, Nigeria.	cross-sectional	N/A	MMAS-8	95%	hypertension patients	342	anti hypertensive	implementation

2020	Shakya, R.	Nepal	o identify illness perception and treatment adherence among patients with HTN in a tertiary hospital in Kathmandu, Nepal.	cross-sectional	4 months	Hill-Bone medication adherence scale	95%	hypertension patients	204	anti hypertensive	implementation
2021	Adomako, N.O.	Ghana	to evaluate adherence and accessibility to antihypertensive medications at two different levels of healthcare facilities in Kumasi, Ghana, and determine factors associated with medicine accessibility and adherence.	cross-sectional	N/A	MARS-10	N/A	hypertension patients	485	anti hypertensive	implementation
2021	Pan, J.	China	to examine the effect of social support on the treatment adherence in hypertension in China	cross-sectional	5 months	Hill-Bone medication adherence scale	93%	hypertension patients	487	anti hypertensive	implementation
2019	Aminde, L.N.	Cameroon	to assess the level of adherence and factors influencing non-adherence to antidiabetic medication among patients with type-2 diabetes	cross-sectional	2 months	Medication Compliance Questionnaire (MCQ)	N/A	T2DM patients	195	anti diabetic	implementation
2013	Ahmad, N.S.	Malaysia	to assess adherence to medications and to identify factors that are associated with nonadherence in type 2 diabetes mellitus (T2DM) patients at Primary Health Clinics of the Ministry of Health in Malaysia	cross-sectional	7 months	Medication Compliance Questionnaire (MCQ)	92.8%	T2DM patients	600	anti diabetic	implementation

2015	Akintunde, A.A.	Nigeria	to describe the level of adherence to antihypertensive medications, its determinants and whether any difference exist between those attending specialty clinic or general outpatient department (GOPD) Clinic in a Nigerian University Teaching Hospital. Subjects	cross-sectional	6 months	MMAS-8	N/A	hypertension patients	114	anti hypertensive	implementation
2017	Akoko, B.M.	Cameroon	to assess knowledge of hypertension and to determine factors affecting the compliance of hypertensive patients to their antihypertensive drugs.	cross-sectional	4 months	MMAS-8	100%	hypertension patients	221	anti hypertensive	implementation
2015	Chan, C.W.	Malaysia	to assess the adherence to antihypertensive agents using Morisky Medication Adherence Scale (MMAS-8) among primary care patients, and to determine whether the blood pressure control is associated with the level of adherence.	cross-sectional	3 months	MMAS-8	95.1%	hypertension patients	231	anti hypertensive	implementation
2015	Lulebo	Congo	to measure non-adherence to antihypertensive medication and to identify its predictors	cross-sectional	2 months	MMAS-4	96.8%	hypertension patients	408	anti hypertensive	implementation

2015	Abbas	Pakistan	To report medication adherence among ambulatory patients with diabetes mellitus (DM) using Morisky 8-item medication adherence MMAS-8 scale	cross-sectional	3 months	MMAS-8	85.6%	diabetic patients	300	anti diabetic	implementation
2014	Arulmozhi, S.	India	To assess medication adherence and adherence to self care among type 2 diabetics who were admitted to a tertiary care hospital	cross-sectional	2 months	MMAS-8	N/A	T2DM patients	150	anti diabetic	implementation
2012	Ambaw	Ethiopia	to assess adherence to antihypertensive therapy and associated factors among HTN patients on follow up at University of Gondar Referral Hospital	cross-sectional	5 months	MMAS-4	100%	hypertension patients	384	anti hypertensive	implementation
2018	Waari, G.	Kenya	to assess medication adherence among Type 2 diabetes mellitus patients	cross-sectional	3 months	MMAS-8	N/A	T2DM patients	289	anti diabetic	implementation
2021	Ozoh, O.B	Nigeria	to assess the feasibility of a nurse-led asthma education program and its effect on asthma knowledge and outcomes in Lagos, Nigeria	Quasi experemintal	6 months	MMAS-8	52.5%	asthma patients	80	asthma controller medication	implementation

2020	Tefera, Y.G.	Ethiopia	to assess the diabetic health literacy level and its association with gly-cemic control among adult patients with type 2 diabetes mellitus attending the outpatient clinic of University of Gondar Comprehensive Specialized Hospital (UOGCSH): Northwest Ethiopia	cross-sectional	1 month	MMAS-4	100%	T2DM patients	400	anti diabetic	implementation
2019	Yuvaraj, K.	India	To determine the prevalence of drug adherence among patients with NCD in rural Puducherry	cross-sectional	2 months	MMAS-4	93.8%	diabetic, hypertension, asthma patients	277	anti diabetic, anti hypertensive, asthma	implementation
2020	Pirasath, S.	Sri Lanka	to assess the patient's knowledge and awareness about hypertension and adherence to anti-hypertensive medication among hypertensive patients	cross-sectional	6 months	MMAS-7	95%	hypertension patients	384	anti hypertensive	implementation
2018	Abba, Z.I.	Cameroon	to determine the prevalence and risk factors of cognitive dysfunction in patients with type 2 diabetes and to assess its influence on medication adherence.	cross-sectional	4 months	MMAS-8	99,10%	T2DM patients	223	anti diabetic	implementation

2016	Dego, T.R.	Ethiopia	to assess adherence to anti-hypertensive medication and contributing factors among non-comorbid hypertensive patients in two hospitals of Jimma town, South West Ethiopia	cross-sectional	2 months	MMAS-4	100%	hypertension patients	120	anti hypertensive	implementation
2021	Shimels, T.	Ethiopia	to assess the magnitude and associated factors of poor medication adherence among diabetic and hypertensive patients visiting public health facilities in Addis Ababa, Ethiopia during the COVID-19 pandemic	cross-sectional	1 month	MMAS-8	90%	T2DM and hypertension patients	422	antidiabetic and antihypertensive medications	implementation
2018	Balasubramanian, A.	India	assessing the level of adherence to medication and to identify factors associated with it in people with hypertension in a rural population of Kerala	cross-sectional	1 month	MMAS-4	50.8%	hypertension patients	189	anti hypertensive	implementation
2020	Mahmood	Pakistan	to assess the prevalence and predictors of non-adherence to antihypertensive medication among patients with hypertension attending various healthcare settings in Islamabad, Pakistan.	cross-sectional	4 months	MMAS-8	95%	hypertension patients	741	anti hypertensive	implementation

2021	Chaudhary, A.P.	India	to determine medication compliance among hypertension patients and discover factors influencing medication compliance.	cross-sectional	5 months	Hill-Bone medication adherence scale	91.8%	hypertension patients	90	anti hypertensive	implementation
2020	Mathur, D.	India	to assess the awareness of hypertension, medication adherence, and dietary pattern in hypertensive population of western Rajasthan. M	cross-sectional	4 months	MMAS-8	100%	hypertension patients	383	anti hypertensive	implementation
2016	Kassahun, A.	Ethiopia	To assess nonadherence and factors affecting adherence of diabetic patients to anti-diabetic medication in Assela General Hospital (AGH), Oromia Region, Ethiopia	Cross sectional	2 months	MMAS-4	95%	T2DM patients	285	Antidiabetic	implementation
2019	Okeke, H.C.	Nigeria	to determine the relationship between patients' socio-demographic characteristics and adherence to antihypertensive therapy in the Southern Senatorial District of Cross River State	Cross sectional	N/A	MMAS-8	84.2%	hypertension patients	500	anti hypertensive	implementation
2020	Afaya, R.A.	Ghana	to evaluate medication adherence, self-care behaviours and diabetes knowledge among patients with type 2 diabetes mellitus in Ghana	Cross sectional	3 months	Medication Adherence Questionnaire (MAQ)	91.7%	T2DM patients	360	Antidiabetic	implementation

2017	Iloh, G.U.P	Nigeria	to determine the role of treatment satisfaction in medication adherence, and BP control among adult Nigerians with essential hypertension	Cross sectional	8 months	no name	N/A	hypertension patients	140	anti hypertensive	implementation
2021	Takahashi, E.	Laos	to examine the rate of medication adherence to antihypertensive medicines among outpatients with hypertension in rural districts of the Savannakhet.	Cross sectional	7 months	MMAS-4	63%	hypertension patients	75	anti hypertensive	implementation
2019	Amer, M.	Pakistan	To determine knowledge regarding hypertension, adherence to medication and Health- Related Quality of Life (HRQoL), and their associations in hypertensive patients in Pakistan.	Cross sectional	N/A	MMAS-8	N/A	hypertension patients	384	anti hypertensive	implementation
2019	Nonogaki, A.	Cambodia	to identify factors associated with dia- betes medication adherence among people with diabetes mellitus in poor urban areas of Phnom Penh, Cambodia	Cross sectional	3 months	MMAS-4	90.6%	diabetic patients	773	anti diabetic	implementation
2021	Raja, W.	India	to estimate adherence to antihypertensive therapy and its determinants among OPD patients attending two primary care hospitals in Kashmir valley.	Cross sectional	3 months	Hill-Bone medication adherence scale	90.6%	hypertension patients	406	anti hypertensive	implementation

2017	Ali, M.	Ethiopia	to assess adherence to anti-diabetic medications and associated factors among patient with diabetes mellitus receiving care at Zewditu Memorial Hospital	Cross sectional	2 months	MMAS-8	N/A	T2DM patients	146	anti diabetic	implementation
2019	Ullah, M.	Bangladesh	to find the causes of non adherence of drugs among the patients with HTN in a secondary care hospital of Bangladesh	Cross sectional	4 months	Hill-Bone medication adherence scale	N/A	hypertension patients	144	anti hypertensive	implementation
2020	G/Tsadik, D.	Ethiopia	to assess adherence to antihypertensive treatment and factors associated with it in Central Ethiopia.	Cross sectional	9 months	MMAS-8	96%	hypertension patients	989	anti hypertensive	implementation
2020	Roka, T.	Nepal	to measure adherence to anti-hypertensive medication and factors associated with low adherence.	Cross sectional	2 months	MMAS-4	N/A	hypertension patients	216	anti hypertensive	implementation
2016	Bakar, Z.A	Malaysia	To determine the satisfaction and current adherence status of diabetes mellitus patients at the diabetes MTAC (DMTAC) and its between patient satisfaction and adherence.	Cross sectional	3 months	MMAS-8	N/A	T2DM patients	165	anti diabetic	implementation
2016	Yang, S.	China	to determine the factors associated with antihypertensive adherence based on the Health Belief Model (HBM)	Cross sectional	3 months	MMAS-4	N/A	hypertension patients	745	anti hypertensive	implementation

2017	Berhe, D.F.	Ethiopia	to evaluate the impact of adverse drug events (ADEs) and treatment satisfaction on antihypertensive medication adherence.	Cross sectional	7 months	MMAS-8	98.4%	hypertension patients	984	anti hypertensive	implementation
2020	Sefah, I.A.	Ghana	to assess adherence to oral hypoglycemic drugs and factors that affect adherence among patients with T2DM in the Volta Region of Ghana	cross-sectional	2 months	MMAS-8	90%	T2DM patients	400	antidiabetic	implementation
2020	Saraiva, E.M.S	Brazil	to evaluate other factors for medication non-adherence by studying patients with full access to oral hypoglycemic agents	cross-sectional	12 months	MMAS-4	91.7%	T2DM patients	300	antidiabetic	implementation
2018	Bermeo-Cabrera, J.	Mexico	To investigate factors associated with insulin adherence in subjects with type 2 diabetes mellitus (T2D) attending a tertiary care centre in Mexico City.	cross-sectional	9 months	MMAS-8	N/A	T2DM patients	200	insulin	implementation
2009	Lerman, I	Mexico	to evaluate the psy-chosocial barriers to insulin use in low-income, type 2 diabetic patients, the clinical characteristics of these patients, and the possible causes of nonadherence to insulin regimens months after prescription.	cohort	9 months	Self Care Inventory Diabetes	N/A	T2DM patients	29	insulin	implementation

2019	Abate, T.W.	Ethiopia	to assess medication non-adherence and associated factors among adult diabetes in Felege Hiwot Referral Hospital Bahir Dar city administration	cross-sectional	2 months	MMAS-8	99%	T2DM patients	416	antidiabetic	implementation
2019	Oluwole, E.O	Nigeria	to determine the association between medication adherence and treatment satisfaction among hypertensive patients attending hypertension outpatient clinic in Lagos University Teaching Hospital (LUTH), Nigeria	cross-sectional	3 months	MMAS-8	N/A	hypertension patients	500	anti hypertensive	implementation
2019	Mariye, T.	Ethiopia	to assess the level of adherence to insulin therapy and associated factors among diabetes mellitus patients.	cross-sectional	3 months	MMAS-8	100%	T2DM patients	273	insulin	implementation
2015	Jackson, I.	Nigeria	to assess medication adherence among type 2 diabetes patients and to identify patient characteristics and probable factors associated with nonadherence	cross-sectional	9 months	MMAS-8	84.2%	T2DM patients	360	antidiabetic	implementation
2018	Basharat, S.	Pakistan	To assess the degree of medication adherence among asthma patients and association of asthma control level with the degree of adherence.	cross-sectional	12 months	MMAS-8	N/A	asthma patients	310	asthma controller medication	implementation

2015	Fadare, J.	Nigeria	to assess the level of adherence to antidiabetic drugs among outpatients in a teaching	cross-sectional	3 months	MMAS-8	N/A	T2DM patients	129	antidiabetic	implementation
2021	Al-Qerem, W.	Jordan	to assess medication adherence and explore its predictors in outpatients with type 2 diabetes.	cross-sectional	12 months	MMAS-4	79.7%	T2DM patients	287	antidiabetic	implementation
2017	Iqbal, Q.	Pakistan	to assess medication adherence among type 2 diabetic patients in Quetta city, Pakistan. Methods:	cross-sectional	6 months	Drug Attitude Inventory (DAI)	90.9%	T2DM patients	300	antidiabetic	implementation
2021	Akram, F.	Pakistan	To explore relationship involving Ego Defence Mechanism, Medication Adherence and Self-Management of patients with type 2 diabetes	cross-sectional	12 months	MMAS-8	N/A	T2DM patients	150	antidiabetic	implementation
2020	Ayodapo, A.O.	Nigeria	to determine the role of patient education in medication adherence among hypertensives attending tertiary hospitals in Ekiti State, South Western, Nigeria	cross-sectional	N/A	MMAS-4	N/A	hypertension patients	420	anti hypertensive	implementation
2007	Hashmi	Pakistan	to measure adherence to antihypertensive therapy in a representative sample of the hypertensive Pakistani population	cross-sectional	9 months	MMAS-4	95.2%	hypertension patients	438	anti hypertensive	implementation
2020	Asilar, H.	Turkey	to investigate the effect of loneliness and perceived social support on medication adherence self-efficacy in hypertensive patients.	cross-sectional	4 months	Medication Adherence Self-Efficacy Scale Short Form	N/A	hypertension patients	397	anti hypertensive	implementation

						(MASES-SF)					
2016	Kassahun, T.	Ethiopia	to assess the status of glycemic control and its contributing factors among adult patients with type 2 diabetes mellitus	cross-sectional	2 months	MMAS-8	67.3%	T2DM patients	325	antidiabetic	implementation
2021	Ishaq, R.	Pakistan	to evaluate the correlations between diabetes-related knowledge, medication adherence, and HRQoL among T2DM patients in Pakistan.	cross-sectional	2 months	Drug Attitude Inventory (DAI)	N/A	T2DM patients	300	antidiabetic	implementation
2016	Ngahane, B.H.M.	Cameroon	To determine the adherence rate and identify the predictors of low adherence to asthma controller therapy.	cross-sectional	7 months	MMAS-8	82.7%	asthma patients	201	asthma controller medication	implementation
2019	Adeoye, A.M	Nigeria	to report the pattern and correlate of medication adherence among the uncontrolled hypertensive population	cross-sectional	12 months	MMAS-4	N/A	hypertension patients	148	anti hypertensive	implementation
2021	Andualem, A.	Ethiopia	to assess adherence to antihypertensive medications among adult hypertensive patients in Dessie Referral Hospital.	cross-sectional	2 months	MMAS-4	97.8%	hypertension patients	366	anti hypertensive	implementation

2016	Hossain	Bangladesh	to investigate whether there is an association between sleep duration and treatment compliance among skilled professionals who are experiencing hypertension	cross-sectional	2 months	self-reported compliance test (SC)	82.1	hypertension patients	101	anti hypertensive	implementation
2018	Asgedom, S.W	Ethiopia	to assess antihypertensive medication adherence and associated factors among adult hypertensive patients. A	cross-sectional	2 months	MMAS-8	92%	hypertension patients	280	anti hypertensive	implementation
2019	Jannoo, Z.	Malaysia	To evaluate medication adherence and self-care behaviors among patients with T2DM	cross-sectional	N/A	MMAS-8	66.2%	T2DM patients	497	anti diabetic	implementation
2016	Mohammad, Y.	Lebanon	to evaluate treatment adherence to antihypertensive therapy in Lebanese hyperten-sive patients by estimating the proportion of adherent hypertensive patients using a validated tool and investigates what factors predict this behavior	cross-sectional	N/A	MMAS-8	100%	hypertension patients	210	anti hypertensive	implementation
1995	Garay-Sevilla, M.	Mexico	to investigate factors associated with adherence to diet and medication in non-insulin-dependent diabetes mellitus patients.	cross-sectional	N/A	no name	N/A	diabetic patients	200	anti diabetic	implementation

2015	Bruce, S.P.	Ghana	to examine the level of adherence to oral antidiabetic drugs among patients who visited the teaching hospital and explored the probable contributory factors to non-adherence.	cross-sectional	2 months	MMAS-4	89.3%	T2DM patients	200	anti diabetic	implementation
2012	Karakurt	Turkey	to evaluate concordance with medication and those factors that affect the use of medicines in patients with hypertension.	cross-sectional	4 months	no name	N/A	hypertension patients	750	anti hypertensive	implementation
2016	Alhaddad, I.A.	Lebanon and Jordan	to assess the adherence level to antihypertensive treatment and to identify its associated factors in a sample of hypertensive patients in Lebanon and Jordan.	cohort	5 months	MMAS-4	97.6%	hypertension patients	1470	anti hypertensive	implementation
2014	Kretchy, I.A.	Ghana	to examine the association between locus orientation and adherence to hypertensive medication among adult patients	cross-sectional	3 months	MMAS-8	N/A	hypertension patients	400	anti hypertensive	implementation
2017	Mekonnen, H.S	Ethiopia	to assess adherence level and its determinants for antihypertensive medications among adult hypertensive patients attending the chronic illness clinics of the referral hospitals in northwest Ethiopia.	cross-sectional	2 months	MMAS-8	100%	hypertension patients	409	anti hypertensive	implementation

2021	Mwangasha	Kenya	to determine and explore the interplay between patient knowledge, medication adherence and glycemic control among adult diabetics in a low resource setting.	cross-sectional	N/A	MMAS-8	N/A	diabetic patients	270	anti diabetic	implementation
2019	Balasubramaniam, S.	Malaysia	to explore the association between illness perception and medication adherence, glycemic control, and CKD in an Asian population with the aim of gaining an insight into the importance of illness perception-based psycho- logical management in diabetes	cross-sectional	5 months	MMAS-8	97.4%	T2DM patients	384	anti diabetic	implementation
2015	Vancini-Campanharo	Brazil	to identify the epidemiological profile of hypertension patients, how much they understand about the disease and the rate of adherence to treatment by these patients who had been hospitalized in the Brazilian emergency service.	cross-sectional	4 months	MMAS-4 and Brief Medication Questionnaire (BMQ)	N/A	hypertension patients	116	anti hypertensive	implementation
2006	Hassan, N.B	Malaysia	to identify psychosocial predictors of medication noncompliance in hyper-tensive patients using valid and reliable questionnaires	cross-sectional	6 months	no name	98%	hypertension patients	242	anti hypertensive	implementation

2016	Nazir, S.U.R	Pakistan	The purpose of this study was to investigate the association of diabetes-related knowledge and treatment adherence with glycaemic control among patients with type 2 diabetes mellitus (T2DM) in Pakistan.	cross-sectional	4 months	MMAS-8	N/A	T2DM patients	392	anti diabetic	implementation
2021	Khadka	Nepal	to explore the adherence towards prescribed antihypertensive treatment, and identify factors influencing non-adherence.	cross-sectional	4 months	MMAS-4	N/A	hypertension patients	384	anti hypertensive	implementation
2020	Kretchy, I.A.	Ghana	to estimate distress associated with T2DM and to examine its association with medication adherence.	cross-sectional	3 months	MARS-5	N/A	T2DM patients	188	anti diabetic	implementation
2020	Sartori, A.C.	Brazil	to examine the effect of an educational intervention using WhatsApp? messaging on medication adherence of patients with hypertension and diabetes.	randomized controlled trial	4 months	MMAS-4	81.25%	DM and hypertension patients	403	antidiabetic and antihypertensive medications	implementation
2020	Owolabi	South Africa	to assess the effect of unidirectional text messaging on adherence to dietary and activity regimens among adults living with diabetes in a rural setting of Eastern Cape, South Africa	randomized controlled trial	10 months	no name	90.7%	T2DM patients	216	anti diabetic	implementation

2021	Alfian, S.D	Indonesia	To assess the effects of a targeted and tailored pharmacist-led intervention among patients with type 2 diabetes (T2DM) who are nonadherent to antihypertensive drugs.	randomized controlled trial	3 months	MARS-5	80.4% for control and 77.2% for intervention patients	T2DM patients	41 for control and intervention group	antihypertensive	implementation
2015	Cani, C.G.	Brazil	To evaluate the impact of a clinical pharmacy program on health outcomes in patients with type 2 diabetes undergoing insulin therapy at a teaching hospital in Brazil.	randomized controlled trial	6 months	MMAS-4 and Medication Adherence Questionnaire (MAQ)	89.7%	T2DM patients	78	insulin therapy	implementation
2021	Khudair	Jordan	to assess the level of medication adherence and associated factors among Syrian refugees with hypertension in Jordan.	Cross sectional	2 months	Adherence to Refills and Medications Scale (ARMS)	N/A	hypertension patients	180	anti hypertensive	implementation
2014	Chung, W.W.	Malaysia	To assess the effects of a pharmaceutical care model on medication adherence and glycemic levels of people with type 2 diabetes mellitus	randomized controlled trial	12 months	Malaysian Medication Adherence Scale (MALMAS)	N/A	T2DM patients	241	anti diabetic	implementation
2019	Shen, Y.	China	to evaluate the effectiveness of combining low-cost reminder package and health education to improve medication adherence among hypertensive patients.	randomized controlled trial	8 months	no name	86.9%	hypertension patients	518	anti hypertensive	implementation

2020	Delavar, F.	Iran	To evaluate the effects of self-management education tailored to health literacy on medication adherence and blood pressure control.	randomized controlled trial	3 months	MMAS-8	94.9%	hypertension patients	118	anti hypertensive	implementation
2021	Barikani, A.	Iran	d to determine the impact of motivational interview on self-efficacy, beliefs about medicines and medication adherence among adolescents with asthma.	randomized controlled trial	13 months	MARS-10	88.4%	asthma patients	52	corticosteroid (controller medication)	implementation
2019	Sheilini, M.	India	to investigate the effects of multimodal interventions on medication nonadherence, quality of life (QoL), hypertension (HTN), self-efficacy, and clinical outcome in terms of blood pressure (BP) among elderly people with HTN.	randomized controlled trial	6 months	MMAS-8	77.5%	hypertension patients	160	anti hypertensive	implementation
2020	Nazir, S.U.R	Pakistan	to evaluate the effects of an educational intervention in a pharmacist led, medication management program (MTM) tailored for patients with type 2 diabetes (T2DM).	randomized controlled trial	3 months	MMAS-8	86.9%	T2DM patients	392	anti hypertensive	implementation

2010	Nesari, M.	Iran	To determine whether a nurse telephone follow-up service could improve the level of adherence to a diabetes therapeutic regimen for patients with type 2 diabetes.	randomized controlled trial	3 months	no name	81.3%	T2DM patients	61	anti hypertensive	implementation
2020	Bijam	Iran	to compare the effectiveness of multimedia and traditional methods of patient education in persuading patients with hypertension to stick to their treatment regimens.	quasi experimental	5 months	no name	N/A	hypertension patients	160	anti hypertensive	implementation
2015	Chow, E.P.	Malaysia	to evaluate whether a home-based intervention can result in better understanding about type 2 diabetes mellitus and can increase adherence to prescribed medications	randomized controlled trial	6 months	MMAS-8	80%	T2DM patients	150	anti diabetic	implementation
2018	Supachaipachnipong, S.	Thailand	to evaluate whether a medication education intervention integrated into the routine service of a diabetes clinic and provided by physicians, and nurses could improve the knowledge of medication use, medication beliefs, and medication adherence as well as blood glucose levels among patients with uncontrolled diabetes	quasi experimental	5 months	MMAS-8	88.3%	T2DM patients	86	anti diabetic	implementation

2019	Shi	China	to determine the association between medication literacy and medication adherence in hypertensive patients	Cross sectional	6 months	MMAS-8	93.3%	hypertension patients	320	anti hypertensive	implementation
2015	Ashur, S.T.	Lebanon	to examine how Libyan diabetes patients perceive diabetes and how their perceptions influence their medication adherence	Cross sectional	3 months	MMAS-8	80.7%	T2DM patients	523	anti diabetic	implementation
2013	Mukora-Mutseyekwa, F.N.	Zimbabwe	to estimate prevalence of drug adherence behavior, and establishing the association between drug adherence behavior and achievement of BP control	Cross sectional	N/A	MMAS-4	N/A	hypertension patients	102	anti hypertensive	implementation
2016	Hou, Y.	China	to identify the association between aging perceptions and antihypertensive drug adherence among Chinese older adults.	cross sectional	10 months	MMAS-8	96.7%	hypertension patients	585	anti hypertensive	implementation
2021	Mebrahtu, G.	Eritrea	to evaluate the frequency of AHM and associated risk factors in patients attending a national referral hospital in Asmara, Eritrea. Methods:	Cross sectional	5 months	MARS-10	98.2%	hypertension patients	335	anti hypertensive	implementation
2014	Wang, W.	China	To investigate the factors that influence medication adherence in Chinese community-dwelling older adults with	Cross sectional	6 months	MMAS-4	N/A	hypertension patients	382	anti hypertensive	implementation

			hypertension.								
2021	Mohamad	Lebanon	to determine the self-reported medication adherence prevalence and its predictors and exploring reasons for low adherence among these patients.	mixed method study	1 month	MMAS-8	87.6	hypertension patients	361	anti hypertensive	implementation
2017	Wei.L	China	To investigate beliefs about medicines and their association with medicine adherence in patients with chronic diseases	cross-sectional	4 months	MARS-5	N/A	T2DM patients	315	antidiabetic	implementation
2017	Al-Daken	Jordan	To assess Jordanian hypertensive patients' adherence rate to hypertension therapeutic regimen (HTR) and to identify the strongest predictors of adherence rate among such patients	Cross sectional	N/A	Hill-Bone medication adherence scale	N/A	hypertension patients	192	anti hypertensive	implementation
	Gomes	Brazil	Determine the relationship between self-reported adherence to insulin therapeutic regimens in Brazilian patients with type 1 diabetes and demographic, clinical data, glycemic control and cardiovascular risk factors. Methods:	Cross sectional	3 years	MMAS-4	N/A	T1DM patients	1698	insulin	implementation

2017	Gu	China	to examine the relationship between social support and medication adherence in patients with T2DM.	Cross sectional	6 months	MMAS-8	N/A	T2DM patients	331	anti diabetic	implementation
2019	Jarab	Jordan	to investigate the factors that are significantly associated with self-reported medication non-adherence in patients with COPD	Cross sectional	5 months	MMAS-4	70.7%	COPD patients	133	COPD medications	implementation
2017	Samu	India	to explore the relationship between non-adherence with medication and diabetic peripheral neuropathy in patients with type 2 diabetes mellitus (DM) in a private hospital located in South	Cross sectional	12 months	MMAS-8	71.6%	T2DM patients	86	anti diabetic	implementation
2015	Saleem	Pakistan	to evaluate whether a pharmaceutical care intervention can result in better understanding about hypertension, increase medication adherence to antihypertensive therapy and improve overall health-related quality of life.	randomized controlled trial	9 months	Drug Attitude Inventory (DAI)	97.5%	hypertension patients	385	anti hypertensive	implementation
2018	Abdulsalim	India	to evaluate the effectiveness of a clinical pharmacist-led intervention on medication adherence in COPD patients in a teaching hospital	randomized controlled trial	2 years	Medication Adherence Questionnaire (MAQ)	79.2%	COPD patients	260	COPD medications	implementation

2020	Alfian, S.D	Indonesia	To identify factors associated with non-adherence as well as subtypes of non-adherence to anti-hypertensive or antihyperlipidemic drugs among patients with type 2 diabetes in Indonesia.	cross-sectional	6 months	MARS-5	97.1%	T2DM patients	571	antihypertensive and/or antihyperlipidemic drugs	implementation
2018	Akrom, A.	Indonesia	to evaluate the level of adherence and quality of life of diabetic patients with hypertension in Bantul Public Hospital (BPH), Bantul, Indonesia.	cross-sectional	13 months	MMAS-8	N/A	T2DM patients	143	antidiabetic and/or antihypertensive	initiation or implementation
2011	Al-Qazaaz, H.K.	Malaysia	to investigate any association of knowledge and medication adherence with glycemic control in patients with type 2 diabetes mellitus	cross-sectional	6 months	MMAS-8	N/A	T2DM patients	540	antidiabetic	implementation
2016	Bizu, G.	Ethiopia	to assess adherence to anti- diabetic medications and determinant factors including medication related beliefs among type 2 diabetic patients attending their treatment in primary healthcare centers	cross-sectional	2 months	MMAS-8	N/A	T2DM patients	155	antidiabetic	implementation
2013	Teklay, G.	Ethiopia	to assess the pattern of non-adherence to diabetic therapy and associated factors among type 2 diabetic patients	cross-sectional	3 months	MMAS-4	N/A	T2DM patients	267	antidiabetic	implementation

2014	Ali, M. A	Ethiopia	to investigate antihypertensive medication non-adherence and its determinants among patients on follow up	cross-sectional	2 months	MMAS-8	N/A	hypertension patients	121	antihypertensive	implementation
2012	Ramli, A.	Malaysia	to assess adherence to medications in patients undergoing hypertensive treatment in the Primary Health Clinics of	cross-sectional	7 months	Medication Compliance Questionnaire (MCQ)	N/A	hypertension patients	600	antihypertensive	implementation
2010	Ungari, A.Q.	Brazil	to analyze adherence to drug treatment in hypertensive patients enrolled	cross-sectional	4 months	MMAS-4	N/A	hypertension patients	109	antihypertensive	implementation
2012	Saleem, F.	Pakistan	to evaluate the association between medication adherence and HRQoL.	cross-sectional	2 months	Drug Attitude Inventory	N/A	hypertension patients	385	antihypertensive	implementation
2011	Saleem, F.	Pakistan	To evaluate the association between patient's knowledge of hypertension management and medication adherence. Methods:	cross-sectional	2 months	Drug Attitude Inventory	N/A	hypertension patients	385	antihypertensive	implementation
2020	Nandini, H.C	India	to measure adherence to antidiabetic medication and factors contributing to it	cross-sectional	3 months	MARS-5	N/A	T2DM patients	250	antidiabetic	implementation
2020	Demoz, G.T	Ethiopia	to investigate the level of medication adherence to antidiabetic therapy and to identify possible predictors of poor adherence	cross-sectional	2 months	Brief Medication Questionnaire (BMQ)	92.9%	T2DM patients	384	antidiabetic	implementation

2017	Kavitha, S	India	to evaluate the treatment adherence and factors affecting non adherence among Type 2 diabetes mellitus patients	cross-sectional	2 months	MMAS-8	N/A	T2DM patients	150	antidiabetic	implementation
2016	Abebaw, M.	Ethiopia	to assess adherence and associated factors towards antidiabetic medication among type II diabetic patients in University of Gondar Hospital, Diabetic Clinic, Gondar, North-west Ethiopia	cross-sectional	4 months	MMAS-4	90%	T2DM patients	288	antidiabetic	implementation
2010	Adisa, R.	Nigeria	To assess adherence to medication among ambulatory patients with type 2 diabetes, ascertain the level of glycemic control, and evaluate patients' opinions on probable reasons for non-adherence with a view to identify areas of intervention to improve adherence.	Cross sectional	2 months	MMAS-4	81.4%	T2DM patients	140	Antidiabetic	implementation
2016	Ogheonovo, A.	Nigeria	to determine the level of adherence with medications by respondents and to ascertain the reasons for non-adherence.	Cross sectional	3 months	MMAs-8	N/A	Diabetic patients	300	antidiabetic	implementation

201 5	Divya, S.	India	to determine the non-adherence to medication and to assess the factors affecting the same among the Type 2 diabetes mellitus (DM) patients in a tertiary care hospital in South India.	Cross sectional	4 month s	MMAS-8	N/A	T2DM patients	150	antidiabetic	implementatio n
201 4	Gelaw, B.K	Ethiopia	to determine the magnitude of nonadherence and its contributing factors among diabetic patients attending the diabetic clinic in Adama Hospital	Cross sectional	2 month s	No name	98.3%	Diabetic patients	270	antidiabetic	implementatio n
200 9	Gimene s, H.T	Brazil	to assess patient adherence to antidiabetic drug therapy and its association with factors related to the patient, patient-provider relationship, therapeutic regimen and the disease itself	Cross sectional	N/A	Measure Treatment Adherence (MTA)	N/A	Diabetic patients	46	antidiabetic	implementatio n
201 5	Heissa m, K.	Egypt	to assess patterns and obstacles to adherence of type 2 diabetic patients to their oral hypoglycemic drugs.	Cross sectional	2 month s	Measure Treatment Adherence (MTA)	N/A	T2DM patients	372	antidiabetic	initiation and implementatio n
201 5	Istili, P.T.	Brazil	to assess medication adherence to oral glucose-lowering agents	Cross sectional	6 month s	Brief Medication Questionnai re (BMQ)	N/A	T2DM patients	60	antidiabetic	initiation and implementatio n

2017	Jemal, A.	Ethiopia	to assess adherence to oral antidiabetic drugs among diabetic patients attending chronic ambulatory wards	Cross sectional	2 months	MMAS-4	98%	T2DM patients	200	antidiabetic	implementation
2014	Omar, M.S.	Malaysia	to verify the knowledge level and medication adherence level among type 2 diabetes mellitus geriatric in-patients.	Cross sectional	6 months	MMAS-4	N/A	Diabetic patients	147	antidiabetic	initiation and implementation
2012	Iloh, G.U.P	Nigeria	to determine the blood glucose control and medication adherence among adult type 2 diabetic Nigerians attending a primary care clinic in under-resourced environment of Eastern Nigeria	Cross sectional	9 months	Measure Treatment Adherence (MTA)	N/A	T2DM patients	120	antidiabetic	implementation
2014	Sajith, M.	India	to evaluate self-reported medication adherence and to identify factors linked with poor adherence in patients with type 2 diabetes mellitus.	Cross sectional	6 months	MMAS-4	N/A	Diabetic patients	105	antidiabetic	initiation and implementation
2015	Sankar, U.V	India	to determine medications adherence and factors associated with poor adherence in community-dwelling adults with diabetes in southern India	Cross sectional	3 months	MMAS-8	96.1%	Diabetic patients	360	antidiabetic	initiation and implementation

2010	Shams, M.E.E.	Egypt	to examine the rate of medication adherence and different factors affecting it among Type 2 diabetic patients in Egypt.	Cross sectional	5 months	Measure Treatment Adherence (MTA)	54.2%	T2DM patients	417	antidiabetic	implementation
2016	Tsehay, T.	Ethiopia	to assess the rate of antidiabetic medication adherence and factors associated with poor medication adherence together with reasons for non-adherence among ambulatory type 2 diabetic patients in Tikur Anbessa Specialized Hospital (TASH), Ethiopia	Cross sectional	2 months	MMAS-4	N/A	T2DM patients	322	antidiabetic	implementation
2018	MR, Almadhoun	Palestine	To assess medication adherence and its association with glycemic control among T2DM patients.	Cross sectional	4 months	MMAS-8	91.9%	T2DM patients	148	antidiabetic	implementation
2013	Faria, H.T.G	Brazil	To Investigate the association between adherence to treatment of type 2 diabetes mellitus and socio-demographic, clinical and metabolic control variables.	Cross sectional	10 months	Measure Treatment Adherence (MTA)	84.4%	T2DM patients	423	Antidiabetic	implementation
2012	Okoro, R.N.	Nigeria	to assess the patient's antihypertensive medication adherence level in non-comorbid hypertension and to identify and evaluate the causes of medication non-adherence	Cross sectional	4 months	no name	N/A	hypertension patients	152	antihypertensive	implementation

2019	Olorunfemi, O.	Nigeria	to determine the level of medication adherence, patient medication belief and to determine the correlation between medication belief and medication adherence among patients with diabetes	Cross sectional	N/A	MMAS-4	N/A	T2DM patients	180	Antidiabetic	implementation
2017	Son, N.E.	Turkey	to investigate the relationship between metabolic control and Morisky scale in the population of diabetic patients and the factors that contribute to low motivation and knowledge	Cross sectional	5 months	MMAS-6	N/A	T2DM patients	351	Antidiabetic	implementation
2016	Wu, P.	China	to identify patients' beliefs about taking oral antidiabetic drugs as prescribed and measure the correlations between beliefs and medication adherence.	Cross sectional	N/A	MMAS-8	N/A	T2DM patients	130	Antidiabetic	implementation
2011	Aflakseir, A.	Iran	to examine the role of illness and medication perceptions on medication adherence in a group of Iranian patients with type 2 diabetes.	Cross sectional	N/A	MARS-10	91%	T2DM patients	112	Antidiabetic	implementation
2010	Atulomah, N.O	Nigeria	to collect information about perceived severity and threat to life from poor treatment response and medication adherence in hypertensives	Cross sectional	N/A	no name	N/A	hypertension patients	103	anti hypertensive	initiation and implementation

2022	Faisal, K.	Uganda	To assess the factors contributing to non-adherence to antidiabetic medication among diabetes mellitus patients in the Diabetic clinic at Mbarara Regional Referral Hospital	cross sectional	4 months	Hill-Bone medication adherence scale	100%	Diabetic patients	257	antidiabetic	implementation
2016	Inbaraj, L.R	India	to estimate prevalence of non-adherence and identify perceptions and practices associated with non-adherence.	cross sectional	3 months	MMAS-4	N/A	Diabetic patients	100	antidiabetic	implementation
2015	Wong, M.C.S	China	to evaluate the association between glycaemic control and medication adherence among Chinese diabetes patients in the clinical setting.	cross sectional	N/A	MMAS-8	80.7%	T2DM patients	565	antidiabetic	implementation
2019	Kusa, W.	Ethiopia	to determine the magnitude of medication non-adherence and its associated factors among type II Diabetes Mellitus patients in Adama hospital medical college, central Ethiopia.	cross sectional	2 months	MMAS-8	90%	T2DM patients	140	antidiabetic	implementation
2019	Bhattarai, B.	India	to identify the Non adherence Patient to the Diabetic treatment, and to assess the contributing factors for non adherence	cross sectional	2 months	MMAS-4	N/A	T2DM patients	214	antidiabetic	implementation
2015	Boima, V.	Ghana and Nigeria	to determine factors associated with medication non-adherence among hypertensives in Ghana and Nigeria.	multicenter cross sectional	6 months	MMAS-8	N/A	hypertension patients	357	anti hypertensive	implementation

2015	Sontake, S.	India	To evaluate adherence to therapy and study factors associated with non-adherence in patients of type 2 diabetes mellitus (DM)	cross sectional	N/A	MMAS-8	N/A	T2DM patients	150	antidiabetic	implementation
2020	Cai, Q.	China	to assess the association between patients’ belief and adherence to inhaled corticosteroid therapy.	cross sectional	2 years	MMAS-8	N/A	asthma patients	217	inhaled corticosteroid (ICS) alone or combination with long-acting beta blocker (LABA)	implementation
2010	Mwenee, M.D	Zambia	To determine the prevalence of drug adherence and factors associated with poor adherence to antihypertensive treatment among adults seen in the department of medicine at UTH	cross sectional	1.5 months	Hill-Bone medication adherence scale	98.7%	hypertension patients	237	anti hypertensive	implementation
2014	Sharma, T.	India	to evaluate the patient adherence to the treatment prescription and to analyse the reasons for non-adherence	cross sectional	8 months	MMAS-4	N/A	Diabetic patients	600	antidiabetic	implementation
2014	Arias, M.F.	Peru	To characterize adherence to pharmacological medication and beliefs towards medication in a group of patients with hypertension in a large national hospital.	cross sectional	2 months	MMAS-8	95.8%	hypertension patients	120	anti hypertensive	implementation

2013	Ledur, P.S.	Brazil	to assess the association of adherence to antihypertensive drug treatment with patients' perception of uncontrolled BP in a sample of diabetic hypertensive subjects attending an outpatient clinic.	cross sectional	9 months	MMAS-4	86.9%	patients with diabetes and hypertension.	323	anti diabetic and anti hypertensive	implementation
2016	Saarti, S.	Lebanon	to examine the association between adherence to medication, treatment satisfaction and illness perception in Lebanese hypertensive patients	cross sectional	7 months	MMAS-8	97.5%	hypertension patients	120	anti hypertensive	implementation
2019	Asgari, M.R.	Iran	to investigate the psychosocial factors associated with treatment adherence in patients with hypertension in Semnan, Iran	cross sectional	N/A	MMAS-8	N/A	hypertension patients	250	anti hypertensive	implementation
2015	Bhandari, B.	Nepal	to explore the extent of adherence towards prescribed antihypertensive treatment and to identify the factors of non adherence	cross sectional	6 months	MMAS-4	N/A	hypertension patients	154	anti hypertensive	implementation
2016	Shams, N.	Pakistan	to determine drug non-adherence in type 2 diabetics and its predictors and associations.	cross sectional	10 months	MMAS-8	N/A	T2DM patients	183	anti diabetic	implementation
2017	Teshome, D.F.	Ethiopia	to assess adherence to antihypertensive medications and identify associated factors at Debre Tabor General Hospital, northwest	cross sectional	3 months	MMAS-4	97.3%	hypertension patients	337	anti hypertensive	implementation

			Ethiopia.								
201 5	Yue, Z.	China	to investigate a range of risk factors associated with medication adherence among Chinese hypertensive patients.	cross sectional	9 month s	MMAS-8	90.6%	hypertensio n patients	256	anti hypertensive	implementatio n
201 8	Zhang, Y.	China	to assess the relationship between factors and medication adherence in Xinjiang community-managed patients with hypertension based on the principal component analysis.	cross sectional	5 month s	no name	87.4%	hypertensio n patients	127	anti hypertensive	implementatio n

Supplementary 5. Summary of Characteristics of Self-Reported Medication Adherence Instruments

Instrument name	Number of items	Type of scoring	Country of Development	Original Language	Fee for Using Applies
ARMS	12	4 Likert scale, with responses of “none”, “some”, “most”, or “all the time”, which are given values of 1–4. The range of possible scores is 12 to 48, with lower scores indicating better adherence. ARMS scores were categorized into: “adherent” with scores 12 or 13 and “non-adherent” with scores of 14 to 48	United States	English	Yes
BMQ	9	Participants receive a score of 1 if their response indicates potential non-adherence and 0 if it indicates adherence	United States	English	No
DAI	10	scoring type was true (score=1) and false (score= -1) for each statement. The scale measured adherence from a maximum of 10 to a minimum of -10. Any negative score was rated as poor adherence, 0 - 5 as moderate adherence and 6 - 10 as good adherent.	United Kingdom	English	No
Hill-Bone medication adherence scale	14	4-point Likert scale (1= all of the time; 2 = most of the time; 3 = some of the time; 4=never). overall adherence score of 14 to 56, with higher scores indicating better adherence. A score ≥48 is considered as adherence and a score <48 is considered as non-adherence.	United States	English	No

MALMAS	8	The first item of the MALMAS has five responses: (1) All the time, (2) Often, (3) Sometimes, (4) Rarely and (5) Never. The remaining seven items have a dichotomous response of “Yes” or “No”. MALMAS scores range from 0 to 8, medication adherence was classified into medication adherence (total score, 6–8) and nonadherence (total score > 6).	Malaysia	English	No
MARS-10	10	5-point Likert scale (never = 5, rarely = 4, sometimes = 3, often = 2, and always = 1). The score obtained from this questionnaire is divided into 10 and thus the scores fit between the ranges of1 to 5. Any score greater than 4.5 indicates good medication adherence and any score below 4.5 indicates poor medication adherence.	Australia	English	No
MARS-5	5	5-point Likert scale, where 5, “never”;4, “rarely”;3, “sometimes”;2, “often” and 1, “always”	Australia	English	No
MARS-A	10	5-point Likert scale (never = 5, rarely = 4, sometimes = 3, often = 2, and always = 1). The score obtained from this questionnaire is divided into 10 and thus the scores fit between the ranges of1 to 5. Any score greater than 4.5 indicates good medication adherence and any score below 4.5 indicates poor medication adherence.	United States	English	No
MASES	26	4 likert scale scoring from 1 (not at all sure), 2 (a little sure), 3 (fairly sure), 4 (extremely sure)	United States	English	NR

MASES-SF	13	4 likert scale scoring from 1 (not at all sure), 2 (a little sure), 3 (fairly sure), 4 (extremely sure). The scoring in the scale is from 1 to 4, the lowest total score obtainable being 13 and the highest 52. Higher scores indicate better compliance of the patient with antihypertensive drug therapy.	United States	English	NR
MTA	7	6Likert-type scale for each question (always (1); almost always (2); frequently (3); sometimes (4); rarely (5); and never (6))	Portugal	Portuguese	NR
MAQ	8	The questions had Yes and No responses for each item. A mark of 1 was awarded for a "No" response and zero for a "Yes" response. The possible overall score for the 8-item medication adherence questionnaire ranged from zero to eight. Participants who had a total score of<6 were considered non-adherent and those whose scores were from 6–8 were categorised as adherent to medication.	United States	English	No
MCQ	7	4-point Likert scale for each question was used in the data collection tools (4=never, 3=sometimes (1-4x per month), 2= often (>5x/month or >2x/week), 1=always). A total score for each patient was calculated which could range from 7 (minimum) to 28 (maximum). Adherence was defined as a score of 27 or more while non-adherence was defined by a score less than 27.	Malaysia	English	No
MMAS-4	4	dichotomous scoring yes or no questions. The number of patients who said 'No' to all four questions were considered adherent	United States	English	Yes

MMAS-6	6	The questions require Yes/No answers. “Yes” produces 1 point, “No” 0 point in questions 2 and 5, and “Yes” produces 0 point and “No” 1 point in other questions	Turkey	English	NR
MMAS-7	7	six-questions have type responses: yes, no, and cannot remember and one multiple choice question	Sri Lanka	English	NR
MMAS-8	8	the first 7 of which are yes/no questions, and the last of which is a 5 point Likert-scale rating (1=Never; 0.75=Rarely once; 0.5=In a while sometimes; 0.25=Usually; 0=All the time). MMAS-8 scores range from 0 to 8, with MMAS-8 score of <6 indicates low adherence, a score of 6 to <8 indicates medium adherence, and a score of 8 indicates high adherence	United States	English	Yes
Self Care Inventory (SCI)	14	5 likert scale. Answers were scored on a 5-point Likert scale with 1 representing “never” and 5 representing “always.” Good adherence was defined by participants responding with 5 (“always”) or 4 (“almost always”)	United States	English	NR
Self Reported Compliance Test	3	dichotomous scoring yes or no questions. All the questions are given a score of 1 for yes. Among these three questions we defined the compliance with treatment as total score of two items including a positive response of the first question	Bangladesh	N/A	NR

TASHP	25	5-point Likert response scale: (1) never/very rarely, (2) rarely, (3) sometimes, (4) mostly, and (5) all of the time, with 1 to 5 points assigned. Possible scores range from 25 to 125, with a higher score indicating greater adherence. A cutoff score of 109 was used to distinguish low adherence behaviors	China	China	NR
No Name (1) Sevilla	10	4 likert scale, Answers ranged from 1 to 4, with increasing scores for better adherence	Mexico	N/A	NR
No Name (2) Karakurt	2	N/A	Turkey	N/A	NR
No Name (3) Hassan	10	5 likert scale Possible scores on the scale ranged from 1 to 5, with 1 indicating ‘never’ and 5 indicating ‘very frequent’. All negatively worded scores were reversed and all scores were converted to a 0 to 100 scale. Patients were categorized as ‘compliant’ if they had an individual score of 75% or greater	Malaysia	N/A	NR
No Name (4) Owolabi	7	dichotomous scoring yes or no questions.	South Africa	N/A	NR
No Name (5) shen	5	The first two items were dichotomous and coded as 0 and 1. The rest three items used Likert 5-point scale and coded as 0, 0.25, 0.50, 0.75, and 1, respectively. Higher scores represented higher MA.	China	N/A	NR
No Name (6) Nesari	68	the questions related to their adherence to the therapeutic regimen in a five-point Likert scale.	Iran	N/A	NR

No Name (7) Bijam	8	5-point Likert scale: No=1, Rarely=2, Occasionally=3, Usually=4, and Always=5.	Iran	N/A	NR
No Name (8) gelaw	6	4 likert scale which is 4: never; 3: rarely; 2: frequently; 1: daily	Ethiopia	N/A	NR
No Name (9) okoro	6	2-point Likert scale used, a mark of two (2) was awarded for Yes, and one (1) for No when they are expected; a mark of one (1) was for Yes, and two (2) for No when they are not expected; a mark of zero (0) was awarded for no response	Nigeria	N/A	NR
no name (10) atulomah	35	4-point scale which require respondents to answer: 0 = all of the time, 1 = most of the time, 2 = some of the time, 3 = none of the time	Nigeria	N/A	NR
no name (11) zhang	4	4-likert scale (1) never complete, 2) sometimes complete, 3) usually complete, and 4) always complete), the participants who chose either the third or the fourth options as answers to all four questions were identified as adherent	China	China	NR
no name (12) iloh	5	4-likert scale using an ordinal scoring system of 0-4 points as follows all-times=4 points, most-times=3 points, sometimes=2 points, rarely=1 point, never=0 point.	Nigeria	N/A	NR

ARMS: Adherence to Refills and Medications Scale; BMQ: Brief Medication Questionnaire; DAI: Drug Attitude Inventory; MALMAS: Malaysian Medication Adherence Scale; MARS-10: Medication Adherence Report Scale-10; MARS-5: Medication Adherence Report Scale-5; MASES: Medication Adherence Self-Efficacy Scale; MASES-SF: Medication Adherence Self-Efficacy Scale-Short Form; MTA: Measure Treatment Adherence; MAQ: Medication Adherence Questionnaire; MCQ: Medication Compliance Questionnaire; MMAS-4: Morisky Medication Adherence Scale-4; MMAS-6: Morisky Medication Adherence Scale-6; MMAS-7: Morisky Medication Adherence Scale-7; MMAS-8: Morisky Medication Adherence Scale-8; SCID: Self Care Inventory Diabetes; TASHP: Therapeutic Adherence Scale For Hypertensive Patients

Supplementary 6. Characteristics of Self-Reported Medication Adherence Instruments

authors	Instrument name	Number of items	Type of scoring	Original language	Translated/ Validated/ Developed	Reasons of non-adherence assessed	Psychometric Properties
Zyoud, S.H	MMAS-8	8	the first 7 of which are yes/no questions, and the last of which is a 5 point Likert-scale rating (1=Never; 0.75=Rarely once; 0.5=In a while sometimes; 0.25=Usually; 0=All the time). MMAS-8 scores range from 0 to 8, with MMAS-8 score of <6 indicates low adherence, a score of 6 to <8 indicates medium adherence, and a score of 8 indicates high adherence	English	translated to Arab and has been validated	**forgetfulness**: point 1, 2, 8 (memory difficulties); point 4 (travelling), point 5, 7 (not able to make taking a drug part of a routine); **lack of knowledge**: point 6 (do not understand why to take the drug); **lack of motivation**: point 3 (high concern)	Cronbach's alpha value 0.723

Sweileh, W. M	MMAS-8	8	the first 7 of which are yes/no questions, and the last of which is a 5 point Likert-scale rating (1=Never; 0.75=Rarely once; 0.5=In a while sometimes; 0.25=Usually; 0=All the time). MMAS-8 scores range from 0 to 8, with MMAS-8 score of <6 indicates low adherence, a score of 6 to <8 indicates medium adherence, and a score of 8 indicates high adherence	English	translated to Arab but has not been validated yet	**forgetfulness**: point 1, 2, 8 (memory difficulties); point 4 (travelling), point 5, 7 (not able to make taking a drug part of a routine); **lack of knowledge**: point 6 (do not understand why to take the drug); **lack of motivation**: point 3 (high concern)	N/A
Shakarneh	MMAS-4	4	dichotomous scoring yes or no questions. The number of patients who said ‘No’ to all four questions were considered adherent	English	N/A	**forgetfulness**: point 1, 2, (memory difficulties) **lack of knowledge**: point 3 (do not understand why to take the drug); **lack of motivation**: point 4 (high concern)	N/A

Al-Ramahi, R.	MMAS-8	8	the first 7 of which are yes/no questions, and the last of which is a 5 point Likert-scale rating (1=Never; 0.75=Rarely once; 0.5=In a while sometimes; 0.25=Usually; 0=All the time). MMAS-8 scores range from 0 to 8, with MMAS-8 score of <6 indicates low adherence, a score of 6 to <8 indicates medium adherence, and a score of 8 indicates high adherence	English	has been translated to Arabic	**forgetfulness**: point 1, 2, 8 (memory difficulties); point 4 (travelling), point 5, 7 (not able to make taking a drug part of a routine); **lack of knowledge**: point 6 (do not understand why to take the drug); **lack of motivation**: point 3 (high concern)	N/A
Khdour, M.R.	MMAS-4	4	dichotomous scoring yes or no questions. The number of patients who said 'No' to all four questions were considered adherent	English	has been translated to Arab and has been validated	**forgetfulness**: point 1, 2, (memory difficulties) **lack of knowledge**: point 3 (do not understand why to take the drug); **lack of motivation**: point 4 (high concern)	Cronbach's alpha value was 0.61
Elsous, A.	Morisky Medication Adherence Scale (MMAS-4)	4	dichotomous scoring yes or no questions. The number of patients who said 'No' to all four questions were considered adherent	English	has been translated to Arab and has been validated	**forgetfulness**: point 1, 2, (memory difficulties) **lack of knowledge**: point 3 (do not understand why to take the drug); **lack of motivation**: point 4 (high concern)	Cronbach's alpha value was 0.76; item and scale content validity index (I-CVI) 0.88-1.00 dan (S-CVI) 0.97-1.00.

							inter reliability kappa statistic > 0.889
Salama, H.M	MMAS-8	8	the first 7 of which are yes/no questions, and the last of which is a 5 point Likert-scale rating (1=Never; 0.75=Rarely once; 0.5=In a while sometimes; 0.25=Usually; 0=All the time). MMAS-8 scores range from 0 to 8, with MMAS-8 score of <6 indicates low adherence, a score of 6 to <8 indicates medium adherence, and a score of 8 indicates high adherence	English	has been translated to Arab and has been validated	**forgetfulness**: point 1, 2, 8 (memory difficulties); point 4 (travelling), point 5, 7 (not able to make taking a drug part of a routine); **lack of knowledge**: point 6 (do not understand why to take the drug); **lack of motivation**: point 3 (high concern)	Cronbach's alpha value was 0.75
Abebe, S.M.	MMAS-8	8	the first 7 of which are yes/no questions, and the last of which is a 5 point Likert-scale rating (1=Never; 0.75=Rarely once; 0.5=In a while sometimes; 0.25=Usually; 0=All	English	N/A	**forgetfulness**: point 1, 2, 8 (memory difficulties); point 4 (travelling), point 5, 7 (not able to make taking a drug part of a routine); **lack of knowledge**: point 6 (do not understand why to take the drug); **lack of motivation**: point 3 (high concern)	N/A

			the time). MMAS-8 scores range from 0 to 8, with MMAS-8 score of <6 indicates low adherence, a score of 6 to <8 indicates medium adherence, and a score of 8 indicates high adherence				
Malik, A.	MMAS-8	8	the first 7 of which are yes/no questions, and the last of which is a 5 point Likert-scale rating (1=Never; 0.75=Rarely once; 0.5=In a while sometimes; 0.25=Usually; 0=All the time). MMAS-8 scores range from 0 to 8, with MMAS-8 score of <6 indicates low adherence, a score of 6 to <8 indicates medium adherence, and a score of 8 indicates high adherence	English	N/A	**forgetfulness**: point 1, 2, 8 (memory difficulties); point 4 (travelling), point 5, 7 (not able to make taking a drug part of a routine);<br>**lack of knowledge**: point 6 (do not understand why to take the drug);<br>**lack of motivation**: point 3 (high concern)	N/A

Jamous, R.M	MMAS-8	8	the first 7 of which are yes/no questions, and the last of which is a 5 point Likert-scale rating (1=Never; 0.75=Rarely once; 0.5=In a while sometimes; 0.25=Usually; 0=All the time). MMAS-8 scores range from 0 to 8, with MMAS-8 score of <6 indicates low adherence, a score of 6 to <8 indicates medium adherence, and a score of 8 indicates high adherence	English	translated to Arab but has not been validated yet	**forgetfulness**: point 1, 2, 8 (memory difficulties); point 4 (travelling), point 5, 7 (not able to make taking a drug part of a routine); **lack of knowledge**: point 6 (do not understand why to take the drug); **lack of motivation**: point 3 (high concern)	N/A
Sarkodie, E.	MMAS-8	8	the first 7 of which are yes/no questions, and the last of which is a 5 point Likert-scale rating (1=Never; 0.75=Rarely once; 0.5=In a while sometimes; 0.25=Usually; 0=All the time). MMAS-8 scores range from 0 to 8, with MMAS-8 score of <6 indicates low adherence, a	English	N/A	**forgetfulness**: point 1, 2, 8 (memory difficulties); point 4 (travelling), point 5, 7 (not able to make taking a drug part of a routine); **lack of knowledge**: point 6 (do not understand why to take the drug); **lack of motivation**: point 3 (high concern)	N/A

			score of 6 to <8 indicates medium adherence, and a score of 8 indicates high adherence				
Nazir, S.U.R	MMAS-8	8	the first 7 of which are yes/no questions, and the last of which is a 5 point Likert-scale rating (1=Never; 0.75=Rarely once; 0.5=In a while sometimes; 0.25=Usually; 0=All the time). MMAS-8 scores range from 0 to 8, with MMAS-8 score of <6 indicates low adherence, a score of 6 to <8 indicates medium adherence, and a score of 8 indicates high adherence	English	has been translated in Urdu and has been validated	**forgetfulness**: point 1, 2, 8 (memory difficulties); point 4 (travelling), point 5, 7 (not able to make taking a drug part of a routine); **lack of knowledge**: point 6 (do not understand why to take the drug); **lack of motivation**: point 3 (high concern)	the Cronbach's alpha value was 0.701. spearman's rank coefficient was value was 0.8. correlation coefficient 0.3-0.4.

Turan, G.B	Medication Adherence Self-Efficacy Scale (MASES)	26	4 likert scale scoring from 1 (not at all sure), 2 (a little sure), 3 (fairly sure), 4 (extremely sure)	English	has been translated to Turkish and has been validated	**forgetfulness**: point 1, 7 (being busy); point 2, 9, 10, 18, 23 (not able to make taking a drug part of a routine); point 3 (lack of family support); point 25 (memories difficulties)<br>**lack of knowledge**: point 14, 20 (do not understand why to take the drug); point 13, 16 (do not understand how to take the drug)<br>**lack of motivation**: point 5, 11, 12, 26 (high concern); point 8 (low necessity)<br>**other drugs-related problem**: point 4, 21 (experience adverse event); point 6, 24 (financial issue); point 19 (polypharmacy); point 22 (access of care)	Cronbach's alpha coefficient was 0.98
Nguyen, T.	MMAS-8	8	the first 7 of which are yes/no questions, and the last of which is a 5 point Likert-scale rating (1=Never; 0.75=Rarely once; 0.5=In a while sometimes; 0.25=Usually; 0=All the time). MMAS-8 scores range from 0 to 8, with MMAS-8 score of <6 indicates low adherence, a score of 6 to <8 indicates medium	English	has been translated in Vietnamese	**forgetfulness**: point 1, 2, 8 (memory difficulties); point 4 (travelling), point 5, 7 (not able to make taking a drug part of a routine);<br>**lack of knowledge**: point 6 (do not understand why to take the drug);<br>**lack of motivation**: point 3 (high concern)	N/A

			adherence, and a score of 8 indicates high adherence				
Cai, Q.	Medication Adherence Report Scale for Asthma (MARS-A)	10	5-point Likert scale ranging from 1 (always) to 5 (never). A mean score was calculated by averaging the 10 questions, ranging from 1 to 5, with MARS-A score ≥4.5 indicating high adherence, and a score <4.5 indicating low adherence	English	has been translated to Chinese and has been validated	**forgetfulness**: point 5 (memory difficulties)<br>**lack of knowledge:** point 1, 2, 6, 10 (do not understand how to take the drug); point 7 (do not understand why to take the drug)<br>**lack of motivation**: point 3, 4, 9 (high concerns); point 8 (low necessity)	Cronbach's α scores of 0.93
Erku, D.A.	MMAS-8	8	the first 7 of which are yes/no questions, and the last of which is a 5 point Likert-scale rating (1=Never; 0.75=Rarely once; 0.5=In a while sometimes; 0.25=Usually; 0=All	English	N/A	**forgetfulness**: point 1, 2, 8 (memory difficulties); point 4 (travelling), point 5, 7 (not able to make taking a drug part of a routine);<br>**lack of knowledge**: point 6 (do not understand why to take the drug);<br>**lack of motivation**: point 3 (high concern)	N/A

			the time). MMAS-8 scores range from 0 to 8, with MMAS-8 score of <6 indicates low adherence, a score of 6 to <8 indicates medium adherence, and a score of 8 indicates high adherence				
Teshome, D.F.	MMAS-4	4	dichotomous scoring yes or no questions. The number of patients who said 'No' to all four questions were considered adherent	English	has been translated to Amharic and has been validated	**forgetfulness**: point 1, 2, (memory difficulties) **lack of knowledge**: point 3 (do not understand why to take the drug); **lack of motivation**: point 4 (high concern)	N/A
Pan, J.	therapeutic adherence scale for hypertensive patients (TASHP)	25	5-point Likert response scale: (1) never/very rarely, (2) rarely, (3) sometimes, (4) mostly, and (5) all of the time, with 1 to 5 points assigned. Possible scores range from 25 to 125, with a higher score indicating greater adherence. A cutoff score of 109 was used to distinguish low adherence behaviors	Chinese	has been validated	**forgetfulness**: point 6-13 **lack of knowledge:** point 1-5 **lifestyle:** point 14-25	Cronbach's a coefficient of the scale was 0.862 and the test- retest reliability coefficient of the total scale was 0.958

Ajayi, D.T	MMAS-8	8	the first 7 of which are yes/no questions, and the last of which is a 5 point Likert-scale rating (1=Never; 0.75=Rarely once; 0.5=In a while sometimes; 0.25=Usually; 0=All the time). MMAS-8 scores range from 0 to 8, with MMAS-8 score of <6 indicates low adherence, a score of 6 to <8 indicates medium adherence, and a score of 8 indicates high adherence	English	has been validated	**forgetfulness**: point 1, 2, 8 (memory difficulties); point 4 (travelling), point 5, 7 (not able to make taking a drug part of a routine);<br>**lack of knowledge**: point 6 (do not understand why to take the drug);<br>**lack of motivation**: point 3 (high concern)	The Cronbach's α was 0.68
Shakya	Hill-Bone medication adherence scale	14	4-point Likert scale (1= all of the time; 2 = most of the time; 3 = some of the time; 4=never). If the score is 14 = perfect adherence. If the score is >14 = non-perfect adherence (score 15–22 = optimal adherence, score >22 = poor adherence)	English	has been translated to Nepalese and has been validated	**forgetfulness**: point 1, 8, 14 (memory difficulties); point 2, 6, 7, 9, 10 (not able to make taking a drug part of routine)<br>**lack of knowledge:** point 11 (do not understand why to take the drug); point 13 (do not understand how to take the drug)<br>**lifestyle**: point 3, 4, 5<br>**lack of motivation:** point 12 (low necessity)	Cronbach alpha was 0.723

Adomako, N.O	MARS-10	10	dichotomous scoring yes or no questions, cored between 0 and 5 had low adherence and 6 and 10 had high adherence	English	translated into the local dialect to the participants as needed.	**forgetfulness**: point 1 (memory difficulties); point 2, 6, 10 (not able to make taking a drug part of routine)<br>**lack of knowledge:** point 3, 5, 8 (do not understand why to take the drug);<br>**lack of motivation:** point 4,7, 9 (high necessity)	N/A
Pan, J.	Hill-Bone medication adherence scale	14	4-point Likert scale (1= all of the time; 2 = most of the time; 3 = some of the time; 4=never). overall adherence score of 14 to 56, with higher scores indicating better adherence. A score ≥48 is considered as adherence and a score <48 is considered as non-adherence.	English	has been translated to Chinese and has been validated	**forgetfulness**: point 1, 8, 14 (memory difficulties); point 2, 6, 7, 9, 10 (not able to make taking a drug part of routine)<br>**lack of knowledge:** point 11 (do not understand why to take the drug); point 13 (do not understand how to take the drug)<br>**lifestyle**: point 3, 4, 5<br>**lack of motivation:** point 12 (low necessity)	The Cronbach's alpha score for the entire questionnaire was 0.857
Aminde, L.N.	Medication Compliance Questionnaire (MCQ)	7	4-point Likert scale for each question was used in the data collection tools (4=never, 3=sometimes (1-4x per month), 2= often (>5x/month or >2x/week), 1=always). A total score for each patient was calculated which	English	N/A	**forgetfulness**: point 1(memory difficulties); point 6 (travelling)<br>**lack of knowledge**: point 3, 4 (do not understand why to take the drug)<br>**lack of motivation**: point 2 (low necessity)<br>**other drugs-related problem**: point 5 (experience adverse event); point 7 (run out drug)	N/A

			could range from 7 (minimum) to 28 (maximum). Adherence was defined as a score of 27 or more while non-adherence was defined by a score less than 27.				
Ahmad, N.S.	Medication Compliance Questionnaire (MCQ)	7	4-point Likert scale for each question was used in the data collection tools (4=never, 3=sometimes (1-4x per month), 2= often (>5x/month or >2x/week), 1=always). A total score for each patient was calculated which could range from 7 (minimum) to 28 (maximum). Adherence was defined as a score of 27 or more while non-adherence was defined by a score less than 27.	English	has been developed using a combination of the Morisky self-reporting scale and the Hill-Bone Compliance to High Blood Pressure Therapy Scale, and has been validated	**forgetfulness**: point 1(memory difficulties); point 6 (travelling) **lack of knowledge**: point 3, 4 (do not understand why to take the drug) **lack of motivation**: point 2 (low necessity) **other drugs-related problem**: point 5 (experience adverse event); point 7 (run out drug)	Cronbach's alpha value of 0.782; a Cohen's kappa statistic value of 0.796

Akintunde, A.A.	MMAS-8	8	the first 7 of which are yes/no questions, and the last of which is a 5 point Likert-scale rating (1=Never; 0.75=Rarely once; 0.5=In a while sometimes; 0.25=Usually; 0=All the time). Medication adherence was categorized as low (score >2), medium(score 1-2), and high adherence (score 0)	English	has been validated (using original language)	**forgetfulness**: point 1, 2, 8 (memory difficulties); point 4 (travelling), point 5, 7 (not able to make taking a drug part of a routine);<br>**lack of knowledge**: point 6 (do not understand why to take the drug);<br>**lack of motivation**: point 3 (high concern)	N/A
Akoko, B.M.	MMAS-8	8	the first 7 of which are yes/no questions, and the last of which is a 5 point Likert-scale rating (1=Never; 0.75=Rarely once; 0.5=In a while sometimes; 0.25=Usually; 0=All the time). A score of less than 3 represented adherence and a score of 3–8 represented nonadherence.	English	has been validated (using original language)	**forgetfulness**: point 1, 2, 8 (memory difficulties); point 4 (travelling), point 5, 7 (not able to make taking a drug part of a routine);<br>**lack of knowledge**: point 6 (do not understand why to take the drug);<br>**lack of motivation**: point 3 (high concern)	N/A

Chan, C.W.	MMAS-8	8	the first 7 of which are yes/no questions, and the last of which is a 5 point Likert-scale rating (1=Never; 0.75=Rarely once; 0.5=In a while sometimes; 0.25=Usually; 0=All the time). MMAS-8 scores range from 0 to 8, with MMAS-8 score of <6 indicates low adherence, a score of 6 to <8 indicates medium adherence, and a score of 8 indicates high adherence	English	has been validated, and has been translated to Malaysian version	**forgetfulness**: point 1, 2, 8 (memory difficulties); point 4 (travelling), point 5, 7 (not able to make taking a drug part of a routine); **lack of knowledge**: point 6 (do not understand why to take the drug); **lack of motivation**: point 3 (high concern)	Cronbach's alpha was 0.675, and the test–retest reliability value was 0.816
Lulebo	MMAS-4	4	dichotomous scoring yes or no questions. The number of patients who said ‘No’ to all four questions were considered adherent	English	has been translated to French and Lingala	**forgetfulness**: point 1, 2, (memory difficulties) **lack of knowledge**: point 3 (do not understand why to take the drug); **lack of motivation**: point 4 (high concern)	N/A
Abbas	MMAS-8	8	the first 7 of which are yes/no questions, and the last of which is a 5 point Likert-scale rating. A score of 0-1 represented high adherence and score of 2 represented	English	has been translated to Urdu and has been validated in pilot study	**forgetfulness**: point 1, 2, 8 (memory difficulties); point 4 (travelling), point 5, 7 (not able to make taking a drug part of a routine); **lack of knowledge**: point 6 (do not understand why to take the drug);	N/A

			medium adherence. Scores 3-7 represented low adherence and a score of 8 meant no adherence			**lack of motivation**: point 3 (high concern)	
Arulmozhi, S.	MMAS-8	8	the first 7 of which are yes/no questions, and the last of which is a 5 point Likert-scale rating (1=Never; 0.75=Rarely once; 0.5=In a while sometimes; 0.25=Usually; 0=All the time). MMAS-8 scores range from 0 to 8, with MMAS-8 score of <6 indicates low adherence, a score of 6 to <8 indicates medium adherence, and a score of 8 indicates high adherence	English	has been validated (in original instruments), and has been translated to India version	**forgetfulness**: point 1, 2, 8 (memory difficulties); point 4 (travelling), point 5, 7 (not able to make taking a drug part of a routine);<br>**lack of knowledge**: point 6 (do not understand why to take the drug);<br>**lack of motivation**: point 3 (high concern)	Cronbach's alph a was 0.83
Ambaw	MMAS-4	4	dichotomous scoring yes or no questions. a cut-off value of MMAS mean score>=3 was used for labeling patients as adherent	English	N/A	**forgetfulness**: point 1, 2, (memory difficulties)<br>**lack of knowledge**: point 3 (do not understand why to take the drug);<br>**lack of motivation**: point 4 (high concern)	N/A

Waari, G.	MMAS-8	8	the first 7 of which are yes/no questions, and the last of which is a 5 point Likert-scale rating (1=Never; 0.75=Rarely once; 0.5=In a while sometimes; 0.25=Usually; 0=All the time). MMAS-8 scores range from 0 to 8, with MMAS-8 score of <6 indicates low adherence, a score of 6 to <8 indicates medium adherence, and a score of 8 indicates high adherence	English	N/A	**forgetfulness**: point 1, 2, 8 (memory difficulties); point 4 (travelling), point 5, 7 (not able to make taking a drug part of a routine);<br>**lack of knowledge**: point 6 (do not understand why to take the drug);<br>**lack of motivation**: point 3 (high concern)	N/A
Ozoh, O.B.	MMAS-8	8	the first 7 of which are yes/no questions, and the last of which is a 5 point Likert-scale rating (1=Never; 0.75=Rarely once; 0.5=In a while sometimes; 0.25=Usually; 0=All the time). MMAS-8 scores range from 0 to 8, with MMAS-8 score of <6 indicates low adherence, a	English	N/A	**forgetfulness**: point 1, 2, 8 (memory difficulties); point 4 (travelling), point 5, 7 (not able to make taking a drug part of a routine);<br>**lack of knowledge**: point 6 (do not understand why to take the drug);<br>**lack of motivation**: point 3 (high concern)	N/A

			score of 6 to <8 indicates medium adherence, and a score of 8 indicates high adherence				
Tefera, Y.G	MMAS-4	4	dichotomous scoring yes or no questions. The number of patients who said 'No' to all four questions were considered adherent	English	has been translated to Amharic and has been validated	**forgetfulness**: point 1, 2, (memory difficulties) **lack of knowledge**: point 3 (do not understand why to take the drug); **lack of motivation**: point 4 (high concern)	N/A
Yuvaraj, K.	MMAS-4	4	dichotomous scoring yes or no questions. The number of patients who said 'No' to all four questions were considered adherent	English	has been translated to Hindi and has been validated (in original version)	**forgetfulness**: point 1, 2, (memory difficulties) **lack of knowledge**: point 3 (do not understand why to take the drug); **lack of motivation**: point 4 (high concern)	N/A
Pirasath, S.	MMAS-7	7	six-questions have type responses: yes, no, and cannot remember and one multiple choice question	English	has been translated to Sinhala and Tamil languages and has been validated in pilot study	**forgetfulness**: not able to make taking a drug part of the routine (points 1,2,3), traveling (point 5), memory difficulties (point 7) **other related problem**: experiencing adverse drug reactions (point 4) **lack of motivation**: low necessity (point 6)	N/A

Abba, Z.I.	MMAS-8	8	the first 7 of which are yes/no questions, and the last of which is a 5 point Likert-scale rating (1=Never; 0.75=Rarely once; 0.5=In a while sometimes; 0.25=Usually; 0=All the time) and a score ≥ 1 defined medication non-adherence.	English	N/A	**forgetfulness**: point 1, 2, 8 (memory difficulties); point 4 (travelling), point 5, 7 (not able to make taking a drug part of a routine); **lack of knowledge**: point 6 (do not understand why to take the drug); **lack of motivation**: point 3 (high concern)	N/A
Dego, T.R.	MMAS-4	4	dichotomous scoring yes or no questions. The number of patients who said 'No' to all four questions were considered adherent	English	N/A	**forgetfulness**: point 1, 2, (memory difficulties) **lack of knowledge**: point 3 (do not understand why to take the drug); **lack of motivation**: point 4 (high concern)	N/A
Shimels, T.	MMAS-8	8	Items 1 through 7 were coded as 1 = yes, 2 = no, except for item 5 in which no was rated as 1 and yes was rated as 2. For item 8, the Likert scaled scores of1 to 5 were reverse coded as 2 = never and 1 = often to always. MMAS-8 score summation of 8 through 15 was	English	has been translated to Amharic and has been validated	**forgetfulness**: point 1, 2, 8 (memory difficulties); point 4 (travelling), point 5, 7 (not able to make taking a drug part of a routine); **lack of knowledge**: point 6 (do not understand why to take the drug); **lack of motivation**: point 3 (high concern)	alpha test ofthe reliability ofthe scale among the samples also showed an acceptable range for both hospitals (α = 0.88) and health centers (α = 0.63).

			classified under poor adherence				
Balasubramanian, A.	MMAS-4	4	dichotomous scoring yes or no questions (no=0, yes=1), total score zero was considered as high adherence, a score of 1 or 2 as medium adherence, and a score of 3 or 4 as low adherence	English	N/A	**forgetfulness**: point 1, 2, (memory difficulties)<br>**lack of knowledge**: point 3 (do not understand why to take the drug);<br>**lack of motivation**: point 4 (high concern)	N/A
Mahmood, S.	MMAS-8	8	the first 7 of which are yes/no questions, and the last of which is a 5 point Likert-scale rating (1=Never; 0.75=Rarely once; 0.5=In a while sometimes; 0.25=Usually; 0=All the time). MMAS-8 scores range from 0 to 8, with MMAS-8 score of <6 indicates low adherence, a score of 6 to <8	English	has been translated to Urdu and has been validated	**forgetfulness**: point 1, 2, 8 (memory difficulties); point 4 (travelling), point 5, 7 (not able to make taking a drug part of a routine);<br>**lack of knowledge**: point 6 (do not understand why to take the drug);<br>**lack of motivation**: point 3 (high concern)	Cronbach's alpha was 0.701 and the test–retest reliability value was 0.8

			indicates medium adherence, and a score of 8 indicates high adherence				
Chaudhary, A.P.	Hill-Bone medication adherence scale	14	4-point Likert scale (1= all of the time; 2 = most of the time; 3 = some of the time; 4=never). overall adherence score of 14 to 56, with higher scores indicating better adherence. A score ≥48 is considered as adherence and a score <48 is considered as non-adherence.	English	N/A	**forgetfulness**: point 1, 8, 14 (memory difficulties); point 2, 6, 7, 9, 10 (not able to make taking a drug part of routine)<br>**lack of knowledge:** point 11 (do not understand why to take the drug); point 13 (do not understand how to take the drug)<br>**lifestyle**: point 3, 4, 5<br>**lack of motivation:** point 12 (low necessity)	N/A
Mathur, D.	MMAS-8	8	the first 7 of which are yes/no questions, and the last of which is a 5 point Likert-scale rating (1=Never; 0.75=Rarely once; 0.5=In a while sometimes; 0.25=Usually; 0=All the time). MMAS-8 scores range from 0	English	N/A	**forgetfulness**: point 1, 2, 8 (memory difficulties); point 4 (travelling), point 5, 7 (not able to make taking a drug part of a routine);<br>**lack of knowledge**: point 6 (do not understand why to take the drug);<br>**lack of motivation**: point 3 (high concern)	N/A

			to 8, with MMAS-8 score of <6 indicates low adherence, a score of 6 to <8 indicates medium adherence, and a score of 8 indicates high adherence				
Kassahun, A.	MMAS-4	4	dichotomous scoring yes or no questions. The number of patients who said ‘No’ to all four questions were considered adherent	English	N/A	**forgetfulness**: point 1, 2, (memory difficulties)<br>**lack of knowledge**: point 3 (do not understand why to take the drug);<br>**lack of motivation**: point 4 (high concern)	N/A
Okeke, H.C.	MMAS-8	8	the first 7 of which are yes/no questions, and the last of which is a 5 point Likert-scale rating. MMAs scores range from zero to eight with low adherence defined as MMAs scores < 2; moderate adherence as 1-2 and optimal adherence scores as a score of 0	English	N/A	**forgetfulness**: point 1, 2, 8 (memory difficulties); point 4 (travelling), point 5, 7 (not able to make taking a drug part of a routine);<br>**lack of knowledge**: point 6 (do not understand why to take the drug);<br>**lack of motivation**: point 3 (high concern)	N/A

Afaya, R.A.	Medication Adherence Questionnaire (MAQ)	8	The questions had Yes and No responses for each item. A mark of 1 was awarded for a “No” response and zero for a “Yes” response. The possible overall score for the 8-item medication adherence questionnaire ranged from zero to eight. Participants who had a total score of<6 were considered non-adherent and those whose scores were from 6–8 were categorised as adherent to medication.	English	has been translated to Dagbani and has been validated. The questionnaire was developed from previous instrument including MARS, Hill-Bone, and MMAS.	assessed deliberate and non-deliberate non-adherence including reasons for non-adherence<br>**forgetfulness**: point 1, 8 (memory difficulties); point 4 (travelling), point 5, 7(not able to make as a routine activity)<br>**lack of motivation**: point 2, 6 (low necessity)<br>**other drugs-related problem**: point 3 (experience adverse event)	The Cronbach alpha was 0.765
Iloh, G.U.P	Measure Treatment Adherence (MTA)	5	4-likert scale using an ordinal scoring system of 0-4 points as follows all-times=4 points, most-times=3 points, sometimes=2 points, rarely=1 point, never=0 point.	Portugal	has been developed by the author from several literature and has been validated in pilot testing	point 1-5: forgetfulness	N/A

Takahashi, E.	MMAS-4	4	dichotomous scoring yes or no questions (no=0, yes=1), total score zero was considered as high adherence, a score of 1 or 2 as medium adherence, and a score of 3 or 4 as low adherence	English	has been translated to Laotian	**forgetfulness**: point 1, 2, (memory difficulties)<br>**lack of knowledge**: point 3 (do not understand why to take the drug);<br>**lack of motivation**: point 4 (high concern)	N/A
Amer, M.	MMAS-8	8	the first 7 of which are yes/no questions, and the last of which is a 5 point Likert-scale rating (1=Never; 0.75=Rarely once; 0.5=In a while sometimes; 0.25=Usually; 0=All the time). MMAS-8 scores range from 0 to 8, with MMAS-8 score of <6 indicates low adherence, a score of 6 to <8 indicates medium adherence, and a score of 8 indicates high adherence	English	has been translated to Urdu and has been validated	**forgetfulness**: point 1, 2, 8 (memory difficulties); point 4 (travelling), point 5, 7 (not able to make taking a drug part of a routine);<br>**lack of knowledge**: point 6 (do not understand why to take the drug);<br>**lack of motivation**: point 3 (high concern)	Cronbach's alpha value was 0.747

Nonogaki, A.	MMAS-4	4	dichotomous scoring yes or no questions (no=0, yes=1), total score zero was considered as high adherence, a score of 1 or 2 as medium adherence, and a score of 3 or 4 as low adherence	English	has been translated to Khmer and has been validated	**forgetfulness**: point 1, 2, (memory difficulties)<br>**lack of knowledge**: point 3 (do not understand why to take the drug);<br>**lack of motivation**: point 4 (high concern)	The Cron-bach's coefficient alpha of the scale in this study was 0.78
Raja, W.	Hill-Bone medication adherence scale	14	4-point Likert scale (1= all of the time; 2 = most of the time; 3 = some of the time; 4=never). overall adherence score of 14 to 56, with higher scores indicating better adherence. A score ≥48 is considered as adherence and a score <48 is considered as non-adherence.	English	N/A	**forgetfulness**: point 1, 8, 14 (memory difficulties); point 2, 6, 7, 9, 10 (not able to make taking a drug part of routine)<br>**lack of knowledge:** point 11 (do not understand why to take the drug); point 13 (do not understand how to take the drug)<br>**lifestyle**: point 3, 4, 5<br>**lack of motivation:** point 12 (low necessity)	N/A
Ali, M.	MMAS-8	8	the first 7 of which are yes/no questions, and the last of which is a 5 point Likert-scale rating. MMAS-8 score of 0 indicates adherence, a score of 1-8 indicates non-adherence	English	N/A	**forgetfulness**: point 1, 2, 8 (memory difficulties); point 4 (travelling), point 5, 7 (not able to make taking a drug part of a routine);<br>**lack of knowledge**: point 6 (do not understand why to take the drug);<br>**lack of motivation**: point 3 (high concern)	N/A

Ullah	Hill-Bone medication adherence scale	14	4-point Likert scale (1= all of the time; 2 = most of the time; 3 = some of the time; 4=never). overall adherence score of 14 to 56, with higher scores indicating better adherence. A score ≥48 is considered as adherence and a score <48 is considered as non-adherence.	English	N/A	**forgetfulness**: point 1, 8, 14 (memory difficulties); point 2, 6, 7, 9, 10 (not able to make taking a drug part of routine)<br>**lack of knowledge:** point 11 (do not understand why to take the drug); point 13 (do not understand how to take the drug)<br>**lifestyle**: point 3, 4, 5<br>**lack of motivation:** point 12 (low necessity)	N/A
G/Tsadik, D.	MMAS-8	8	the first 7 of which are yes/no questions, and the last of which is a 5 point Likert-scale rating (1=Never; 0.75=Rarely once; 0.5=In a while sometimes; 0.25=Usually; 0=All the time). MMAS-8 scores range from 0 to 8, with MMAS-8 score of <6 indicates low adherence, a score of 6 to <8 indicates medium adherence, and a score of 8 indicates high adherence	English	has been validated	**forgetfulness**: point 1, 2, 8 (memory difficulties); point 4 (travelling), point 5, 7 (not able to make taking a drug part of a routine);<br>**lack of knowledge**: point 6 (do not understand why to take the drug);<br>**lack of motivation**: point 3 (high concern)	Cronbach's alpha value was 0.72

Roka	MMAS-4	4	dichotomous scoring yes or no questions (no=0, yes=1), total score zero was considered as high adherence, a score of 1 or 2 as medium adherence, and a score of 3 or 4 as low adherence	English	N/A	**forgetfulness**: point 1, 2, (memory difficulties) **lack of knowledge**: point 3 (do not understand why to take the drug); **lack of motivation**: point 4 (high concern)	N/A
Bakar, Z.A	MMAS-8	8	the first 7 of which are yes/no questions, and the last of which is a 5 point Likert-scale rating (1=Never; 0.75=Rarely once; 0.5=In a while sometimes; 0.25=Usually; 0=All the time). MMAS-8 scores range from 0 to 8, with MMAS-8 score of <6 indicates low adherence, a score of 6 to <8 indicates medium adherence, and a score of 8 indicates high adherence	English	has been translated to Malay and has been validated	**forgetfulness**: point 1, 2, 8 (memory difficulties); point 4 (travelling), point 5, 7 (not able to make taking a drug part of a routine); **lack of knowledge**: point 6 (do not understand why to take the drug); **lack of motivation**: point 3 (high concern)	Cronbach’s alpha value was 0.675

Yang, S.	MMAS-4	4	dichotomous scoring yes or no questions. The number of patients who said 'No' to all four questions were considered adherent	English	has been translated to Chinese and has been validated	**forgetfulness**: point 1, 2, (memory difficulties)<br>**lack of knowledge**: point 3 (do not understand why to take the drug);<br>**lack of motivation**: point 4 (high concern)	Cronbach's alpha value was 0.712
Berhe, D.F.	MMAS-8	8	the first 7 of which are yes/no questions, and the last of which is a 5 point Likert-scale rating (1=Never; 0.75=Rarely once; 0.5=In a while sometimes; 0.25=Usually; 0=All the time). MMAS-8 scores range from 0 to 8, with MMAS-8 score of <6 indicates low adherence, a score of 6 to <8 indicates medium adherence, and a score of 8 indicates high adherence	English	has been translated to Amharic and Tigrigna and has been validated	**forgetfulness**: point 1, 2, 8 (memory difficulties); point 4 (travelling), point 5, 7 (not able to make taking a drug part of a routine);<br>**lack of knowledge**: point 6 (do not understand why to take the drug);<br>**lack of motivation**: point 3 (high concern)	Cronbach's alpha value was 0.72

Sefah, I.A.	MMAS-8	8	the first 7 of which are yes/no questions, and the last of which is a 5 point Likert-scale rating (1=Never; 0.75=Rarely once; 0.5=In a while sometimes; 0.25=Usually; 0=All the time). MMAS-8 scores range from 0 to 8, with MMAS-8 score of <6 indicates low adherence, a score of 6 to <8 indicates medium adherence, and a score of 8 indicates high adherence	English	N/A	**forgetfulness**: point 1, 2, 8 (memory difficulties); point 4 (travelling), point 5, 7 (not able to make taking a drug part of a routine); **lack of knowledge**: point 6 (do not understand why to take the drug); **lack of motivation**: point 3 (high concern)	N/A
Saraiva, E.M.S	MMAS-4	4	dichotomous scoring yes or no questions. The number of patients who said 'No' to all four questions were considered adherent	English	has been translated to Portuguese and has been validated	**forgetfulness**: point 1, 2, (memory difficulties) **lack of knowledge**: point 3 (do not understand why to take the drug); **lack of motivation**: point 4 (high concern)	Cronbach's alpha value was 0.73

Bermeo-Cabrera, J.	MMAS-8	8	the first 7 of which are yes/no questions, and the last of which is a 5 point Likert-scale rating (1=Never; 0.75=Rarely once; 0.5=In a while sometimes; 0.25=Usually; 0=All the time). MMAS-8 scores range from 0 to 8, with MMAS-8 score of <6 indicates low adherence, a score of 6 to <8 indicates medium adherence, and a score of 8 indicates high adherence	English	N/A	**forgetfulness**: point 1, 2, 8 (memory difficulties); point 4 (travelling), point 5, 7 (not able to make taking a drug part of a routine);<br>**lack of knowledge**: point 6 (do not understand why to take the drug);<br>**lack of motivation**: point 3 (high concern)	N/A
Lerman, I.	Self Care Inventory Diabetes	15	5 likert scale. Answers were scored on a 5-point Likert scale with 1 representing “never” and 5 representing “always.” Good adherence was defined by participants responding with 5 (“always”) or 4 (“almost always”)	English	N/A	Thus, 4 of the items address diet, 2 address glucose monitoring, 3 address medication administration, 1 addresses exercise, 2 address low glucose levels, and the remaining 3 items address preventative/routine aspects of self-care	N/A

Abate, T.W.	MMAS-8	8	the first 7 of which are yes/no questions (yes=1 no=0), and the last of which is a 5 point Likert-scale rating (1=Never; 0.75=Rarely once; 0.5=In a while sometimes; 0.25=Usually; 0=All the time). A score of > 2 was considered as low adherence, 0-2 considered as high adherence	English	has been translated to Amharic	**forgetfulness**: point 1, 2, 8 (memory difficulties); point 4 (travelling), point 5, 7 (not able to make taking a drug part of a routine); **lack of knowledge**: point 6 (do not understand why to take the drug); **lack of motivation**: point 3 (high concern)	N/A
Oluwole, E.O	MMAS-8	8	the first 7 of which are yes/no questions, and the last of which is a 5 point Likert-scale rating (1=Never; 0.75=Rarely once; 0.5=In a while sometimes; 0.25=Usually; 0=All the time). A score of 0 was considered as high adherence, a score of 1–4 was considered as moderate adherence and a score of >4 as low adherence. The	English	has been translated to Yoruba and Pidgin English; and has been validated	**forgetfulness**: point 1, 2, 8 (memory difficulties); point 4 (travelling), point 5, 7 (not able to make taking a drug part of a routine); **lack of knowledge**: point 6 (do not understand why to take the drug); **lack of motivation**: point 3 (high concern)	Cronbach's alpha value was 0.69

Mariye, T.	MMAS-8	8	the first 7 of which are yes/no questions (no=1, yes=0), and the last of which is a 5 point Likert-scale rating (1=Never; 0.75=Rarely once; 0.5=In a while sometimes; 0.25=Usually; 0=All the time). MMAS-8 scores range from 0 to 8, with MMAS-8 score of <6 indicates non adherent, a score of >6 t adherent	English	has been translated to Tigrigna	**forgetfulness**: point 1, 2, 8 (memory difficulties); point 4 (travelling), point 5, 7 (not able to make taking a drug part of a routine);<br>**lack of knowledge**: point 6 (do not understand why to take the drug);<br>**lack of motivation**: point 3 (high concern)	N/A
Jackson, I.	MMAS-8	8	the first 7 of which are yes/no questions, and the last of which is a 5 point Likert-scale rating (1=Never; 0.75=Rarely once; 0.5=In a while sometimes; 0.25=Usually; 0=All the time). MMAS-8 scores range from 0 to 8, with MMAS-8 score of <6 indicates low adherence, a score of 6 to <8 indicates medium adherence, and a score of 8 indicates	English	N/A (using original language)	**forgetfulness**: point 1, 2, 8 (memory difficulties); point 4 (travelling), point 5, 7 (not able to make taking a drug part of a routine);<br>**lack of knowledge**: point 6 (do not understand why to take the drug);<br>**lack of motivation**: point 3 (high concern)	N/A

			high adherence				
Basharat	MMAS-8	8	the first 7 of which are yes/no questions (no=1, yes=0), and the last of which is a 5 point Likert-scale rating (1=Never; 0.75=Rarely once; 0.5=In a while sometimes; 0.25=Usually; 0=All the time). MMAS-8 scores range from 0 to 8, with MMAS-8 score of <6 indicates low adherence, a score of 6 to <8 indicates medium adherence, and a score of 8 indicates high adherence	English	N/A	**forgetfulness**: point 1, 2, 8 (memory difficulties); point 4 (travelling), point 5, 7 (not able to make taking a drug part of a routine);<br>**lack of knowledge**: point 6 (do not understand why to take the drug);<br>**lack of motivation**: point 3 (high concern)	N/A

Fadare, J.	MMAS-8	8	the first 7 of which are yes/no questions (no=1, yes=0), and the last of which is a 5 point Likert-scale rating (1=Never; 0.75=Rarely once; 0.5=In a while sometimes; 0.25=Usually; 0=All the time). MMAS-8 scores range from 0 to 8, with MMAS-8 score of <6 indicates low adherence, a score of 6 to <8 indicates medium adherence, and a score of 8 indicates high adherence	English	N/A	**forgetfulness**: point 1, 2, 8 (memory difficulties); point 4 (travelling), point 5, 7 (not able to make taking a drug part of a routine);<br>**lack of knowledge**: point 6 (do not understand why to take the drug);<br>**lack of motivation**: point 3 (high concern)	N/A
Al-Qerem, W.	BMQ and MMAS-4	4	dichotomous scoring yes or no questions (yes=1, no=0). Low adherence for the participants with a score of >3, moderate adherence score 1-2, and high adherence group for those with a score of 0	English	has been translated to Arabic and has been validated	**forgetfulness**: point 1, 2, (memory difficulties)<br>**lack of knowledge**: point 3 (do not understand why to take the drug);<br>**lack of motivation**: point 4 (high concern)	Cronbach's alpha value was 0.747

Iqbal	Drug Attitude Inventory (DAI)	10	scoring type was true (score=1) and false (score= -1) for each statement. The scale measured adherence from a maximum of 10 to a minimum of -10. Any negative score was rated as poor adherence, 0 - 5 as moderate adherence and 6 - 10 as good adherent.	English	has been translated to Urdu and has been validated	**lack of motivation** : point 1, 9 (high necessity), point 3 (low necessity)<br>**forgetfulness**: point 8 (make a daily routine)<br>**lack of knowledge**: point 6, 7, 10 (do not understand why to take drug)<br>**other drug-related problems:** point 2, 5 (experience adverse event)	Cronbach's alpha value was 0.65
Akram, F.	MMAS-8	8	the first 7 of which are yes/no questions (no=1, yes=0), and the last of which is a 5 point Likert-scale rating (1=Never; 0.75=Rarely once; 0.5=In a while sometimes; 0.25=Usually; 0=All the time). MMAS-8 scores range from 0 to 8, with MMAS-8 score of <6 indicates low adherence, a score of 6 to <8 indicates medium adherence, and a score of 8 indicates high adherence	English	has been translated to Urdu and has been validated	**forgetfulness**: point 1, 2, 8 (memory difficulties); point 4 (travelling), point 5, 7 (not able to make taking a drug part of a routine);<br>**lack of knowledge**: point 6 (do not understand why to take the drug);<br>**lack of motivation**: point 3 (high concern)	Cronbach's alpha value was 0.70

Ayodapo, A.O.	MMAS-4	4	dichotomous scoring yes or no questions (yes=1, no=0). Low adherence for the participants with a score of >3, moderate adherence group for those with 1-2 points, and high adherence group for those with a score of 0	English	has been translated to Yoruba	**forgetfulness**: point 1, 2, (memory difficulties)<br>**lack of knowledge**: point 3 (do not understand why to take the drug);<br>**lack of motivation**: point 4 (high concern)	N/A
Hashmi	MMAS-4	4	dichotomous scoring yes or no questions (yes=0, no=1). The total score ranges from 0 (non adherent) to 4 (adherent)	English	has been translated to Urdu	**forgetfulness**: point 1, 2, (memory difficulties)<br>**lack of knowledge**: point 3 (do not understand why to take the drug);<br>**lack of motivation**: point 4 (high concern)	N/A
Asilar, H.	Medication Adherence Self-Efficacy Scale Short Form (MASES-SF)	13	4 likert scale scoring from 1 (not at all sure), 2 (a little sure), 3 (fairly sure), 4 (extremely sure). The scoring in the scale is from 1 to 4, the lowest total score obtainable being 13 and the highest 52. Higher scores indicate better	English	has been translated to Turkish and has been validated	**forgetfulness**: point 1, 7 (being busy); point 5, 6, 9, 10, (not able to make taking a drug part of a routine); point 2 (lack of family support); point 25 (memories difficulties); point 8 (travelling)<br>**lack of knowledge**: point 11 (do not understand why to take the drug)<br>**lack of motivation**: point 3 (high concern); point 4 (low necessity)<br>**other drugs-related problem**:	Cronbach's alpha coefficient was 0.97

			compliance of the patient with antihypertensive drug therapy.			point 12 (experience adverse event)	
Kassahun, T.	MMAS-8	8	the first 7 of which are yes/no questions (no=0, yes=1), and the last of which is a 5 point Likert-scale rating (1=Never; 0.75=Rarely once; 0.5=In a while sometimes; 0.25=Usually; 0=All the time). The total score of adherence was classified into low adherence if the score was >2, medium adherence if between 1 and 2, and high adherence if 0	English	has been translated to Amharic and Afan-Oromo	**forgetfulness**: point 1, 2, 8 (memory difficulties); point 4 (travelling), point 5, 7 (not able to make taking a drug part of a routine); **lack of knowledge**: point 6 (do not understand why to take the drug); **lack of motivation**: point 3 (high concern)	N/A

Ishaq, R.	Drug Attitude Inventory (DAI)	10	scoring type was true (score=1) and false (score= -1) for each statement. The scale measured adherence from a maximum of 10 to a minimum of -10. Any negative score was rated as poor adherence, 0 - 5 as moderate adherence and 6 - 10 as good adherent.	English	has been translated to Urdu and has been validated	**lack of motivation** : point 1, 9 (high necessity), point 3 (low necessity)<br>**forgetfulness**: point 8 (make a daily routine)<br>**lack of knowledg**e: point 6, 7, 10 (do not understand why to take drug)<br>**other drug-related problems:** point 2, 5 (experience adverse event)	N/A
Ngahane, B.H.M.	MMAS-8	8	the first 7 of which are yes/no questions (no=1, yes=0), and the last of which is a 5 point Likert-scale rating (1=Never; 0.75=Rarely once; 0.5=In a while sometimes; 0.25=Usually; 0=All the time). MMAS-8 scores range from 0 to 8, with MMAS-8 score of <6 indicates low adherence, a score of 6 to <8 indicates medium adherence, and a score of 8 indicates high adherence	English	using original language	**forgetfulness**: point 1, 2, 8 (memory difficulties); point 4 (travelling), point 5, 7 (not able to make taking a drug part of a routine);<br>**lack of knowledge**: point 6 (do not understand why to take the drug);<br>**lack of motivation**: point 3 (high concern)	N/A

Adeoye, A.M	MMAS-4	4	dichotomous scoring yes or no questions. The number of patients who said ‘No’ to all four questions were considered adherent	English	has been translated to local language Nigeria and has been validated	**forgetfulness**: point 1, 2, (memory difficulties) **lack of knowledge**: point 3 (do not understand why to take the drug); **lack of motivation**: point 4 (high concern)	Cronbach’s alpha value was 0.78
Andualem, A.	MMAS-4	4	dichotomous scoring yes or no questions. The number of patients who said ‘No’ to all four questions were considered adherent	English	has been translated to Amharic	**forgetfulness**: point 1, 2, (memory difficulties) **lack of knowledge**: point 3 (do not understand why to take the drug); **lack of motivation**: point 4 (high concern)	Cronbach’s alpha value was 0.61
Hossain, A.	self-reported compliance test (SC)	3	dichotomous scoring yes or no questions. All the questions are given a score of 1 for yes. Among these three questions we defined the compliance with treatment as total score of two items including a positive response of the first question	N/A	has been developed from the Morisky-Green test (MG) and Wong et al.	forgetfulness, irregular clinic attendance, and lack of support family	N/A

Asgedom, S.W	MMAS-8	8	the first 7 of which are yes/no questions (no=1, yes=0), and the last of which is a 5 point Likert-scale rating (1=Never; 0.75=Rarely once; 0.5=In a while sometimes; 0.25=Usually; 0=All the time). MMAS-8 scores range from 0 to 8, with MMAS-8 score of <6 indicates low adherence, a score of 6 to <8 indicates medium adherence, and a score of 8 indicates high adherence	English	has been translated to Amharic and Afan Oromo	**forgetfulness**: point 1, 2, 8 (memory difficulties); point 4 (travelling), point 5, 7 (not able to make taking a drug part of a routine); **lack of knowledge**: point 6 (do not understand why to take the drug); **lack of motivation**: point 3 (high concern)	N/A
Jannoo, Z.	MMAS-8	8	the first 7 of which are yes/no questions (no=1, yes=0), and the last of which is a 5 point Likert-scale rating (1=Never; 0.75=Rarely once; 0.5=In a while sometimes; 0.25=Usually; 0=All the time). MMAS-8 scores range from 0 to 8, with MMAS-8 score of <6 indicates low adherence, a	English	has been translated to Malay and has been validated	**forgetfulness**: point 1, 2, 8 (memory difficulties); point 4 (travelling), point 5, 7 (not able to make taking a drug part of a routine); **lack of knowledge**: point 6 (do not understand why to take the drug); **lack of motivation**: point 3 (high concern)	N/A

			score of 6 to <8 indicates medium adherence, and a score of 8 indicates high adherence				
Mohammad, Y.	MMAS-8	8	the first 7 of which are yes/no questions (no=1, yes=0), and the last of which is a 5 point Likert-scale rating (1=Never; 0.75=Rarely once; 0.5=In a while sometimes; 0.25=Usually; 0=All the time). MMAS-8 scores range from 0 to 8, with MMAS-8 score of <6 indicates low adherence, a score of 6 to <8 indicates medium adherence, and a score of 8 indicates high adherence	English	has been translated to Arabic and has been validated in pilot study	**forgetfulness**: point 1, 2, 8 (memory difficulties); point 4 (travelling), point 5, 7 (not able to make taking a drug part of a routine);<br>**lack of knowledge**: point 6 (do not understand why to take the drug);<br>**lack of motivation**: point 3 (high concern)	N/A
Garay-Sevilla, M.	no name	10	4 likert scale, Answers ranged from 1 to 4, with increasing scores for better adherence	N/A	has been developed	seven questions concerning about lifestyle and 3 question about continuity of intake of medication, self-decided changes of doses or its type.	N/A

Bruce, S.P	MMAS-4	4	dichotomous scoring yes or no questions. The number of patients who said ‘No’ to all four questions were considered adherent	English	has been translated to Ga, Twi and Ewe language	**forgetfulness**: point 1, 2, (memory difficulties)<br>**lack of knowledge**: point 3 (do not understand why to take the drug);<br>**lack of motivation**: point 4 (high concern)	N/A
Karakurt	no name	2	N/A	N/A	has been developed by the researcher special for this study	medication status and the factors affecting it (adherence, and reasons for non-adherence, listed viz. forgetfulness/aloneness/negligence, poverty, old age/inactivity, dislike of medicine, transportation difficulties, disturbance and failure to use multiple medicines together).	N/A
Alhaddad	MMAS-4	4	dichotomous scoring yes or no questions (yes=0, no=1). The number of patients who said ‘No’ to all four questions were considered adherent	English	N/A	**forgetfulness**: point 1, 2, (memory difficulties)<br>**lack of knowledge**: point 3 (do not understand why to take the drug);<br>**lack of motivation**: point 4 (high concern)	N/A

Kretchy, I.A.	MMAS-8	8	the first 7 of which are yes/no questions (no=1, yes=0), and the last of which is a 5 point Likert-scale rating (1=Never; 0.75=Rarely once; 0.5=In a while sometimes; 0.25=Usually; 0=All the time). MMAS-8 scores range from 0 to 8, with MMAS-8 score of <6 indicates low adherence, a score of 6 to <8 indicates medium adherence, and a score of 8 indicates high adherence	English	N/A	**forgetfulness**: point 1, 2, 8 (memory difficulties); point 4 (travelling), point 5, 7 (not able to make taking a drug part of a routine);<br>**lack of knowledge**: point 6 (do not understand why to take the drug);<br>**lack of motivation**: point 3 (high concern)	N/A
Mekonnen, H.S.	MMAS-8	8	the first 7 of which are yes/no questions (no=1, yes=0), and the last of which is a 5 point Likert-scale rating (1=Never; 0.75=Rarely once; 0.5=In a while sometimes; 0.25=Usually; 0=All the time). MMAS-8 scores range from 0 to 8, with MMAS-8 score of <6 indicates low adherence, a	English	has been translated to Amharic	**forgetfulness**: point 1, 2, 8 (memory difficulties); point 4 (travelling), point 5, 7 (not able to make taking a drug part of a routine);<br>**lack of knowledge**: point 6 (do not understand why to take the drug);<br>**lack of motivation**: point 3 (high concern)	N/A

			score of 6 to <8 indicates medium adherence, and a score of 8 indicates high adherence				
Mwangasha	MMAS-8	8	the first 7 of which are yes/no questions (no=1, yes=0), and the last of which is a 5 point Likert-scale rating (1=Never; 0.75=Rarely once; 0.5=In a while sometimes; 0.25=Usually; 0=All the time). MMAS-8 scores range from 0 to 8, with MMAS-8 score of <6 indicates low adherence, a score of 6 to <8 indicates medium adherence, and a score of 8 indicates high adherence	English	has been translated to Amharic	**forgetfulness**: point 1, 2, 8 (memory difficulties); point 4 (travelling), point 5, 7 (not able to make taking a drug part of a routine);<br>**lack of knowledge**: point 6 (do not understand why to take the drug);<br>**lack of motivation**: point 3 (high concern)	N/A

Balasubramaniam, S.	MMAS-8	8	the first 7 of which are yes/no questions (no=1, yes=0), and the last of which is a 5 point Likert-scale rating (1=Never; 0.75=Rarely once; 0.5=In a while sometimes; 0.25=Usually; 0=All the time). MMAS-8 scores range from 0 to 8, with MMAS-8 score of <6 indicates low adherence, a score of 6 to <8 indicates medium adherence, and a score of 8 indicates high adherence	English	has been translated to Malay and has been validated	**forgetfulness**: point 1, 2, 8 (memory difficulties); point 4 (travelling), point 5, 7 (not able to make taking a drug part of a routine); **lack of knowledge**: point 6 (do not understand why to take the drug); **lack of motivation**: point 3 (high concern)	Cronbach's alpha value was 0.675
Vancini-Campanharo	MMAS-4 and Brief Medication Questionnaire (BMQ)	4 and 9	MMAS-4: dichotomous scoring yes or no questions (yes=0, no=1). The number of patients who said 'No' to all four questions were considered adherent; BMQ: The presence of an affirmative response in each of the headings identifies a barrier to the medication adherence	English	N/A	**MMAS 4 = forgetfulness**: point 1, 2, (memory difficulties) **lack of knowledge**: point 3 (do not understand why to take the drug); **lack of motivation**: point 4 (high concern); BMQ= identify barrier to medication adherence: Regimen barrier, Belief barrier, and Recall barrier	N/A

Hassan, N.B	no name	10	5 likert scale Possible scores on the scale ranged from 1 to 5, with 1 indicating ‘never’ and 5 indicating ‘very frequent’. All negatively worded scores were reversed and all scores were converted to a 0 to 100 scale. Patients were categorized as ‘compliant’ if they had an individual score of 75% or greater	N/A	has been developed and has been validated in separated study	The questionnaire consisted of two domains: a drug-taking behaviour domain and drug-stopping behaviour domain	Cronbach’s alpha was 0.84
Nazir, S.U.R	MMAS-8	8	the first 7 of which are yes/no questions, and the last of which is a 5 point Likert-scale rating (1=Never; 0.75=Rarely once; 0.5=In a while sometimes; 0.25=Usually; 0=All the time). MMAS-8 scores range from 0 to 8, with MMAS-8 score of <6 indicates low adherence, a score of 6 to <8 indicates medium adherence, and a	English	has been translated in Urdu and has been validated	**forgetfulness**: point 1, 2, 8 (memory difficulties); point 4 (travelling), point 5, 7 (not able to make taking a drug part of a routine);<br>**lack of knowledge**: point 6 (do not understand why to take the drug);<br>**lack of motivation**: point 3 (high concern)	the Cronbach's alpha value was 0.701. spearman's rank coefficient was value was 0.8. correlation coefficient 0.3-0.4.

			score of 8 indicates high adherence				
Khadka	MMAS-4	4	dichotomous scoring yes or no questions. The number of patients who said ‘No’ to all four questions were considered adherent	English	N/A	**forgetfulness**: point 1, 2, (memory difficulties) **lack of knowledge**: point 3 (do not understand why to take the drug); **lack of motivation**: point 4 (high concern)	N/A
Kretchy	MARS-5	5	5-point Likert scale, where 5, “never”;4, “rarely”;3, “sometimes”;2, “often” and 1, “always”	English	has been validated	point 1: forgetfulness, point 2, 3, 4, 5 : lack of knowledge (one item that reflects unintentional non-adherence and four items that reflect intentional non-adherence)	Cronbach Alpha Coefficient was 0.6967.

Sartori	MMAS-4	4	dichotomous scoring yes or no questions. The number of patients who said ‘No’ to all four questions were considered adherent	English	has been validated and translated	**forgetfulness**: point 1, 2, (memory difficulties) **lack of knowledge**: point 3 (do not understand why to take the drug); **lack of motivation**: point 4 (high concern)	N/A
Owolabi	no name	7	dichotomous scoring yes or no questions.	N/A	has been developed	forgetfulness, carelessness, assumption of improved health or deterioration of health or users’ feelings	the alpha coefficient value was 0.68
Alfian, S.D	MARS-5	5	5-point Likert scale, where 5, “never”;4, “rarely”;3, “sometimes”;2, “often” and 1, “always”	English	has been translated to Indonesian and has been validated	point 1: forgetfulness, point 2, 3, 4, 5 : lack of knowledge (one item that reflects unintentional non-adherence and four items that reflect intentional non-adherence)	Cronbach Alpha Coefficient was 0,804

Cani	MMAS-4 and Medication Adherence Questionnaire (MAQ)	MGLS = 4; MAQ= 8	MGLS= dichotomous scoring yes or no questions (yes=0, no=1). The number of patients who said 'No' to all four questions were considered adherent; MAQ= The questions had Yes and No responses for each item. A mark of 1 was awarded for a "No" response and zero for a "Yes" response. The possible overall score for the 8-item medication adherence questionnaire ranged from zero to eight. Participants who had a total score of<6 were considered non-adherent and those whose scores were from 6–8 were categorised as adherent to medication.	English	has been translated to Brazilian and has been validated	**MGLS = forgetfulness**: point 1, 2, (memory difficulties)<br>**lack of knowledge**: point 3 (do not understand why to take the drug);<br>**lack of motivation**: point 4 (high concern); MAQ = forgetfulness: point 1, 8 (memory difficulties); point 4 (travelling), point 5, 7(not able to make as a routine activity)<br>lack of motivation: point 2, 6 (low necessity)<br>other drugs-related problem: point 3 (experience adverse event)	N/A

Khudair	Adherence to Refills and Medications Scale (ARMS)	12	4 Likert scale, with responses of “none”, “some”, “most”, or “all the time”, which are given values of 1–4. The range of possible scores is 12 to 48, with lower scores indicating better adherence. ARMS scores were categorized into: “adherent” with scores 12 or 13 and “non-adherent” with scores of 14 to 48	N/A	The questionnaire was translated to Arabic and has been validated	adherence to taking medications (eight items) and adherence to refilling prescriptions (four items) **forgetfulness**: point 1,3, 9 (memory difficult), point 8 (not able to make routine) **lack of motivation**: point 2, 6, 10 (low necessity) **other related problem**: point 4, 12 (run out medicine), point 5 (access of care), point 7 (related adverse effect), point 11 (financial issue)	Cronbach Alpha Coefficient was 0,814
Chung, W.W	Malaysian Medication Adherence Scale (MALMAS)	8	The first item of the MALMAS has five responses: (1) All the time, (2) Often, (3) Sometimes, (4) Rarely and (5) Never. The remaining seven items have a dichotomous response of “Yes” or “No”. MALMAS scores range from 0 to 8, medication adherence was classified into medication adherence (total score, 6–8) and nonadherence (total score > 6).	English	has been developed based MMAS-8 and has been validated	**forgetfulness**: point 1, 2, 8 (memory difficulties); point 4 (travelling), point 5, 7 (not able to make taking a drug part of a routine); **lack of knowledge**: point 6 (do not understand why to take the drug); **lack of motivation**: point 3 (high concern)	the Cronbach's alpha value was 0.565

Shen, Y.	no name	5	The first two items were dichotomous and coded as 0 and 1. The rest three items used Likert 5-point scale and coded as 0, 0.25, 0.50, 0.75, and 1, respectively. Higher scores represented higher MA.	N/A	has been developed based literature	**lack of motivation**: point 1, 2, 5 (low necessity)<br>**forgetfulness**: point 3,4 (memories difficulties)	the Cronbach's alpha value was 0.726
Delavar, F.	MMAS-8	8	the first 7 of which are yes/no questions, and the last of which is a 5 point Likert-scale rating (1=Never; 0.75=Rarely once; 0.5=In a while sometimes; 0.25=Usually; 0=All the time). MMAS-8 scores range from 0 to 8, with MMAS-8 score of <6 indicates low adherence, a score of 6 to <8 indicates medium adherence, and a score of 8 indicates high adherence	English	has been translated in Persian and has been validated	**forgetfulness**: point 1, 2, 8 (memory difficulties); point 4 (travelling), point 5, 7 (not able to make taking a drug part of a routine);<br>**lack of knowledge**: point 6 (do not understand why to take the drug);<br>**lack of motivation**: point 3 (high concern)	N/A

Barikani, A.	MARS-10	10	5-point Likert scale (never = 5, rarely = 4, sometimes = 3, often = 2, and always = 1). The score obtained from this questionnaire is divided into 10 and thus the scores fit between the ranges of1 to 5. Any score greater than 4.5 in-dicates good medication adherence and any score below 4.5 indicates poor medication adherence.	English	translated into Farsi and has been validated	**forgetfulness**: point 1 (memory difficulties); point 2, 6, 10 (not able to make taking a drug part of routine) **lack of knowledge:** point 3, 5, 8 (do not understand why to take the drug); **lack of motivation:** point 4,7, 9 (high necessity)	the Cronbach's alpha value was 0.73
Sheilini	MMAS-8	8	the first 7 of which are yes/no questions, and the last of which is a 5 point Likert-scale rating (1=Never; 0.75=Rarely once; 0.5=In a while sometimes; 0.25=Usually; 0=All the time). MMAS-8 scores range from 0 to 8, with MMAS-8 score of <6 indicates low adherence, a score of 6 to <8 indicates medium	English	has been translated in Kannada and has been validated	**forgetfulness**: point 1, 2, 8 (memory difficulties); point 4 (travelling), point 5, 7 (not able to make taking a drug part of a routine); **lack of knowledge**: point 6 (do not understand why to take the drug); **lack of motivation**: point 3 (high concern)	N/A

			adherence, and a score of 8 indicates high adherence				
Nazir, S.U.R	MMAS-8	8	the first 7 of which are yes/no questions, and the last of which is a 5 point Likert-scale rating (1=Never; 0.75=Rarely once; 0.5=In a while sometimes; 0.25=Usually; 0=All the time). MMAS-8 scores range from 0 to 8, with MMAS-8 score of <6 indicates low adherence, a score of 6 to <8 indicates medium adherence, and a score of 8 indicates high adherence	English	has been translated in Urdu and has been validated	**forgetfulness**: point 1, 2, 8 (memory difficulties); point 4 (travelling), point 5, 7 (not able to make taking a drug part of a routine);<br>**lack of knowledge**: point 6 (do not understand why to take the drug);<br>**lack of motivation**: point 3 (high concern)	the Cronbach's alpha value was 0.701. spearman's rank coefficient was value was 0.8. correlation coefficient 0.3-0.4.
Nesari, M.	no name	7	the questions related to their adherence to the therapeutic regimen in a five-point Likert scale.	N/A	has been developed	N/A	N/A

Bijam	no name	8	5-point Likert scale: No=1, Rarely=2, Occasionally=3, Usually=4, and Always=5.	Farsi	has been developed	N/A	N/A
Chow, E.P.	MMAS-8	8	the first 7 of which are yes/no questions, and the last of which is a 5 point Likert-scale rating (1=Never; 0.75=Rarely once; 0.5=In a while sometimes; 0.25=Usually; 0=All the time). MMAS-8 scores range from 0 to 8, with MMAS-8 score of <6 indicates low adherence, a score of 6 to <8 indicates medium adherence, and a score of 8 indicates high adherence	English	has been translated in Malaysia and has been validated	**forgetfulness**: point 1, 2, 8 (memory difficulties); point 4 (travelling), point 5, 7 (not able to make taking a drug part of a routine);<br>**lack of knowledge**: point 6 (do not understand why to take the drug);<br>**lack of motivation**: point 3 (high concern)	the Cronbach's alpha value was 0.675
Supachaipachni pong, S.	MMAS-8	8	the first 7 of which are yes/no questions, and the last of which is a 5 point Likert-scale rating (1=Never; 0.75=Rarely once; 0.5=In a while sometimes; 0.25=Usually; 0=All	English	has been translated in Thai and has been validated	**forgetfulness**: point 1, 2, 8 (memory difficulties); point 4 (travelling), point 5, 7 (not able to make taking a drug part of a routine);<br>**lack of knowledge**: point 6 (do not understand why to take the drug);<br>**lack of motivation**: point 3 (high concern)	the Cronbach's alpha value was 0.76

			the time). MMAS-8 scores range from 0 to 8, with MMAS-8 score of <6 indicates low adherence, a score of 6 to <8 indicates medium adherence, and a score of 8 indicates high adherence				
Shi	MMAS-8	8	the first 7 of which are yes/no questions, and the last of which is a 5 point Likert-scale rating (1=Never; 0.75=Rarely once; 0.5=In a while sometimes; 0.25=Usually; 0=All the time). MMAS-8 scores range from 0 to 8, with MMAS-8 score of <6 indicates low adherence, a score of 6 to <8 indicates medium adherence, and a score of 8 indicates high adherence	English	has been translated in Chinese and has been validated	**forgetfulness**: point 1, 2, 8 (memory difficulties); point 4 (travelling), point 5, 7 (not able to make taking a drug part of a routine);<br>**lack of knowledge**: point 6 (do not understand why to take the drug);<br>**lack of motivation**: point 3 (high concern)	the Cronbach's alpha value was 0.77

Ashur, S.T.	MMAS-8	8	the first 7 of which are yes/no questions, and the last of which is a 5 point Likert-scale rating (1=Never; 0.75=Rarely once; 0.5=In a while sometimes; 0.25=Usually; 0=All the time). MMAS-8 scores range from 0 to 8, with MMAS-8 score of <6 indicates low adherence, a score of 6 to <8 indicates medium adherence, and a score of 8 indicates high adherence	English	has been translated in Arabic and has been validated	**forgetfulness**: point 1, 2, 8 (memory difficulties); point 4 (travelling), point 5, 7 (not able to make taking a drug part of a routine);<br>**lack of knowledge**: point 6 (do not understand why to take the drug);<br>**lack of motivation**: point 3 (high concern)	the Cronbach's alpha value was 0.70
Mukora-Mutseyekwa, F.N.	MMAS-4	4	dichotomous scoring yes or no questions. . Patients who responded "No" to three or more of the four MMAS items (MMAS score of 0 or 1) were classified as adherent. Patients responding "No" to fewer than three of the four items (MMAS score 2, 3, or 4) were classified as non-adherent.	English	N/A	**forgetfulness**: point 1, 2, (memory difficulties)<br>**lack of knowledge**: point 3 (do not understand why to take the drug);<br>**lack of motivation**: point 4 (high concern)	N/A

Hou, Y.	MMAS-8	8	the first 7 of which are yes/no questions, and the last of which is a 5 point Likert-scale rating (1=Never; 0.75=Rarely once; 0.5=In a while sometimes; 0.25=Usually; 0=All the time). MMAS-8 scores range from 0 to 8, scores <6 and ≥6 reflected poor and good adherence, respectively	English	has been translated in Chinese and has been validated	**forgetfulness**: point 1, 2, 8 (memory difficulties); point 4 (travelling), point 5, 7 (not able to make taking a drug part of a routine); **lack of knowledge**: point 6 (do not understand why to take the drug); **lack of motivation**: point 3 (high concern)	the Cronbach's alpha value was 0.83
Mebrahtu	MARS-10	10	5-point Likert scale (never = 5, rarely = 4, sometimes = 3, often = 2, and always = 1). The score obtained from this questionnaire is divided into 10 and thus the scores fit between the ranges of1 to 5. Any score greater than 4.5 in-dicates good medication adherence and any score below 4.5 indicates poor medication adherence.	English	N/A	**forgetfulness**: point 1 (memory difficulties); point 2, 6, 10 (not able to make taking a drug part of routine) **lack of knowledge:** point 3, 5, 8 (do not understand why to take the drug); **lack of motivation:** point 4,7, 9 (high necessity)	N/A

Wang, W.	MMAS-4	4	dichotomous scoring yes or no questions. . Patients who responded "No" to three or more of the four MMAS items (MMAS score of 0 or 1) were classified as adherent. Patients responding "No" to fewer than three of the four items (MMAS score 2, 3, or 4) were classified as non-adherent.	English	has been translated to Chinese and has been validated	**forgetfulness**: point 1, 2, (memory difficulties) **lack of knowledge**: point 3 (do not understand why to take the drug); **lack of motivation**: point 4 (high concern)	the Cronbach's alpha value was 0.73
Mohamad	MMAS-8	8	the first 7 of which are yes/no questions, and the last of which is a 5 point Likert-scale rating (1=Never; 0.75=Rarely once; 0.5=In a while sometimes; 0.25=Usually; 0=All the time). MMAS-8 scores range from 0 to 8, scores <6 and $\geq$6 reflected poor and good adherence, respectively	English	has been translated in Arabic and has been validated	**forgetfulness**: point 1, 2, 8 (memory difficulties); point 4 (travelling), point 5, 7 (not able to make taking a drug part of a routine); **lack of knowledge**: point 6 (do not understand why to take the drug); **lack of motivation**: point 3 (high concern)	N/A

Wei.L	MARS-5	5	5-point Likert scale, where 5, “never”;4, “rarely”;3, “sometimes”;2, “often” and 1, “always”	English	has been translated to Chinese version	point 1: forgetfulness, point 2, 3, 4, 5 : lack of knowledge (one item that reflects unintentional non-adherence and four items that reflect intentional non-adherence)	N/A
Al-Daken	Hill-Bone medication adherence scale	14	4-point Likert scale (1= all of the time; 2 = most of the time; 3 = some of the time; 4=never). overall adherence score of 14 to 56, with higher scores indicating better adherence. A score ≥48 is considered as adherence and a score <48 is considered as non-adherence.	English	has been translated to Arabian and has been validated	**forgetfulness**: point 1, 8, 14 (memory difficulties); point 2, 6, 7, 9, 10 (not able to make taking a drug part of routine) **lack of knowledge:** point 11 (do not understand why to take the drug); point 13 (do not understand how to take the drug) **lifestyle**: point 3, 4, 5 **lack of motivation:** point 12 (low necessity)	Cronbach’s alpha 0.79
Gomes	MMAS-4	4	dichotomous scoring yes or no questions. . Patients who responded “No” to three or more of the four MMAS items (MMAS score of 0 or 1) were classified as adherent. Patients responding “No” to fewer than three of the four items (MMAS score 2, 3, or 4) were classified as non-adherent.	English	N/A	**forgetfulness**: point 1, 2, (memory difficulties) **lack of knowledge**: point 3 (do not understand why to take the drug); **lack of motivation**: point 4 (high concern)	N/A

Gu, L.	MMAS-8	8	the first 7 of which are yes/no questions, and the last of which is a 5 point Likert-scale rating (1=Never; 0.75=Rarely once; 0.5=In a while sometimes; 0.25=Usually; 0=All the time). MMAS-8 scores range from 0 to 8, scores <6 and ≥6 reflected poor and good adherence, respectively	English	has been translated in Chinese and has been validated	**forgetfulness**: point 1, 2, 8 (memory difficulties); point 4 (travelling), point 5, 7 (not able to make taking a drug part of a routine); **lack of knowledge**: point 6 (do not understand why to take the drug); **lack of motivation**: point 3 (high concern)	the Cronbach's alpha value was 0.84
Jarab	MMAS-4	4	dichotomous scoring yes or no questions (yes=1, no=0). . For the purpose of the present analysis, patients scoring zero were considered adherent and those scoring 1–4 were deemed non-adherent.	English	N/A	**forgetfulness**: point 1, 2, (memory difficulties) **lack of knowledge**: point 3 (do not understand why to take the drug); **lack of motivation**: point 4 (high concern)	N/A
Samu	MMAS-8	8	the first 7 of which are yes/no questions, and the last of which is a 5 point Likert-scale rating (1=Never; 0.75=Rarely once; 0.5=In a while	English	has been translated in Malayalam	**forgetfulness**: point 1, 2, 8 (memory difficulties); point 4 (travelling), point 5, 7 (not able to make taking a drug part of a routine); **lack of knowledge**: point 6 (do not understand why to take the drug);	N/A

			sometimes; 0.25=Usually; 0=All the time). MMAS-8 scores range from 0 to 8, scores <6 and >6 reflected poor and good adherence, respectively			**lack of motivation**: point 3 (high concern)	
Saleem	Drug Attitude Inventory (DAI)	10	scoring type was true (score=1) and false (score= -1) for each statement. The scale measured adherence from a maximum of 10 to a minimum of -10. Any negative score was rated as poor adherence, 0 - 5 as moderate adherence and 6 - 10 as good adherent.	English	has been translated to Urdu and has been validated	**lack of motivation** : point 1, 9 (high necessity), point 3 (low necessity)<br>**forgetfulness**: point 8 (make a daily routine)<br>**lack of knowledge**: point 6, 7, 10 (do not understand why to take drug)<br>**other drug-related problems:** point 2, 5 (experience adverse event)	Cronbach's alpha value was 0.65
Abdulsalim	Medication Adherence Questionnaire (MAQ)	8	The questions had Yes and No responses for each item. A mark of 1 was awarded for a "No" response and zero for a "Yes" response. The possible overall score for the 8-item medication adherence questionnaire ranged from zero to eight. Participants who had a total score of<6	English	has been translated to Dagbani and has been validated. The questionnaire was developed from previous instrument including MARS, Hill-	assessed deliberate and non-deliberate non-adherence including reasons for non-adherence<br>**forgetfulness**: point 1, 8 (memory difficulties); point 4 (travelling), point 5, 7(not able to make as a routine activity)<br>**lack of motivation**: point 2, 6 (low necessity)<br>**other drugs-related problem**: point 3 (experience adverse event)	The Cronbach alpha was 0.765

			were considered non-adherent and those whose scores were from 6–8 were categorised as adherent to medication.		Bone, and MMAS.		
Alfian, S.D	MARS-5	5	5-point Likert scale, where 5, “never”;4, “rarely”;3, “sometimes”;2, “often” and 1, “always”	English	has been translated to Indonesian and has been validated	point 1: forgetfulness, point 2, 3, 4, 5 : lack of knowledge (one item that reflects unintentional non-adherence and four items that reflect intentional non-adherence)	validity test using Pearson value correlation > 0,396 and Cronbach Alpha Coefficient 0,803
Akrom, A.	MMAS-8	8	the first 7 of which are yes/no questions, and the last of which is a 5 point Likert-scale rating	English	has been translated to Indonesian and has been validated	**forgetfulness**: point 1, 2, 8 (memory difficulties); point 4 (travelling), point 5, 7 (not able to make taking a drug part of a routine);<br>**lack of knowledge**: point 6 (do not understand why to take the drug);<br>**lack of motivation**: point 3 (high concern)	N/A

Al-kazaaz, H.K.	MMAS-8	8	the first 7 of which are yes/no questions, and the last of which is a 5 point Likert-scale rating (1=Never; 0.75=Rarely once; 0.5=In a while sometimes; 0.25=Usually; 0=All the time). MMAS-8 scores range from 0 to 8, with MMAS-8 score of <6 indicates low adherence, a score of 6 to <8 indicates medium adherence, and a score of 8 indicates high adherence	English	has been translated to Malaysian and has been validated	**forgetfulness**: point 1, 2, 8 (memory difficulties); point 4 (travelling), point 5, 7 (not able to make taking a drug part of a routine); **lack of knowledge**: point 6 (do not understand why to take the drug); **lack of motivation**: point 3 (high concern)	Cronbach's alpha value 0.675 with a significant test–retest reliability of 0.816.
Bizu, G.	MMAS-8	8	the first 7 of which are yes/no questions, and the last of which is a 5 point Likert-scale rating (1=Never; 0.75=Rarely once; 0.5=In a while sometimes; 0.25=Usually; 0=All the time). MMAS-8 scores range from 0 to 8, with MMAS-8 score of <6 indicates low adherence, a	English	has been translated to Amharic and has been validated	**forgetfulness**: point 1, 2, 8 (memory difficulties); point 4 (travelling), point 5, 7 (not able to make taking a drug part of a routine); **lack of knowledge**: point 6 (do not understand why to take the drug); **lack of motivation**: point 3 (high concern)	Cronbach's alpha value 0.803

			score of 6 to <8 indicates medium adherence, and a score of 8 indicates high adherence				
Teklay, G.	MMAS-4	4	dichotomous scoring yes or no questions. The number of patients who said ‘No’ to all four questions were considered adherent	English	N/A	**forgetfulness**: point 1, 2, (memory difficulties) **lack of knowledge**: point 3 (do not understand why to take the drug); **lack of motivation**: point 4 (high concern)	N/A
Ali, M. A	MMAS-8	8	the first 7 of which are yes/no questions, and the last of which is a 5 point Likert-scale rating (1=Never; 0.75=Rarely once; 0.5=In a while sometimes; 0.25=Usually; 0=All the time). MMAS-8 scores range from 0 to 8, with MMAS-8 score of <6 indicates low adherence, a score of 6 to <8 indicates medium	English	not translated and has been validated	**forgetfulness**: point 1, 2, 8 (memory difficulties); point 4 (travelling), point 5, 7 (not able to make taking a drug part of a routine); **lack of knowledge**: point 6 (do not understand why to take the drug); **lack of motivation**: point 3 (high concern)	N/A

			adherence, and a score of 8 indicates high adherence				
Ramli, A.	Medication Compliance Questionnaire (MCQ)	7	4-point Likert-type response format (none of the time = 4, some of the time = 3, most of the time = 2, and all the time = 1)	Malaysian	developed using he Hill-Bone Adherence to Blood Pressure Therapy Scale and the 8-item Morisky Medication Adherence Scale MMAS, and this questioinnaire has been validated	point 1, 2, 6: forgetfulness, point 3, 4: lack of knowledge point 5, 7: other drug-related problems (experience adverse event, access of care)	Cronbach’s alpha was 0.782 and the Kappa value of 0.796

Ungari, A.Q.	MMAS-4	4	dichotomous scoring yes or no questions. The number of patients who said 'No' to all four questions were considered adherent	English	N/A	point 1,2: forgetfuless<br>point 3: lack of knowledge<br>point 4: other drug-related problems (experience adverse drug event)	N/A
Saleem, F.	Drug Attitude Inventory	10	scoring type was true (score=1) and false (score= -1) for each statement. The scale measured adherence from a maximum of 10 to a minimum of - 10. Any negative score was rated as poor adherence, 0 - 5 as moderate adherence and 6 - 10 as good adherent.	English	has been translated to Urdu and has been validated	point 1, 3, 4, 9, 10: lack of motivation<br>point 2, 5: other drug-related problems (experience adverse event)<br>point 6, 7: lack of knowledge<br>point 8: forgetfulness	Cronbach's alpha value was 0.70
Saleem, F.	Drug Attitude Inventory	10	scoring type was true (score=1) and false (score= -1) for each statement. The scale measured adherence from a maximum of 10 to a minimum of - 10. Any negative score was rated as poor adherence, 0 - 5 as moderate adherence and 6 - 10 as good adherent.	English	has been translated to Urdu and has been validated	point 1, 3, 4, 9, 10: lack of motivation<br>point 2, 5: other drug-related problems (experience adverse event)<br>point 6, 7: lack of knowledge<br>point 8: forgetfulness	Cronbach's alpha value was 0.65

Nandini, H.C	MARS-5	5	5-point Likert scale, where 5, “never”;4, “rarely”;3, “sometimes”;2, “often” and 1, “always”	English	N/A	point 1: forgetfulness, point 2, 3, 4, 5 : lack of knowledge (one item that reflects unintentional non-adherence and four items that reflect intentional non-adherence)	N/A
Demoz, G.T	Brief Medication Questionnaire (BMQ)	9	Participants receive a score of 1 if their response indicates potential non-adherence and 0 if it indicates adherence	English	N/A	forgetfulness, lack of knowledge, lack of motivation, and other related drug problem	N/A
Kavitha, S	MMAS-8	8	the first 7 of which are yes/no questions, and the last of which is a 5 point Likert-scale rating (1=Never; 0.75=Rarely once; 0.5=In a while sometimes; 0.25=Usually; 0=All the time). MMAS-8 scores range from 0 to 8, with MMAS-8 score of <6 indicates low adherence, a score of 6 to <8	English	N/A	**forgetfulness**: point 1, 2, 8 (memory difficulties); point 4 (travelling), point 5, 7 (not able to make taking a drug part of a routine);<br>**lack of knowledge**: point 6 (do not understand why to take the drug);<br>**lack of motivation**: point 3 (high concern)	N/A

			indicates medium adherence, and a score of 8 indicates high adherence				
Abebaw, M.	MMAS-4	4	dichotomous scoring yes or no questions. The number of patients who said ‘No’ to all four questions were considered adherent	English	has been translated to Amharic	**forgetfulness**: point 1, 2, (memory difficulties) **lack of knowledge**: point 3 (do not understand why to take the drug); **lack of motivation**: point 4 (high concern)	N/A
Adisa, R.	MMAS-4	4	dichotomous scoring yes or no questions. The number of patients who said ‘No’ to all four questions were considered adherent	English	Has been validated (using original language)	**forgetfulness**: point 1, 2, (memory difficulties) **lack of knowledge**: point 3 (do not understand why to take the drug); **lack of motivation**: point 4 (high concern)	Cronbach’s alpha value was 0.61

Ogheonovo, A.	MMAS-8	8	the first 7 of which are yes/no questions, and the last of which is a 5 point Likert-scale rating (1=Never; 0.75=Rarely once; 0.5=In a while sometimes; 0.25=Usually; 0=All the time). MMAS-8 scores range from 0 to 8, with MMAS-8 score of <6 indicates low adherence, a score of 6 to <8 indicates medium adherence, and a score of 8 indicates high adherence	English	N/A	**forgetfulness**: point 1, 2, 8 (memory difficulties); point 4 (travelling), point 5, 7 (not able to make taking a drug part of a routine); **lack of knowledge**: point 6 (do not understand why to take the drug); **lack of motivation**: point 3 (high concern)	N/A
Divya, S.	MMAS-8	8	the first 7 of which are yes/no questions, and the last of which is a 5 point Likert-scale rating (1=Never; 0.75=Rarely once; 0.5=In a while sometimes; 0.25=Usually; 0=All the time). MMAS-8 scores range from 0 to 8, with MMAS-8 score of <6 indicates low adherence, a	English	N/A	**forgetfulness**: point 1, 2, 8 (memory difficulties); point 4 (travelling), point 5, 7 (not able to make taking a drug part of a routine); **lack of knowledge**: point 6 (do not understand why to take the drug); **lack of motivation**: point 3 (high concern)	N/A

			score of 6 to <8 indicates medium adherence, and a score of 8 indicates high adherence				
Gelaw, B.K	no name	6	4 likert scale which is 4: never; 3: rarely; 2: frequently; 1: daily	Portugal	Has been developed in Ethiopia and has been validated in pilot testing	Point 1: forgetfulness<br>Point 2: lack of knowledge<br>Point 3,6: other drug related problem (adverse event, traditional medication and religion belief)<br>Point 4, 5: lack of motivation (low necessity, high concern)	N/A
Gimenes, H.T	Measure Treatment Adherence (MTA)	7	6Likert-type scale for each question (always (1); almost always (2); frequently (3); sometimes (4); rarely (5); and never (6))	Portugal	has been translated to Brazilian and has been validated	Point 1,2.5,7: forgetfulness<br>Point 3: lack of knowledge<br>Point4: other drug-related problem (experience adverse event)	0.77 for sensitivity value and 0.73 specifity value
Heissam, K.	Measure Treatment Adherence (MTA)	7	6 Likert-type scale for each question (always (1); almost always (2); frequently (3); sometimes (4); rarely (5); and never (6))	Portugal	Has been validated (using original language)	Point 1,2.5,7: forgetfulness<br>Point 3: lack of knowledge<br>Point4: other drug-related problem (experience adverse event)	Cronbach alpha was 0.84

Istili, P.T.	Brief Medication Questionnaire (BMQ)	9	Participants receive a score of 1 if their response indicates potential non-adherence and 0 if it indicates adherence	English	N/A	forgetfulness, lack of knowledge, lack of motivation, and other related drug problem	N/A
Jemal, A.	MMAS-4	4	dichotomous scoring yes or no questions. The number of patients who said 'No' to all four questions were considered adherent	English	has been translated to Afan Oromo and Amharic	**forgetfulness**: point 1, 2, (memory difficulties)<br>**lack of knowledge**: point 3 (do not understand why to take the drug);<br>**lack of motivation**: point 4 (high concern)	N/A
Omar, M.S.	MMAS-4	4	dichotomous scoring yes or no questions. The number of patients who said 'No' to all four questions were considered adherent	English	N/A	**forgetfulness**: point 1, 2, (memory difficulties)<br>**lack of knowledge**: point 3 (do not understand why to take the drug);<br>**lack of motivation**: point 4 (high concern)	N/A
Iloh, G.U.P	Measure Treatment Adherence (MTA)	5	4 likert scale using an ordinal scoring system of 0-4 points as follows all-times=4 points, most-times=3 points, sometimes=2 points, rarely=1 point, never=0 point.	Portugal	has been developed by the author from several literature and has been validated in pilot testing	point 1-5: forgetfulness	N/A

Sajith, M.	MMAS-4	4	dichotomous scoring yes or no questions. The number of patients who said 'No' to all four questions were considered adherent	English	N/A	**forgetfulness**: point 1, 2, (memory difficulties)<br>**lack of knowledge**: point 3 (do not understand why to take the drug);<br>**lack of motivation**: point 4 (high concern)	N/A
Sankar, U.V	MMAS-8	8	the first 7 of which are yes/no questions, and the last of which is a 5 point Likert-scale rating (1=Never; 0.75=Rarely once; 0.5=In a while sometimes; 0.25=Usually; 0=All the time). MMAS-8 scores range from 0 to 8, with MMAS-8 score of <6 indicates low adherence, a score of 6 to <8 indicates medium adherence, and a score of 8 indicates high adherence	English	has been translated to Malayalam and has been done content validity and face validity	**forgetfulness**: point 1, 2, 8 (memory difficulties); point 4 (travelling), point 5, 7 (not able to make taking a drug part of a routine);<br>**lack of knowledge**: point 6 (do not understand why to take the drug);<br>**lack of motivation**: point 3 (high concern)	N/A
Shams, M.E.E.	Measure Treatment Adherence (MTA)	7	6 Likert-type scale for each question (always (1); almost always (2); frequently (3); sometimes (4); rarely (5); and never (6))	Portugal	N/A	Point 1,2.5,7: forgetfulness<br>Point 3: lack of knowledge<br>Point4: other drug-related problem (experience adverse event)	N/A

Tsehay, T.	MMAS-4	4	dichotomous scoring yes or no questions. The number of patients who said ‘No’ to all four questions were considered adherent	English	has been translated to Amharic and has been validated in pilot study	**forgetfulness**: point 1, 2, (memory difficulties)<br>**lack of knowledge**: point 3 (do not understand why to take the drug);<br>**lack of motivation**: point 4 (high concern)	N/A
MR, Almadhoun	MMAS-8	8	the first 7 of which are yes/no questions, and the last of which is a 5 point Likert-scale rating (1=Never; 0.75=Rarely once; 0.5=In a while sometimes; 0.25=Usually; 0=All the time). MMAS-8 scores range from 0 to 8, with MMAS-8 score of <6 indicates low adherence, a score of 6 to <8 indicates medium adherence, and a score of 8 indicates high adherence	English	Has been translated to Arab and has been validated	**forgetfulness**: point 1, 2, 8 (memory difficulties); point 4 (travelling), point 5, 7 (not able to make taking a drug part of a routine);<br>**lack of knowledge**: point 6 (do not understand why to take the drug);<br>**lack of motivation**: point 3 (high concern)	Cronbach alpha was 0.70 and reliability value was 0.65
Faria, H.T.G	Measure Treatment Adherence (MTA)	7	6 Likert-type scale for each question (always (1); almost always (2); frequently (3); sometimes (4); rarely (5); and never	Portugal	N/A	Point 1,2.5,7: forgetfulness<br>Point 3: lack of knowledge<br>Point4: other drug-related problem (experience adverse event)	N/A

			(6))				
Okoro, R.N.	no name	6	2-point Likert scale used, a mark of two (2) was awarded for Yes, and one (1) for No when they are expected; a mark of one (1) was for Yes, and two (2) for No when they are not expected; a mark of zero (0) was awarded for no response	N/A	has been developed by the author	Point1, 2: forgetfulness<br>point 3,4,6: other problems (access to care, experience adverse events)<br>Point 5: lack of knowledge	N/A
Olorunfemi, O.	MMAS-4	4	dichotomous scoring yes or no questions. The number of patients who said 'No' to all four questions were considered adherent	English	has been validated	**forgetfulness**: point 1, 2, (memory difficulties)<br>**lack of knowledge**: point 3 (do not understand why to take the drug);<br>**lack of motivation**: point 4 (high concern)	Cronbach alpha was 0.86
Son, N.E.	MMAS-6	6	The questions require Yes/No answers. "Yes" produces 1 point, "No" 0 point in questions 2 and 5, and "Yes" produces 0 point and "No" 1 point in other questions	English	has been translated to Turkish and has been validated	point 1,2, 6: forgetfulness<br>point 3, 5: lack of knowledge<br>point 4: other drug-related problems (experience adverse event)	N/A

Wu, P.	MMAS-8	8	the first 7 of which are yes/no questions, and the last of which is a 5 point Likert-scale rating (1=Never; 0.75=Rarely once; 0.5=In a while sometimes; 0.25=Usually; 0=All the time). MMAS-8 scores range from 0 to 8, with MMAS-8 score of <6 indicates low adherence, a score of 6 to <8 indicates medium adherence, and a score of 8 indicates high adherence	English	Has been translated to Chinese and has been validated	**forgetfulness**: point 1, 2, 8 (memory difficulties); point 4 (travelling), point 5, 7 (not able to make taking a drug part of a routine); **lack of knowledge**: point 6 (do not understand why to take the drug); **lack of motivation**: point 3 (high concern)	Cronbach alpha was 0.65 and intraclass correlation coefficient was 0.80
Aflakseir, A.	MARS-10	10	dichotomous (yes ⁄ no)	English	has been validated	**forgetfulness**: point 1 (memory difficulties); point 2, 6, 10 (not able to make taking a drug part of routine) **lack of knowledge:** point 3, 5, 8 (do not understand why to take the drug); **lack of motivation:** point 4,7, 9 (high necessity)	Cronbach's alpha was 0.79
Atulomah, N.O	no name	35	4-point scale which require respondents to answer: 0 = all of the time, 1 = most of the time, 2 = some of the time, 3 = none of	English and Yoruba	has been developed from the Health Belief Model and Hill-Born	N/A	Cronbach's alpha of 0.781

			the time		compliance scale. Has been validated		
Faisal, K.	Hill-Bone medication adherence scale	9	4-point Likert scale (1= all of the time; 2 = most of the time; 3 = some of the time; 4=never)	English	has been validated in pilot study, translated where necessary for patients who do not understand English	**forgetfulness**: point 1, 8, 14 (memory difficulties); point 2, 6, 7, 9, 10 (not able to make taking a drug part of routine)<br>**lack of knowledge:** point 11 (do not understand why to take the drug); point 13 (do not understand how to take the drug)<br><br>**lack of motivation:** point 12 (low necessity)	N/A
Inbaraj, L.R	MMAS-4	4	dichotomous scoring yes or no questions. The number of patients who said 'No' to all four questions were considered adherent	English	N/A	**forgetfulness**: point 1, 2, (memory difficulties)<br>**lack of knowledge**: point 3 (do not understand why to take the drug);<br>**lack of motivation**: point 4 (high concern)	N/A
Wong, M.C.S	MMAS-8	8	the first 7 of which are yes/no questions, and the last of which is a 5 point Likert-scale rating (1=Never; 0.75=Rarely once; 0.5=In a while sometimes; 0.25=Usually; 0=All the time). MMAS-8	English	Has been translated to Cantonese and has been validated	**forgetfulness**: point 1, 2, 8 (memory difficulties); point 4 (travelling), point 5, 7 (not able to make taking a drug part of a routine);<br>**lack of knowledge**: point 6 (do not understand why to take the drug);<br>**lack of motivation**: point 3 (high concern)	Cronbach alpha was 0.83

			scores range from 0 to 8, with MMAS-8 score of <6 indicates low adherence, a score of 6 to <8 indicates medium adherence, and a score of 8 indicates high adherence				
Kusa, W.	MMAS-8	8	the first 7 of which are yes/no questions, and the last of which is a 5 point Likert-scale rating (1=Never; 0.75=Rarely once; 0.5=In a while sometimes; 0.25=Usually; 0=All the time). MMAS-8 scores range from 0 to 8, with MMAS-8 score of <6 indicates low adherence, a score of 6 to <8 indicates medium adherence, and a score of 8 indicates high adherence	English	Has been translated to Afan Oromo and Amharic; has been validated	**forgetfulness**: point 1, 2, 8 (memory difficulties); point 4 (travelling), point 5, 7 (not able to make taking a drug part of a routine); **lack of knowledge**: point 6 (do not understand why to take the drug); **lack of motivation**: point 3 (high concern)	N/A
Bhattarai, B.	MMAS-4	4	dichotomous scoring yes or no questions. The number of patients who said 'No' to all four questions were	English	N/A	**forgetfulness**: point 1, 2, (memory difficulties) **lack of knowledge**: point 3 (do not understand why to take the drug); **lack of motivation**: point 4 (high	N/A

			considered adherent			concern)	
Boima, V.	MMAS-8	8	the first 7 of which are yes/no questions, and the last of question has dichotomous choice "never/rarely" versus "sometimes/usually/all the time". MMAS-8 scores range from 0 to 8, with MMAS-8 score of <6 indicates low adherence, a score of 6 to <8 indicates medium adherence, and a score of 8 indicates high adherence	English	has been validated in pilot study	**forgetfulness**: point 1, 2, 8 (memory difficulties); point 4 (travelling), point 5, 7 (not able to make taking a drug part of a routine);<br>**lack of knowledge**: point 6 (do not understand why to take the drug);<br>**lack of motivation**: point 3 (high concern)	N/A
Sontake, S.	MMAS-8	8	the first 7 of which are yes/no questions, and the last of which is a 5 point Likert-scale rating (1=Never; 0.75=Rarely once; 0.5=In a while sometimes; 0.25=Usually; 0=All the time). MMAS-8 scores range from 0	English	Has been translated to Afan Oromo and Amharic; has been validated	**forgetfulness**: point 1, 2, 8 (memory difficulties); point 4 (travelling), point 5, 7 (not able to make taking a drug part of a routine);<br>**lack of knowledge**: point 6 (do not understand why to take the drug);<br>**lack of motivation**: point 3 (high concern)	N/A

			to 8, with MMAS-8 score of <6 indicates low adherence, a score of 6 to <8 indicates medium adherence, and a score of 8 indicates high adherence				
Cai, Q.	MMAS-8	8	the first 7 of which are yes/no questions, and the last of which is a 5 point Likert-scale rating (1=Never; 0.75=Rarely once; 0.5=In a while sometimes; 0.25=Usually; 0=All the time). MMAS-8 scores range from 0 to 8, with MMAS-8 score of <6 indicates low adherence, a score of 6 to <8 indicates medium adherence, and a score of 8 indicates high adherence	English	Has been translated to Chinese and has been validated	**forgetfulness**: point 1, 2, 8 (memory difficulties); point 4 (travelling), point 5, 7 (not able to make taking a drug part of a routine); **lack of knowledge**: point 6 (do not understand why to take the drug); **lack of motivation**: point 3 (high concern)	Cronbach's alpha) for BMQ-Concern and BMQ-Necessity were 0.698 and 0.784, respectively and good test-retest reliability (intraclass correlation coefficient) was 0.729

Mwenee, M.D	Hill-Bone medication adherence scale	14	4-point Likert scale (1= all of the time; 2 = most of the time; 3 = some of the time; 4=never). score of less than 16, while non adherence was scored as greater than or equal to 16	English	has been validated and translated	**forgetfulness**: point 1, 8, 14 (memory difficulties); point 2, 6, 7, 9, 10 (not able to make taking a drug part of routine)<br>**lack of knowledge:** point 11 (do not understand why to take the drug); point 13 (do not understand how to take the drug)<br>**lifestyle**: point 3, 4, 5<br>**lack of motivation:** point 12 (low necessity)	Cronbach alpha was 0.79
Sharma, T.	MMAS-4	4	dichotomous scoring yes or no questions. The number of patients who said 'No' to all four questions were considered adherent	English	N/A	**forgetfulness**: point 1, 2, (memory difficulties)<br>**lack of knowledge**: point 3 (do not understand why to take the drug);<br>**lack of motivation**: point 4 (high concern)	N/A
Arias, M.F.	MMAS-8	8	the first 7 of which are yes/no questions, and the last of which is a 5 point Likert-scale rating (1=Never; 0.75=Rarely once; 0.5=In a while sometimes; 0.25=Usually; 0=All the time). MMAS-8 scores range from 0 to 8, with MMAS-8 score of <6 indicates low adherence, a score of 6 to <8 indicates medium	English	Has been translated to Spanish and has been validated	**forgetfulness**: point 1, 2, 8 (memory difficulties); point 4 (travelling), point 5, 7 (not able to make taking a drug part of a routine);<br>**lack of knowledge**: point 6 (do not understand why to take the drug);<br>**lack of motivation**: point 3 (high concern)	N/A

			adherence, and a score of 8 indicates high adherence				
Ledur, P.S.	MMAS-4	4	dichotomous scoring yes or no questions. The number of patients who said ‘No’ to all four questions were considered adherent	English	has been translated to Portuguese and has been validated	**forgetfulness**: point 1, 2, (memory difficulties)<br>**lack of knowledge**: point 3 (do not understand why to take the drug);<br>**lack of motivation**: point 4 (high concern)	N/A
Saarti, S.	MMAS-8	8	the first 7 of which are yes/no questions, and the last of which is a 5 point Likert-scale rating (1=Never; 0.75=Rarely once; 0.5=In a while sometimes; 0.25=Usually; 0=All the time). MMAS-8 scores range from 0 to 8, with MMAS-8 score of <6 indicates low adherence, a score of 6 to <8 indicates medium adherence, and a score of 8 indicates	English	N/A	**forgetfulness**: point 1, 2, 8 (memory difficulties); point 4 (travelling), point 5, 7 (not able to make taking a drug part of a routine);<br>**lack of knowledge**: point 6 (do not understand why to take the drug);<br>**lack of motivation**: point 3 (high concern)	N/A

			high adherence				
Asgari, M.R.	MMAS-8	8	the first 7 of which are yes/no questions, and the last of which is a 5 point Likert-scale rating (1=Never; 0.75=Rarely once; 0.5=In a while sometimes; 0.25=Usually; 0=All the time). MMAS-8 scores range from 0 to 8, with MMAS-8 score of <6 indicates low adherence, a score of 6 to <8 indicates medium adherence, and a score of 8 indicates high adherence	English	has been translated to Persian	**forgetfulness**: point 1, 2, 8 (memory difficulties); point 4 (travelling), point 5, 7 (not able to make taking a drug part of a routine); **lack of knowledge**: point 6 (do not understand why to take the drug); **lack of motivation**: point 3 (high concern)	Cron- bach's α, which reported a 0.90
Bhandari, B.	MMAS-4	4	dichotomous scoring yes or no questions. A total score of zero was considered as high adherence, a score of 1 or 2 as	English	N/A	**forgetfulness**: point 1, 2, (memory difficulties) **lack of knowledge**: point 3 (do not understand why to take the drug); **lack of motivation**: point 4 (high	N/A

			medium adherence, and a score of 3 or 4 as low adherence.			concern)	
Shams, N.	MMAS-8	8	the first 7 of which are yes/no questions, and the last of which is a 5 point Likert-scale rating (1=Never; 0.75=Rarely once; 0.5=In a while sometimes; 0.25=Usually; 0=All the time). MMAS-8 scores range from 0 to 8, with MMAS-8 score of <6 indicates low adherence, a score of 6 to <8 indicates medium adherence, and a score of 8 indicates high adherence	English	N/A	**forgetfulness**: point 1, 2, 8 (memory difficulties); point 4 (travelling), point 5, 7 (not able to make taking a drug part of a routine);<br>**lack of knowledge**: point 6 (do not understand why to take the drug);<br>**lack of motivation**: point 3 (high concern)	N/A
Teshome, D.F.	MMAS-4	4	dichotomous scoring yes or no questions. Ta score ≥3 defined as "good adherence".	English	has been translated to Amharic and has been validated	**forgetfulness**: point 1, 2, (memory difficulties)<br>**lack of knowledge**: point 3 (do not understand why to take the drug);<br>**lack of motivation**: point 4 (high concern)	N/A

Yue, Z.	MMAS-8	8	the first 7 of which are yes/no questions, and the last of which is a 5 point Likert-scale rating (1=Never; 0.75=Rarely once; 0.5=In a while sometimes; 0.25=Usually; 0=All the time). MMAS-8 scores range from 0 to 8, with MMAS-8 score of <6 indicates low adherence, a score of 6 to <8 indicates medium adherence, and a score of 8 indicates high adherence	English	N/A	**forgetfulness**: point 1, 2, 8 (memory difficulties); point 4 (travelling), point 5, 7 (not able to make taking a drug part of a routine);<br>**lack of knowledge**: point 6 (do not understand why to take the drug);<br>**lack of motivation**: point 3 (high concern)	N/A
Zhang, Y.	no name	4	4-likert scale (1) never complete, 2) sometimes complete, 3) usually complete, and 4) always complete), the participants who chose either the third or the fourth options as answers to all four questions were identified as adherent	Chinese	has been developed based on the extensive literature review of similar studies	**Dose frequency adherence:** point 1, 2,<br>**forgetfulness**: point 4	Cronbach alpha was 0.804

Supplementary 7. LMIC Related Medication Adherence Issues

Instrument name	Reason Non Adherence Assessed (WHO categories)															
	Patient Related Factors							Medication Related Factors			Provider and Healthcare System Related Factors			Societal Related Factors		
	1	2	3	4	5	6	7	8	9	10	11	12	13	14	15	16
ARMS		●			●		●		●			●	●			
BMQ		●		●	●			●					●			
DAI				●	●	●			●							
Hill-Bone medication adherence scale		●		●												
MALMAS		●	●	●		●										
MARS-10		●		●		●										
MARS-5		●		●												
MARS-A		●		●		●										
MASES	●	●	●	●	●	●	●	●	●			●		●		
MASES-SF	●	●	●	●	●	●			●					●		
MTA		●		●			●		●				●			
MAQ		●	●		●				●							
MCQ		●	●	●	●				●				●			
MMAS-4		●		●		●										
MMAS-6		●		●					●							
MMAS-7		●	●		●				●							

MMAS-8	●	●	●		●									
Self Care Inventory Diabetes (SCID)			●											
Self Reported Compliance Test	●										●		●	
TASHP	●		●		●			●						
No Name (1) Sevilla			●											
No Name (2) Karakurt	●					●	●				●		●	
No Name (3) Hassan	●		●			●		●		●	●			
No Name (4) Owolabi	●													
No Name (5) shen	●			●										
No Name (6) Nesari	●		●											
No Name (7) Bijam	●		●											
No Name (8) gelaw	●		●	●	●			●	●					●
No Name (9) okoro	●		●					●			●			
no name (10) atulomah	●		●									●		
no name (11) zhang	●		●											
no name (12) iloh	●		●											

(1) Being busy (2) memory difficulties (3) travelling (4) lack of knowledge (5) low necessity (6) high concern (7) run out of medicine (8) financial issues (9) polypharmacy (10) experience adverse effect (11) the use of traditional medicine (12) lack of communication with healthcare provider (13) access of care (14) dissatisfaction to the healthcare system (15) lack of support family (16) religion belief (17) stigma social

Supplementary 8. Self-Reported Instruments Included in the Scoping Review

Number	Instrument name	Adapted/Developed
1	ARMS	Adapted from HIC
2	BMQ	
3	DAI	
4	Hill-Bone medication adherence scale	
5	MALMAS	
6	MARS-10	
7	MARS-5	
8	MARS-A	
9	MASES	
10	MASES-SF	
11	MTA	
12	MAQ	
13	MCQ	
14	MMAS-4	
15	MMAS-6	
16	MMAS-7	
17	MMAS-8	
18	Self Care Inventory Diabetes (SCID)	
1	Self Reported Compliance Test	Developed by LMICs
2	TASHP	
3	No Name (1) Sevilla	
4	No Name (2) Karakurt	
5	No Name (3) Hassan	
6	No Name (4) Owolabi	
7	No Name (5) shen	
8	No Name (6) Nesari	
9	No Name (7) Bijam	
10	No Name (8) gelaw	
11	No Name (9) okoro	
12	no name (10) atulomah	
13	no name (11) zhang	
14	no name (12) iloh	

Supplementary 9. Quality Appraisal Studies Included

<u>Cohort Study Design: Newcastle Ottawa Scale for Cohort</u>

No	Title	Author (Year)	**SELECTION**														**COMPARABILITY**			**OUTCOME**											CONCL
			1				2			3					4		1			1					2		3				
			a	b	c	d	a	b	c	a	b	c	d	e	a	b	a	b	c	a	b	c	d	e	a	b	a	b	c	d	
1	Nonadherence to insulin therapy in low-income, type 2 diabetic patients.	Lerman, I., et.al.(2009)		*			*				*					√		*				√			*		*				Good
2	Treatment adherence and quality of life in patients on antihypertensive medications in a Middle Eastern population: adherence.	Alhaddad, et.al.(2016)		*			*				*					√	*	*				√			*		*				Good

<u>Selection</u>

1) Representativeness of the exposed cohort
    a) Truly representative (one star)
    b) Somewhat representative (one star)
    c) Selected group
    d) No description of the derivation of the cohort

2) Selection of the non-exposed cohort
    a) Drawn from the same community as the exposed cohort (one star)

b) Drawn from a different source
c) No description of the derivation of the non exposed cohort

3) Ascertainment of exposure
   a) Secure record (e.g., surgical record) (one star)
   b) Structured interview (one star)
   c) Written self report
   d) No description
   e) Other

4) Demonstration that outcome of interest was not present at start of study
   a) Yes (one star)
   b) No

Comparability

1) Comparability of cohorts on the basis of the design or analysis controlled for confounders
   a) The study controls for age, sex and marital status (one star)
   b) Study controls for other factors (list) ___________________________________ (one star)
   c) Cohorts are not comparable on the basis of the design or analysis controlled for confounders

Outcome

1) Assessment of outcome
   a) Independent blind assessment (one star)
   b) Record linkage (one star)
   c) Self report
   d) No description
   e) Other

2) Was follow-up long enough for outcomes to occur
   a) Yes (one star)
   b) No
   Indicate the median duration of follow-up and a brief rationale for the assessment above:____________________

3) Adequacy of follow-up of cohorts
   a) Complete follow up- all subject accounted for (one star)
   b) Subjects lost to follow up unlikely to introduce bias- number lost less than or equal to 20% or description of those lost suggested no different from those followed. (one star)
   c) Follow up rate less than 80% and no description of those lost
   d) No statement

Thresholds for converting the Newcastle-Ottawa scales to AHRQ standards (good, fair, and poor):

Good quality: 3 or 4 stars in selection domain AND 1 or 2 stars in comparability domain AND 2 or 3 stars in outcome/exposure domain

Fair quality: 2 stars in selection domain AND 1 or 2 stars in comparability domain AND 2 or 3 stars in outcome/exposure domain

Poor quality: 0 or 1 star in selection domain OR 0 stars in comparability domain OR 0 or 1 stars in outcome/exposure domain

Quasi Study Design: The Joanna Briggs Institute Critical Appraisal Tools for Quasi Experimental Studies

No	Title	Author (Year)	QUASI SCORING									%	CONCL
			1	2	3	4	5	6	7	8	9		
1	The feasibility and effect of a nurse-led, patient-centered asthma education program in a Nigerian context.	Ozoh, O.B., et.al.(2021)	Y	Y	Y	Y	Y	U	Y	Y	Y	88,9	High
2	A comparison between multimedia and traditional education in encouraging adherence to treatment regimen in patients with hypertension	Bijam, et.al.(2020)	Y	Y	Y	Y	Y	U	Y	Y	Y	88,9	High
3	An Education Intervention for Medication Adherence in Uncontrolled Diabetes in Thailand	Supachaipachnipong, S., et.al.(2018)	Y	Y	Y	Y	Y	U	Y	Y	Y	88,9	High

Y= Yes; N= No; U: Unclear

1. Is it clear in the study what is the “cause” and what is the “effect” (i.e. there is no confusion about which variable comes first)?
2. Were the participants included in any comparison similar?
3. Were the participants included in any comparison receiving similar treatment/care, other than the exposure or intervention of interest?
4. Was there a control group?
5. Were there multiple measurement of the outcome both pre and post intervention/exposure?
6. Was follow up complete and I f not, were differences between groups in terms of their follow up adequately described and analyzed?
7. Were the outcomes of participants included in any comparisons measured in the same way?
8. Were outcomes measured in a reliable way?
9. Was appropriate statistical analysis used?

Studies were categorized as “high quality” if they met at least 75% of these standards, “moderate” if they met between 50% and 75% of relevant standards, and “low” if less than 50%

<u>Mixed Method Study Design: Mixed Methods Appraisal Tool</u>

No	Title	Author (Year)	SCORING					CONCL
			1	2	3	4	5	
1	Self-reported medication adherence among patients with diabetes or hypertension, Médecins Sans Frontières Shatila refugee camp, Beirut, Lebanon: A mixed-methods study.	Mohamad, et.al.(2021)	Y	Y	Y	Y	Y	GOOD

Y= Yes; N= No; CT: Can't Tell

1. Is there an adequate rationale for using a mixed methods design to address the research question?
2. Are the different components of the study effectively integrated to answer the research question?
3. Are the outputs of the integration of qualitative and quantitative components adequately interpreted?
4. Are divergences and inconsistencies between quantitative and qualitative results adequately addressed?
5. Do the different components of the study adhere to the quality criteria of each tradition of the methods involved?

Randomized Controlled Trial Study Design: The Joanna Briggs Institute Critical Appraisal Tools for Randomized Controlled Trial

No	Title	Author (Year)	SCORING RCT													%	CONCL
			1	2	3	4	5	6	7	8	9	10	11	12	13		
1	The impact of pharmacist-led medication therapy management on medication adherence in patients with type 2 diabetes mellitus: a randomized controlled study.	Erku, D.A., et.al.(2017)	U	U	Y	Y	N	N	Y	Y	Y	Y	Y	Y	Y	69,2	Moderate
2	Educational Intervention Using WhatsApp on Medication Adherence in Hypertension and Diabetes Patients: a Randomized Clinical Trial	Sartori, et.al.(2020)	Y	Y	Y	Y	N	Y	Y	Y	Y	Y	Y	Y	Y	92,3	High
3	Impact of mobile phone text messaging intervention on adherence among patients with diabetes in a rural setting: a randomized controlled trial	Owolabi, et.al.(2020)	Y	Y	Y	Y	N	Y	Y	Y	Y	Y	Y	Y	Y	92,3	High
4	Effectiveness of a targeted and tailored pharmacist-led intervention to improve adherence to antihypertensive drugs among patients with type 2 diabetes in Indonesia: a cluster randomised controlled trial	Alfian, S.D, et.al.(2021)	Y	Y	Y	Y	N	Y	Y	Y	Y	Y	Y	Y	Y	92,3	High
5	Improvement in medication adherence and self-management of diabetes with a clinical pharmacy program: a randomized controlled trial in patients with type 2 diabetes undergoing insulin therapy at a teaching hospital	Cani, et.al.(2015)	Y	Y	Y	Y	N	Y	Y	Y	Y	Y	Y	Y	Y	92,3	High

6	Effects of a pharmaceutical care model on medication adherence and glycemic control of people with type 2 diabetes	Chung, W.W, et.al.(2014)	Y	Y	Y	Y	N	Y	Y	N	Y	Y	Y	Y	Y	84,6	High
7	Effectiveness of low-cost reminder package combined with case-based health education to improve hypertensive patients' medication adherence: a clustered randomized controlled trial	Shen, Y., et.al.(2019)	Y	Y	Y	N	N	Y	Y	Y	Y	Y	Y	Y	Y	84,6	High
8	The effects of self-management education tailored to health literacy on medication adherence and blood pressure control among elderly people with primary hypertension: a randomized controlled trial	Delavar, F., et.al.(2020)	Y	Y	Y	Y	N	Y	Y	Y	Y	Y	Y	Y	Y	92,3	High
9	The Impact of Motivational Interview on Self-Efficacy, Beliefs About Medicines and Medication Adherence Among Adolescents with Asthma: a Randomized Controlled Trial	Barikani, A., et.al.(2021)	Y	Y	Y	N	N	Y	Y	Y	Y	Y	Y	Y	Y	84,6	High
10	Impact of multimodal interventions on medication nonadherence among elderly hypertensives: a randomized controlled study	Sheilini, et.al.(2019)	Y	Y	Y	N	N	Y	Y	Y	Y	Y	Y	Y	Y	84,6	High
11	Medication Management Program: adherence, Disease-related Knowledge, Health-related Quality of Life, and Glycemic Control for Type 2 Diabetes Mellitus	Nazir, S.U.R, et.al.(2020)	Y	Y	Y	N	N	Y	Y	Y	Y	Y	Y	Y	Y	84,6	High

12	Effect of telephone follow-up on adherence to a diabetes therapeutic regimen	Nesari, M., et.al.(2010)	Y	U	Y	N	N	Y	Y	Y	Y	Y	Y	Y	Y	76,9	High
13	Effects of pharmacist-led patient education on diabetes-related knowledge and medication adherence: a home-based study	Chow, E.P., et.al.(2015)	Y	Y	Y	N	N	Y	Y	Y	Y	Y	Y	Y	Y	84,6	High
14	Pharmacist intervention in improving hypertension-related knowledge, treatment medication adherence and health-related quality of life: a non-clinical randomized controlled trial	Saleem, et.al.(2015)	Y	U	Y	N	N	N	Y	Y	Y	Y	Y	Y	Y	69,2	Moderate
15	Structured pharmacist-led intervention programme to improve medication adherence in COPD patients: a randomized controlled study	Abdulsalim, et.al.(2018)	Y	U	Y	N	N	N	Y	Y	Y	Y	Y	Y	Y	69,2	Moderate

Y= Yes; N= No; CT: Can't Tell

1. Was true randomization used for assignment of participants to treatment groups?
2. Was allocation to treatment groups concealed?
3. Were treatment groups similar at the baseline?
4. Were participants blind to treatment assignment?
5. Were those delivering treatment blind to treatment assignment?
6. Were outcomes assessors blind to treatment assignment?
7. Were treatment groups treated identically other than the intervention of interest?
8. Was follow up complete and if not, were differences between groups in term of their follow up adequately described and analyzed?
9. Were participants analyzed in the groups to which they were randomized?
10. Were outcomes measured in the same way for treatment groups?
11. Were outcomes measured in a reliable way?
12. Was appropriate statistical analysis used?

13. Was the trial design appropriate, and any deviations from the standard RCT design (individual randomization, parallel group) accounted for in the conduct and analysis of the trial?

Studies were categorized as “high quality” if they met at least 75% of these standards, “moderate” if they met between 50% and 75% of relevant standards, and “low” if less than 50%

<u>Cross Sectional Study Design: Newcastle Ottawa Scale for Cross Sectional</u>

No	Title	Author (Year)	SELECTION												COMPARABILITY		OUTCOME						TOTAL	CONCL.
			1				2		3			4			1		1				2			
			a	b	c	d	a	b	a	b	c	a	b	c	a	b	a	b	c	d	a	b		
1	Relationship of treatment satisfaction to medication adherence: findings from a cross-sectional survey among hypertensive patients in Palestine.	Zyoud, S.H, et.al.(2013)		*			*		*			**			*				*		*		8	Good
2	Influence of patients' disease knowledge and beliefs about medicines on medication adherence: findings from a cross-sectional survey among patients with type 2 diabetes mellitus in Palestine.	Sweileh, W. M, et.al.(2014)		*			*				√		*		*				*		*		6	Moderate

3	Necessity and concerns about lipid-lowering medical treatments and risk factors for non-adherence: A cross-sectional study in Palestine.	Shakarneh, et.al.(2020)		*	*		*			*	*	*	*	7	Good
4	Adherence to medications and associated factors: A cross-sectional study among Palestinian hypertensive patients.	Al-Ramahi, R., et.al.(2015)	*		*			√		*		*	*	5	Moderate
5	Beliefs About Medicine and Glycemic Control Among Type 2 Diabetes Patients: A Cross-Sectional Study in West Bank, Palestine.	Khdour, M.R., et.al.(2020)		*		√		√	**		*	*	*	6	Moderate
6	Medications Adherence and Associated Factors among Patients with Type 2 Diabetes Mellitus in the Gaza Strip, Palestine.	Elsous, A. , et.al.(2017)	*		*		*		**		*	*	*	8	Good

7	Effect of patients beliefs about medications on adherence to drugs in diabetic patients attending family medicine outpatient clinic in Ismailia, Egypt.	Salama, H.M, et.al.(2020)		*	*		√	**		*		*	*	7	Good
8	Barriers to diabetes medication adherence in North West Ethiopia.	Abebe, S.M. , et.al.(2014)	*		*	*			*	*		*	*	7	Good
9	Hypertension-related knowledge, practice and drug adherence among inpatients of a hospital in samarkand, uzbekistan.	Malik, A., et.al.(2014)	*		*	*			*		*	*	*	7	Good
10	Adherence and satisfaction with oral hypoglycemic medications: a pilot study in Palestine.	Jamous, R.M , et.al.(2011)		*	*	*			*	*		*	*	7	Good
11	Adherence to drug therapy among hypertensive patients attending two district hospitals in Ghana.	Sarkodie, E., et.al.(2020)		*	*		√		*	*		*	*	6	Moderate

12	Does adherence to the therapeutic regimen associate with health related quality of life: Findings from an observational study of type 2 diabetes mellitus patients in Pakistan.	Nazir, S.U.R, et.al.(2017)		√	*				√	**			*	*	5	Moderate
13	Effect of social support on the treatment adherence of hypertension patients.	Turan, G.B, et.al.(2019)		√		√			√	**		*	*	*	5	Moderate
14	Impact of pharmaceutical care in the improvement of medication adherence and quality of life for COPD patients in Vietnam.	Nguyen, T., et.al.(2019)	*			√		√			*	*	*	*	5	Moderate
15	Medication adherence in adult Chinese patients with asthma: role of illness perceptions and medication beliefs.	Cai, Q., et.al.(2021)	*			√			√	**		*	*	*	6	Moderate
16	Determinants of blood pressure control amongst hypertensive patients in Northwest	Teshome, D.F., et.al.(2018)		√	*		*			**		*	*	*	7	Good

	Ethiopia.												
17	Determinants of hypertension treatment adherence among a Chinese population using the therapeutic adherence scale for hypertensive patients.	Pan, J., et.al.(2019)	*	*	*		**		*	*	*	8	Good
18	Treatment Satisfaction and Medication Adherence Among Hypertensive Patients Seeking Care in Selected Hospitals in Ibadan, Nigeria.	Ajayi, D.T, et.al.(2018)	*	*		√	**		*	*	*	7	Good
19	Perceived Illness and Treatment Adherence to Hypertension Among Patients Attending a Tertiary Hospital in Kathmandu, Nepal.	Shakya, et.al.(2020)	*	*		√	**			*	*	6	Moderate
20	Blood Pressure Control, Accessibility, and Adherence to Antihypertensive Medications:	Adomako, N.O, et.al.(2021)	*	*		√		*		*	*	5	Moderate

	Patients Seeking Care in Two Hospitals in the Ashanti Region of Ghana.										
21	The Effect of Social Support on Treatment Adherence in Hypertension in China.	Pan, J., et.al.(2021)	*	*	√	**	*	*	*	7	Good
22	Adherence to antidiabetic medication and factors associated with non-adherence among patients with type-2 diabetes mellitus in two regional hospitals in Cameroon.	Aminde, L.N., et.al.(2019)	*	√	√	*		*	*	4	Unsatisfactory
23	Medication adherence in patients with type 2 diabetes mellitus treated at primary health clinics in Malaysia.	Ahmad, N.S., et.al.(2013)	*	*	√	**	*	*	*	7	Good
24	Antihypertensive Medications Adherence Among Nigerian Hypertensive Subjects in a Specialist Clinic	Akintunde, A.A., et.al.(2015)	*	√	√	*	*	*	√	4	Unsatisfactory

	Compared to a General Outpatient Clinic.													
25	Knowledge of Hypertension and Compliance with Therapy Among Hypertensive Patients in the Bamenda Health District of Cameroon: A Cross-sectional Study.	Akoko, B.M., et.al.(2017)	*	*			√		*	*	*	*	6	Moderate
26	Discordance between medication adherence and blood pressure control in primary care clinics in Negeri Sembilan, Malaysia: The problem of therapeutic inertia.	Chan, C.W., et.al.(2015)	*		√	*		**		*	*	*	7	Good
27	Predictors of non-adherence to antihypertensive medication in Kinshasa, Democratic Republic of Congo: a cross-sectional study.	Lulebo, et.al.(2015)	*	*			√		*	*	*	*	6	Moderate

28	Assessment of medication adherence and knowledge regarding the disease among ambulatory patients with diabetes mellitus in Karachi, Pakistan.	Abbas, et.al.(2015)		*			√		√	*		*		√	3	Unsatisfactory
29	Self Care and Medication Adherence among Type 2 Diabetics in Puducherry, Southern India: A Hospital Based Study.	Arulmozhi, S., et.al.(2014)			√		√		√	*		*	*		3	Unsatisfactory
30	Adherence to antihypertensive treatment and associated factors among patients on follow up at University of Gondar Hospital, Northwest Ethiopia.	Ambaw, et.al.(2012)	*			*		*		*	*	*	*		7	Good
31	Medication adherence and factors associated with poor adherence among type 2 diabetes mellitus patients on follow-up at Kenyatta National Hospital, Kenya.	Waari, G., et.al.(2018)	*			*			√	*	*	*	*		6	Moderate

32	Diabetic health literacy and its association with glycemic control among adult patients with type 2 diabetes mellitus attending the outpatient clinic of a university hospital in Ethiopia.	Tefera, Y.G, et.al.(2020)	*	*		√	*	*	*	*	6	Moderate
33	Prevalence of medication adherence and its associated factors among patients with noncommunicable disease in rural Puducherry, South India – A facility-based cross-sectional study.	Yuvaraj, K., et.al.(2019)	√	*	*		*		*	*	5	Moderate
34	Descriptive Cross-Sectional Study on Knowledge, Awareness, and Adherence to Medication among Hypertensive Patients at a Tertiary Care Centre in Colombo District, Sri Lanka.	Pirasath, S., et.al.(2020)	*	*	*	**		*	*	*	8	Good

35	Prevalence and risk factors of cognitive dysfunction in patients with type 2 diabetes mellitus receiving care in a reference hospital in Cameroon: a cross-sectional study.	Abba, Z.I., et.al.(2018)	√	√	*	*	*	*	*	5	Moderate
36	Adherence to anti-hypertensive medication and contributing factors among non-comorbid hypertensive patients in two hospitals of jimma town, south west ethiopia.	Dego, T.R., et.al.(2016)	√	√	√	*		*	*	3	Unsatisfactory
37	Magnitude and associated factors of poor medication adherence among diabetic and hypertensive patients visiting public health facilities in Ethiopia during the COVID-19 pandemic.	Shimels, T., et.al.(2021)	*	*	√	*	*	*	*	6	Moderate
38	Adherence to treatment among hypertensives of rural Kerala, India.	Balasubramanian, A., et.al.(2018)	√	*	√	*	*	*	*	5	Moderate

39	Non-Adherence to Prescribed Antihypertensives in Primary, Secondary and Tertiary Healthcare Settings in Islamabad, Pakistan: A Cross-Sectional Study.	Mahmood, S., et.al.(2020)	√	√	√	*	*		*	*	4	Unsatisfactory
40	Evaluation of Medication Adherence and Associated Factors in Hypertensive Patients in Bangalore, India; A Cross-Sectional Study.	Chaudhary, A.P., et.al.(2021)	*	√	√	*	*		*	*	5	Moderate
41	Awareness, medication adherence, and diet pattern among hypertensive patients attending teaching institution in western Rajasthan, India.	Mathur, D., et.al.(2020)	√	*	√	*	*		*	*	5	Moderate
42	Nonadherence and factors affecting adherence of diabetic patients to anti-diabetic medication in Assela General Hospital, Oromia Region, Ethiopia	Kassahun, A., et.al.(2016)	√	*	√	*	*		*	*	5	Moderate

43	Patients' socio-demographic variables and adherence to antihypertensive therapy in Southern Senatorial District of Cross River State, Nigeria.	Okeke, H.C., et.al.(2019)	*	*	√	*		*	*	5	Moderate
44	Medication adherence and self-care behaviours among patients with type 2 diabetes mellitus in Ghana.	Afaya, R.A., et.al.(2020)	*	*	√	*	*	*	*	6	Moderate
45	Treatment satisfaction, medication adherence, and blood pressure control among adult Nigerians with essential hypertension.	Iloh, G.U.P, et.al.(2017)	*	*	√	*	*	*	*	6	Moderate
46	Adherence to antihypertensive medications in rural Lao PDR: a prospective observational study.	Takahashi, E., et.al.(2021)	√	*	√	*	*	*	*	5	Moderate

47	Hypertension-related knowledge, medication adherence and health-related quality of life (HRQoL) among hypertensive patients in Islamabad, Pakistan.	Amer, M., et.al.(2019)			√	*	√		*	*	*	*	5	Moderate
48	Factors associated with medication adherence among people with diabetes mellitus in poor urban areas of Cambodia: A cross-sectional study.	Nonogaki, A., et.al.(2019)		*		*	√	**		*	*	*	7	Good
49	Adherence to antihypertensive therapy and its determinants among patients attending primary care hospitals of Kashmir, India.	Raja, W., et.al.(2021)		*		*	√		*	*	*	*	6	Moderate
50	Medication adherence and its associated factors among diabetic patients at Zewditu Memorial Hospital, Addis Ababa, Ethiopia.	Ali, M., et.al.(2017)	*			*	√		*	*	*	*	6	Moderate

51	Nonadherence to Drugs among the Hypertensive Patients in Outpatient Department of a Secondary Hospital of Bangladesh.	Ullah, et.al.(2019)	√	√	√	*	*	*	*	4	Unsatisfactory
52	Adherence to Antihypertensive Treatment and Associated Factors in Central Ethiopia.	G/Tsadik, D., et.al.(2020)	*	*	*	*	*	*	*	7	Good
53	Medication Adherence among Hypertensive Patients Attending a Tertiary Care Hospital in Nepal.	Roka, et.al.(2020)	*	√	√	*	*	*	*	5	Moderate
54	Patient satisfaction and medication adherence assessment amongst patients at the diabetes medication therapy adherence clinic.	Bakar, Z.A, et.al.(2016)	*	√	√	**		*	*	5	Moderate
55	Determinants of antihypertensive adherence among patients in Beijing: Application of the health belief model.	Yang, S., et.al.(2016)	*	*	√	**		*	*	6	Moderate

56	Impact of adverse drug events and treatment satisfaction on patient adherence with antihypertensive medication - a study in ambulatory patients.	Berhe, D.F., et.al.(2017)		*	*		*		**		*	*	*	8	Good
57	Adherence to Oral Hypoglycemic Drugs among Type 2 Diabetic Patients in a Resource-Poor Setting	Sefah, I.A., et.al.(2020)		*	*		*			*		*	*	6	Moderate
58	Medication non-adherence in patients with type 2 diabetes mellitus with full access to medicines.	Saraiva, E.M.S, et.al.(2020)		*		√	*		**		*	*	*	7	Good
59	Insulin Adherence in Type 2 Diabetes in Mexico: Behaviors and Barriers.	Bermeo-Cabrera, J., et.al.(2018)		*	*			√		*	*	*	*	6	Moderate
60	Medication non-adherence and associated factors among diabetes patients in Felege Hiwot Referral Hospital, Bahir Dar city administration, Northwest Ethiopia.	Abate, T.W. , et.al.(2019)	*		*		*			*	*	*	*	7	Good

61	Medication adherence and patient satisfaction among hypertensive patients attending outpatient clinic in Lagos University Teaching Hospital, Nigeria.	Oluwole, E.O, et.al.(2019)	*			*			√	**		*	*	*	7	Good
62	Adherence to insulin therapy and associated factors among patients with diabetes mellitus in public hospitals of Central Zone of Tigray, Ethiopia, 2018: a cross-sectional study.	Mariye, T., et.al.(2019)		*		*		*			*	*	*	*	7	Good
63	Medication adherence in type 2 diabetes patients in Nigeria	Jackson, I., et.al.(2015)			√		√	*			*	*	*	*	5	Moderate
64	Adherence to asthma treatment and their association with asthma control in children.	Basharat, et.al.(2018)			√	*			√		*	*	*	*	5	Moderate
65	Medication adherence and direct treatment cost among diabetes patients attending a tertiary healthcare facility in Ogbomosho,	Fadare, J., et.al.(2015)		*		*			√		*	*	*	*	6	Moderate

	Nigeria.														
66	Exploring variables associated with medication non-adherence in patients with type 2 diabetes mellitus.	Al-Qerem, W., et.al.(2021)		*	*		*		**		*	*	*	8	Good
67	Assessment of medication adherence among type 2 diabetic patients in Quetta city, Pakistan.	Iqbal, et.al.(2017)		*	*		*		**		*	*	*	8	Good
68	Ego defense mechanisms, medication adherence and self-management of the patients with type 2 diabetes.	Akram, F., et.al.(2021)	*		*			√	**		*	*	*	7	Good
69	Patient Education and Medication Adherence among Hypertensives in a Tertiary Hospital, South Western Nigeria.	Ayodapo, A.O., et.al.(2020)	*		*			√		*	*	*	*	6	Moderate
70	Factors associated with adherence to anti-hypertensive treatment in	Hashmi, et.al.(2007)		*		√	*			*	*	*	*	6	Moderate

	Pakistan.												
71	The effect of loneliness and perceived social support on medication adherence self-efficacy in hypertensive patients: An example of Turkey.	Asilar, H., et.al.(2020)	*	*		√	**			*	*	6	Moderate
72	Factors associated with glycemic control among adult patients with type 2 diabetes mellitus: a cross-sectional survey in Ethiopia.	Kassahun, T., et.al.(2016)	*	*	*			*	*	*	*	7	Good
73	Diabetes-related Knowledge, Medication Adherence, and Health-related Quality of Life: A Correlation Analysis.	Ishaq, R., et.al.(2021)	*	*		√		*		*	*	5	Moderate
74	Evaluation of factors affecting adherence to asthma controller therapy in chest clinics in a sub-Saharan African setting: a cross-sectional study.	Ngahane, B.H.M., et.al.(2016)	√	√	*			*		*	*	4	Unsatisfactory

75	Medication adherence and 24-h blood pressure in apparently uncontrolled hypertensive Nigerian patients.	Adeoye, A.M, et.al.(2019)		*		*			√	*		*	*	5	Moderate
76	Adherence to antihypertensive medications among adult hypertensive patients attending chronic follow-up units of Dessie Referral Hospital, Northeastern Ethiopia: A cross-sectional study.	Andualem, A., et.al.(2021)	*			*		*		*	*	*	*	7	Good
77	Sleep duration and treatment compliance: a population-based cross-sectional study of hypertensive patients in Bangladesh.	Hossain, A., et.al.(2016)			√		√	*		*		*	*	4	Unsatisfactory
78	Antihypertensive medication adherence and associated factors among adult hypertensive patients at Jimma University Specialized Hospital, southwest Ethiopia.	Asgedom, S.W, et.al.(2018)	*			*		*		*	*	*	*	7	Good

79	Medication Adherence and Diabetes Self-Care Activities Among Patients With Type 2 Diabetes Mellitus.	Jannoo, Z., et.al.(2019)		*		*		*		*	*	*	*	7	Good
80	Evaluation of medication adherence in Lebanese hypertensive patients.	Mohammad, Y., et.al.(2016)	*			*		*		*		*	*	6	Moderate
81	Adherence to treatment and social support in patients with non-insulin dependent diabetes mellitus.	Garay-Sevilla, M., et.al.(1995)			√		√		√	*		*	*	3	Unsatisfactory
82	Adherence to oral anti-diabetic drugs among patients attending a Ghanaian teaching hospital.	Bruce, S.P, et.al.(2015)	*			*		*		*		*	*	6	Moderate
83	Factors affecting medication adherence in patients with hypertension.	Karakurt, et.al.(2012)		*			√		√	*		*	*	4	Unsatisfactory
84	Locus of control and anti-hypertensive medication adherence in Ghana.	Kretchy, I.A., et.al.(2014)			√	*			√	*		*	*	4	Unsatisfactory

85	Drug adherence for antihypertensive medications and its determinants among adult hypertensive patients attending in chronic clinics of referral hospitals in Northwest Ethiopia.	Mekonnen, H.S., et.al.(2017)	*		*		*		**			*	*	7	Good
86	Factors impacting on diabetes knowledge, medication adherence and glycemic control among adult diabetics visiting a county teaching and referral hospital in Kenya: a cross-sectional study.	Mwangasha, et.al.(2021)		√	*			√		*		*	*	4	Unsatisfactory
87	Evaluation of illness perceptions and their associations with glycaemic control, medication adherence and chronic kidney disease in type 2 diabetes mellitus patients in Malaysia.	Balasubramaniam, S., et.al.(2019)	*			√	*			*	*	*	*	6	Moderate
88	Systemic Arterial Hypertension in the Emergency Service: medication adherence and	Vancini-Campanharo, et.al.(2015)		√		√		√		*		*	*	3	Unsatisfactory

	understanding of this disease.														
89	Identification of psychosocial factors of noncompliance in hypertensive patients.	Hassan, N.B, et.al.(2006)		√		√	*		**		*	*	*	6	Moderate
90	Disease related knowledge, medication adherence and glycaemic control among patients with type 2 diabetes mellitus in Pakistan.	Nazir, S.U.R, et.al.(2016)	*		*			√	**		*	*	*	7	Good
91	Adherence to Anti-Hypertensive Medications among Patients in Selected Health Facilities of Nepal.	Khadka, et.al.(2021)	*		*			√		*	*	*	*	6	Moderate
92	The Association between Diabetes-Related Distress and Medication Adherence in Adult Patients with Type 2 Diabetes Mellitus: A Cross-Sectional Study.	Kretchy, et.al.(2020)	*		*			√	**		*	*	*	7	Good

93	Factors associated with suboptimal adherence to hypertensive medications among syrian refugees ? Cross-sectional study at the Zaatari camp, Jordan	Khudair, et.al.(2021)	*			√		√	**		*	*	*	6	Moderate
94	Association Between Medication Literacy and Medication Adherence Among Patients With Hypertension.	Shi, et.al.(2019)	*		*		*		**		*	*	*	8	Good
95	Illness perceptions of Libyans with T2DM and their influence on medication adherence: a study in a diabetes center in Tripoli.	Ashur, S.T., et.al.(2015)		*	*		*		**		*	*	*	8	Good
96	Drug adherence behavior among hypertensive out-patients at a tertiary health institution in Manicaland province, Zimbabwe, 2011.	Mukora-Mutseyekwa, F.N., et.al.(2013)		*		√		√		*	*	*	*	5	Moderate
97	The association between self-perceptions of aging and antihypertensive medication	Hou, Y., et.al.(2016)		*		√	*		**		*	*	*	7	Good

	adherence in older Chinese adults.												
98	Antihypertensive Medication Adherence and Associated Factors: A Cross-Sectional Analysis of Patients Attending a National Referral Hospital in Asmara, Eritrea.	Mebrahtu, et.al.(2021)	*	*	*			*	*	*	*	7	Good
99	Medication adherence and its associated factors among Chinese community-dwelling older adults with hypertension.	Wang, W., et.al.(2014)	*	*		√	**		*	*	*	7	Good
100	Beliefs about medicines and non-adherence in patients with stroke, diabetes mellitus and rheumatoid arthritis: a cross-sectional study in China.	Wei.L, et.al.(2017)	*	*		√	**		*	*	*	7	Good
101	Self-reported adherence to therapeutic regimens among patients with hypertension.	Al-Daken, et.al.(2017)	*	*		√	**		*	*	*	7	Good

102	Adherence to insulin therapeutic regimens in patients with type 1 diabetes. A nationwide survey in Brazil.	Gomes, et.al.(2016)	*		*			√	**		*	*	*	7	Good
103	Association of Social Support and Medication Adherence in Chinese Patients with Type 2 Diabetes Mellitus.	Gu, L., et.al.(2017)	*			√		√	**		*	*	*	6	Moderate
104	Exploring variables associated with medication non-adherence in patients with COPD.	Jarab, et.al.(2019)	*			√	*			*	*	*	*	6	Moderate
105	Assessment of patient medication adherence among the type 2 diabetes mellitus population with peripheral diabetic neuropathy in South India	Samu, et.al.(2017)		*		√		√		*		*	*	4	Unsatisfactory
106	Modifiable Factors Associated with Non-adherence to Antihypertensive or Antihyperlipidemic Drugs Are Dissimilar: a Multicenter Study Among Patients with	Alfian, S.D, et.al.(2020)	*		*		*		**		*	*	*	8	Good

	Diabetes in Indonesia.													
107	Adherence and quality of life among diabetic patients with hypertension	Akrom, A., et.al.(2018)	*			√	√		*	*	*	*	5	Moderate
108	Diabetes knowledge, medication adherence and glycemic control among patients with type 2 diabetes	Al-kazaaz, H.K., et.al.(2011)	*		*		√		*	*	*	*	6	Moderate
109	Effect of medications-related beliefs on adherence to treatment of type II diabetes mellitus in a primary healthcare setting, Addis Ababa, Ethiopia	Bizu, G., et.al.(2016)	*		*		√	**		*	*	*	7	Good
110	Non-adherence and Associated Factors among Type 2 Diabetic Patients at Jimma University Specialized Hospital, Southwest Ethiopia	Teklay, G., et.al.(2013)		√		√	√		*	*	*	*	4	Unsatisfactory

111	Antihypertensive medication non-adherence and its determinants among patients on follow up in public hospitals in Northern Ethiopia	Ali, M. A, et.al.(2014)	√	√	√	*	*	*	*	4	Unsatisfactory
112	Medication adherence among hypertensive patients of primary health clinics in Malaysia	Ramli, A., et.al.(2012)	*	*	√	**	*	*	*	7	Good
113	Adherence to drug treatment in hypertensive patients on the Family Health Program	Ungari, A.Q., et.al.(2010)	*	*	√	*	*	*	*	6	Moderate
114	Does treatment adherence correlates with health related quality of life? findings from a cross sectional study	Saleem, F., et.al.(2012)	*	*	√	**	*	*	*	7	Good
115	Association Between Knowledge and Drug Adherence in Patients With Hypertension in Quetta, Pakistan	Saleem, F., et.al.(2011)	*	*	√	**	*	*	*	7	Good
116	Assessment of Factors Influencing Adherence to Antidiabetic Drugs among Patients with Type 2 Diabetes	Nandini, H.C, et.al.(2020)	√	*	√	*	*	*	*	5	Moderate

	Mellitus at a Tertiary Care Hospital in India													
117	Predictors of poor adherence to antidiabetic therapy in patients with type 2 diabetes: A cross-sectional study insight from Ethiopia	Demoz, G.T, et.al.(2020)	*		*		*		*	*	*	*	7	Good
118	Treatment adherence and factors contributing to non adherence among type 2 diabetes mellitus patients in a tertiary care hospital: a cross sectional study	Kavitha, S, et.al.(2017)		√		√		√	*	*	*	*	4	Unsatisfactory
119	Adherence and Associated Factors towards Antidiabetic Medication among Type II Diabetic Patients on Follow-Up at University of Gondar Hospital, Northwest Ethiopia	Abebaw, M., et.al.(2016)	*		*		*		*	*	*	*	7	Good
120	Medication adherence among ambulatory patients with type 2 diabetes in a tertiary	Adisa, R., et.al.(2010)	*		*		*		*	*	*	*	7	Good

	healthcare setting in Southwestern Nigeria														
121	Medication adherence and Its Correlates among Diabetic Patients in Central Hospital, warri, nigeria	Ogheonovo, A. , et.al.(2016)		√	*			√		*		*	*	4	Unsatisfactory
122	Factors Contributing To Non-Adherence To Medication Among Type 2 Diabetes Mellitus In Patients Attending Tertiary Care Hospital In South India	Divya, S., et.al.(2015)		√	*			√			*	*	*	4	Unsatisfactory
123	Nonadherence and Contributing Factors among Ambulatory Patients with Antidiabetic Medications in Adama Referral Hospital	Gelaw, B.K, et.al.(2014)	*		*		*		**		*	*	*	8	Good
124	Factors related to patient adherence to antidiabetic drug therapy	Gimenes, H.T, et.al.(2009)		√		√		√		*	*	*	*	4	Unsatisfactory

125	Patterns and obstacles to oral antidiabetic medications adherence among type 2 diabetics in Ismailia, Egypt: a cross section study	Heissam, K., et.al.(2015)	√	√	√	*	*		*	*	4	Unsatisfactory
126	Treatment adherence to oral glucose-lowering agents in people with diabetes: Using the Brief Medication Questionnaire	Istili, P.T., et.al.(2015)	√	√	√	*	*		*	*	4	Unsatisfactory
127	Adherence to Oral Antidiabetic Medications among Type 2 Diabetic (T2DM) Patients in Chronic Ambulatory Wards of Hiwot Fana Specialized University Hospital, Harar, Eastern Ethiopia: A Cross Sectional Study	Jemal, A., et.al.(2017)	√	√	√	*	*		*	*	4	Unsatisfactory
128	Diabetes knowledge and medication adherence among geriatric patient with type 2 diabetes mellitus	Omar, M.S., et.al.(2014)	√	√	√	*	*		*	*	4	Unsatisfactory

129	Blood glucose control and medication adherence among adult type 2 diabetic Nigerians attending a primary care clinic in under-resourced environment of eastern Nigeria	Iloh, G.U.P, et.al.(2012)		*		*				√		*	*	*	*	6	Moderate
130	Medication adherence to antidiabetic therapy in patients with type 2 diabetes mellitus	Sajith, M., et.al.(2014)			√		√			√		*	*	*	*	4	Unsatisfactory
131	The adherence to medications in diabetic patients in rural Kerala, India	Sankar, U.V, et.al.(2015)	*			*		*			**		*	*	*	8	Good
132	Measuring the rate of therapeutic adherence among outpatients with T2DM in Egypt	Shams, M.E.E., et.al.(2010)			√		√		√			*	*	*	*	4	Unsatisfactory
133	Assessment of Antidiabetic Medication Adherence and Its Determinants among Ambulatory Patients with Type 2 Diabetes	Tsehay, T., et.al.(2016)	*			*				√		*	*	*	*	6	Moderate

134	Assessment of Medication Adherence and its Association with Glycemic Control among Type-2 Diabetes Mellitus Patients in Gaza–Palestine	MR, Almadhoun, et.al.(2018)		*	*		*		**		*	*	*	8	Good
135	Factors associated with adherence to treatment of patients with diabetes mellitus	Faria, H.T.G, et.al.(2013)	*		*		*			*	*	*	*	7	Good
136	Assessment of patient's antihypertensive medication adherence level in non-comorbid hypertension in a tertiary hospital in Nigeria	Okoro, R.N., et.al.(2012)		*		√		√		*		*	*	4	Unsatisfactory
137	Medication belief as correlate of medication adherence among patients with diabetes in Edo State, Nigeria.	Olorunfemi, O., et.al.(2019)		*		√		√	**		*	*	*	6	Moderate

138	Relationship between the 6-item Morisky medication adherence scale (MMAS-6) score and glycemic control in a Turkish population with type 2 diabetes mellitus	Son, N.E., et.al.(2017)		√	√		√		*	*	*	*	4	Unsatisfactory
139	Association between patients' beliefs and oral antidiabetic medication adherence in a Chinese type 2 diabetic population	Wu, P., et.al.(2016)	*		√		√	**		*	*	*	6	Moderate
140	Role of illness and medication perceptions on adherence to medication in a group of Iranian patients with type 2 diabetes.	Aflakseir, A., et.al.(2011)	*		√	*		**			*	*	6	Moderate
141	Treatment adherence and risk of non-compliance among hypertensives at a Teaching Hospital in Ogun state, southwest Nigeria	Atulomah, N.O, et.al.(2010)		√	√		√		*	*	*	*	4	Unsatisfactory

142	Prevalence and Factors Associated with Non-Adherence to Antidiabetic Medication Among Patients at Mbarara Regional Referral Hospital, Mbarara, Uganda	Faisal, K., et.al.(2022)	*	*	*			*	*	*	*	7	Good
143	Prevalence, perceptions and practices associated with non-adherence to diabetes medications in primary care setting: A cross sectional study in urban Bangalore	Inbaraj, L.R, et.al.(2016)	*	*		√		*	*	*	*	6	Moderate
144	Association between the 8-item Morisky medication adherence scale (MMAS-8) score and glycaemic control among Chinese diabetes patients	Wong, M.C.S, et.al.(2015)	*	*	*		**		*	*	*	8	Good
145	Type II DM Medication Non-Adherence in Adama Hospital Medical College, Central Ethiopia	Kusa, W., et.al.(2019)	*	*	*			*	*	*	*	7	Good

146	Contributing Factors of Non-Adherence to Treatment among the Patients with Type II Diabetes Mellitus	Bhattarai, B., et.al.(2019)	*			√		√		*		*	*	4	Unsatisfactory
147	Factors Associated with Medication Nonadherence among Hypertensives in Ghana and Nigeria	Boima, V., et.al.(2015)			√	√		√		*		*	*	3	Unsatisfactory
148	Evaluation of adherence to therapy in patients of type 2 diabetes mellitus	Sontake, S., et.al.(2015)			√	√		√		*		*	*	3	Unsatisfactory
149	Patients' adherence-related beliefs about inhaled steroids: application of the Chinese version of the Beliefs about Medicines Questionnaire-specific in patients with asthma	Cai, Q., et.al.(2020)		*		√		√	**		*	*	*	6	Moderate
150	Factors Associated With Poor Medication Adherence In Hypertensive Patients In Lusaka, Zambia	Mwenee, M.D, et.al.(2010)		*		√	*		**			*	*	6	Moderate
151	Poor adherence to treatment: A major challenge in diabetes	Sharma, T., et.al.(2014)			√	√		√		*		*	*	3	Unsatisfactory

152	Adherence to pharmacotherapy and medication-related beliefs in patients with hypertension in Lima, Peru.	Arias, M.F., et.al.(2014)	*			√	*		**		*	*	6	Moderate
153	Perception of uncontrolled blood pressure and non-adherence to anti-hypertensive agents in diabetic hypertensive patients.	Ledur, P.S., et.al.(2013)	*			√	*		**		*	*	6	Moderate
154	Association between adherence, treatment satisfaction and illness perception in hypertensive patients	Saarti, S., et.al.(2016)		√		√	*			*	*	*	4	Unsatisfactory
155	The role of psychosocial determinants in predicting adherence to treatment in patient with hypertension	Asgari, M.R., et.al.(2019)		√		√		√	**		*	*	4	Unsatisfactory
156	Adherence to Antihypertensive Medications: Population Based Follow up in Eastern Nepal	Bhandari, B., et.al.(2015)	*		*			√		*	*	*	5	Moderate
157	Drug Non-Adherence In Type 2	Shams, N., et.al.(2016)		√		√		√		*	*	*	3	Unsatisfactory

	Diabetes Mellitus; Predictors And Associations											
158	Medication adherence and its associated factors among hypertensive patients attending the Debre Tabor General Hospital, Northwest Ethiopia	Teshome, D.F., et.al.(2017)	*	*	*		*	*	*	*	7	Good
159	Effect of medication adherence on blood pressure control and risk factors for antihypertensive medication adherence	Yue, Z., et.al.(2015)	*	*	*		*	*	*	*	7	Good
160	Factors affecting medication adherence in community-managed patients with hypertension based on the principal component analysis: Evidence from Xinjiang, China	Zhang, Y., et.al.(2018)	*	*	*	**		*	*	*	8	Good

<u>Selection: (Maximum 5 stars)</u>

1) Representativeness of the sample
    a) Truly representative of the average in the target population. * (all subjects or random sampling)

b) Somewhat representative of the average in the target population. * (non-random sampling)
c) Selected group of users.
d) No description of the sampling strategy.

2) Sample size
   a) Justified and satisfactory. *
   b) Not justified

3) Non-respondents
   a) Comparability between respondents and non-respondents characteristics is established, and the response rate is satisfactory. *
   b) The response rate is unsatisfactory, or the comparability between respondents and nonrespondents is unsatisfactory.
   c) No description of the response rate or the characteristics of the responders and the nonresponders.

4) Ascertainment of the exposure (disease)
   a) Validated measurement tool. **
   b) Non validated measurement tool, but the tool is available or described. *
   c) No description of measurement tool.

Comparability: (Maximum 2 stars)

1) The subjects in different outcome groups are comparable, based on the study design or analysis. Confounding factors are controlled.
   a) The study controls for the most important factors,. i.e. other factors potentially impairing cognitive outcome. *
   b) The study controls for any additional factor. *

Outcome: (Maximum 3 stars)

1) Assessment of the outcome:
   b) Independent structured assessment. **

c) Record linkage. **
d) Self report or educational level as reported by school *.
e) No description.

2) Statistical test:
   a) The statistical test used to analyze the data is clearly described and appropriate (e.g. comparison to healthy population, or normative scores), and the measurement of the association is presented, including confidence intervals and the probability level (p value). *
   b) The statistical test is not appropriate, not described or incomplete.

Scores for cross-sectional studies

Very good studies: 9-10 points

Good studies: 7-8 points

Moderate studies: 5-6 points

Unsatisfactory studies: 0 to 4 points

Supplementary 10. COSMIN Measurement Properties Checklist

Author (Year)	Instrument type	Internal Consistency						Cross Cultural Validity				
		1	2	3	4	5	CONCL	1	2	3	4	CONCL
Zyoud, S.H, et.al.(2013)	MMAS-8	VG	NA	VG	NA	VG	VG	VG	VG	VG	VG	VG
Sweileh, W. M, et.al.(2014)	MMAS-8	I	NA	I	NA	I	I	VG	VG	VG	VG	VG
Shakarneh, et.al.(2020)	MMAS-4	I	NA	I	NA	I	I	VG	D	VG	I	I
Al-Ramahi, R., et.al.(2015)	MMAS-8	I	NA	I	NA	I	I	VG	VG	VG	VG	VG
Khdour, M.R., et.al.(2020)	MMAS-4	D	NA	VG	NA	VG	D	VG	VG	VG	VG	VG
Elsous, A. , et.al.(2017)	MMAS-4	VG	NA	VG	NA	VG	VG	VG	VG	VG	VG	VG
Salama, H.M, et.al.(2020)	MMAS-8	VG	NA	VG	NA	VG	VG	VG	D	I	VG	I
Abebe, S.M. , et.al.(2014)	MMAS-8	I	NA	I	NA	I	I	VG	I	VG	I	I
Malik, A., et.al.(2014)	MMAS-4	I	NA	I	NA	I	I	VG	I	VG	I	I
Jamous, R.M , et.al.(2011)	MMAS-8	I	NA	I	NA	I	I	VG	VG	D	VG	D
Sarkodie, E., et.al.(2020)	MMAS-8	I	NA	I	NA	I	I	VG	I	VG	I	I
Nazir, S.U.R, et.al.(2017)	MMAS-8	VG	NA	VG	NA	VG	VG	VG	VG	VG	VG	VG

Turan, G.B, et.al.(2019)	MASES	VG	VG	NA	NA	VG	VG	VG	VG	VG	VG	VG
Nguyen, T., et.al.(2019)	MMAS-8	I	I	I	NA	I	I	VG	I	D	I	I
Cai, Q., et.al.(2021)	MARS-A	VG	VG	NA	NA	VG	VG	VG	D	VG	D	D
Erku, D.A., et.al.(2017)	MMAS-8	I	I	I	NA	I	I	VG	I	D	I	I
Teshome, D.F., et.al.(2018)	MMAS-4	D	NA	I	NA	I	I	VG	D	I	I	I
Pan, J., et.al.(2019)	TASHP	VG	VG	NA	NA	VG	VG	A	A	VG	VG	A
Ajayi, D.T, et.al.(2018)	MMAS-8	VG	VG	VG	NA	VG	VG	D	D	VG	D	D
Shakya, et.al.(2020)	Hill-Bone medication adherence scale	VG	VG	NA	NA	VG	VG	VG	VG	VG	VG	VG
Adomako, N.O, et.al.(2021)	MARS-10	I	I	NA	NA	I	I	VG	I	VG	I	I
Pan, J., et.al.(2021)	Hill-Bone medication adherence scale	VG	VG	NA	NA	VG	VG	VG	VG	VG	VG	VG
Aminde, L.N., et.al.(2019)	MCQ	I	I	NA	NA	I	I	A	I	A	I	I
Ahmad, N.S., et.al.(2013)	MCQ	VG	VG	NA	NA	VG	VG	A	A	A	VG	A
Akintunde, A.A., et.al.(2015)	MMAS-8	I	I	I	NA	I	I	A	I	D	I	I
Akoko, B.M., et.al.(2017)	MMAS-8	I	I	I	NA	I	I	A	I	D	I	I
Chan, C.W., et.al.(2015)	MMAS-8	VG	VG	VG	NA	VG	VG	VG	VG	VG	VG	VG

Lulebo, et.al.(2015)	MMAS-4	I	I	I	NA	I	I	A	D	D	VG	D
Abbas, et.al.(2015)	MMAS-8	D	I	I	NA	I	I	A	D	D	VG	D
Arulmozhi, S., et.al.(2014)	MMAS-8	D	I	I	NA	I	I	D	A	A	VG	D
Ambaw, et.al.(2012)	MMAS-4	D	I	I	NA	I	I	D	I	D	I	I
Waari, G., et.al.(2018)	MMAS-8	D	I	I	NA	I	I	D	I	D	I	I
Ozoh, O.B., et.al.(2021)	MMAS-8	D	I	I	NA	I	I	D	I	D	I	I
Tefera, Y.G, et.al.(2020)	MMAS-4	D	I	I	NA	I	I	VG	VG	VG	VG	VG
Yuvaraj, K., et.al.(2019)	MMAS-4	D	I	I	NA	I	I	D	VG	VG	VG	D
Pirasath, S., et.al.(2020)	MMAS-7	D	I	I	NA	I	I	VG	VG	VG	VG	VG
Abba, Z.I., et.al.(2018)	MMAS-8	D	I	I	NA	I	I	D	I	D	I	I
Dego, T.R., et.al.(2016)	MMAS-4	D	NA	I	NA	I	I	D	I	D	I	I
Shimels, T., et.al.(2021)	MMAS-8	VG	VG	NA	NA	VG	VG	VG	VG	VG	VG	VG
Balasubramanian, A., et.al.(2018)	MMAS-4	D	NA	I	NA	I	I	D	I	D	I	I
Mahmood, S., et.al.(2020)	MMAS-8	VG	VG	NA	NA	VG	VG	VG	VG	VG	VG	VG
Chaudhary, A.P., et.al.(2021)	Hill-Bone medication adherence scale	D	I	NA	NA	I	I	D	I	D	I	I
Mathur, D., et.al.(2020)	MMAS-8	D	I	NA	NA	I	I	D	I	D	I	I

Kassahun, A., et.al.(2016)	MMAS-4	D	I	NA	NA	I	I	D	I	D	I	I
Okeke, H.C., et.al.(2019)	MMAS-8	D	I	NA	NA	I	I	D	I	D	I	I
Afaya, R.A., et.al.(2020)	MAQ	VG	VG	NA	NA	VG	VG	VG	VG	VG	VG	VG
Iloh, G.U.P, et.al.(2017)	no name	D	I	NA	NA	I	I	D	I	D	I	I
Takahashi, E., et.al.(2021)	MMAS-4	I	I	NA	NA	I	I	I	D	D	I	I
Amer, M., et.al.(2019)	MMAS-8	VG	VG	NA	NA	VG	VG	A	A	VG	VG	A
Nonogaki, A., et.al.(2019)	MMAS-4	VG	NA	VG	NA	VG	VG	A	A	VG	VG	A
Raja, W., et.al.(2021)	Hill-Bone medication adherence scale	D	I	NA	NA	I	I	D	I	D	I	I
Ali, M., et.al.(2017)	MMAS-8	D	I	NA	NA	I	I	D	I	D	I	I
Ullah, et.al.(2019)	Hill-Bone medication adherence scale	D	I	NA	NA	I	I	D	I	D	I	I
G/Tsadik, D., et.al.(2020)	MMAS-8	D	VG	NA	NA	D	D	D	I	D	I	I
Roka, et.al.(2020)	MMAS-4	D	I	NA	NA	I	I	D	I	D	I	I
Bakar, Z.A, et.al.(2016)	MMAS-8	VG	VG	NA	NA	VG	VG	VG	VG	VG	VG	VG
Yang, S., et.al.(2016)	MMAS-4	VG	NA	VG	NA	VG	VG	VG	VG	VG	VG	VG
Berhe, D.F., et.al.(2017)	MMAS-8	VG	VG	VG	NA	VG	VG	VG	VG	VG	VG	VG

Sefah, I.A., et.al.(2020)	MMAS-8	D	I	NA	NA	I	I	D	I	D	I	I
Saraiva, E.M.S, et.al.(2020)	MMAS-4	VG	VG	VG	NA	VG	VG	VG	VG	VG	VG	VG
Bermeo-Cabrera, J., et.al.(2018)	MMAS-8	D	I	NA	NA	I	I	D	I	D	I	I
Lerman, I., et.al.(2009)	Self Care Inventory Diabetes	D	I	NA	NA	I	I	D	I	D	I	I
Abate, T.W. , et.al.(2019)	MMAS-8	D	I	NA	NA	I	I	VG	VG	VG	VG	VG
Oluwole, E.O, et.al.(2019)	MMAS-8	D	VG	NA	NA	D	D	D	I	D	I	I
Mariye, T., et.al.(2019)	MMAS-8	D	I	NA	NA	I	I	VG	VG	VG	VG	VG
Jackson, I., et.al.(2015)	MMAS-8	D	I	NA	NA	I	I	D	I	D	I	I
Basharat, et.al.(2018)	MMAS-8	D	I	NA	NA	I	I	D	I	D	I	I
Fadare, J., et.al.(2015)	MMAS-8	D	I	NA	NA	I	I	D	I	D	I	I
Al-Qerem, W., et.al.(2021)	MMAS-4 and BMQ	VG	NA	VG	NA	VG	VG	VG	VG	VG	VG	VG
Iqbal, et.al.(2017)	DAI	VG	VG	VG	NA	VG	VG	VG	VG	VG	VG	VG
Akram, F., et.al.(2021)	MMAS-8	VG	VG	VG	NA	VG	VG	VG	VG	VG	VG	VG
Ayodapo, A.O., et.al.(2020)	MMAS-4	D	NA	I	NA	I	I	D	I	D	I	I
Hashmi, et.al.(2007)	MMAS-4	D	NA	I	NA	I	I	D	I	D	I	I
Asilar, H., et.al.(2020)	MASES-SF	VG	VG	VG	NA	VG	VG	VG	VG	VG	VG	VG

Kassahun, T., et.al.(2016)	MMAS-8	D	I	NA	NA	I	I	A	A	VG	VG	A
Ishaq, R., et.al.(2021)	DAI	D	D	NA	NA	D	D	D	D	D	D	D
Ngahane, B.H.M., et.al.(2016)	MMAS-8	D	I	NA	NA	I	I	D	I	D	I	I
Adeoye, A.M, et.al.(2019)	MMAS-4	VG	VG	VG	NA	VG	VG	D	I	D	I	I
Andualem, A., et.al.(2021)	MMAS-4	VG	NA	VG	NA	VG	VG	VG	VG	VG	VG	VG
Hossain, A., et.al.(2016)	SC	D	I	NA	NA	I	I	D	I	D	I	I
Asgedom, S.W, et.al.(2018)	MMAS-8	D	I	NA	NA	I	I	VG	VG	VG	VG	VG
Jannoo, Z., et.al.(2019)	MMAS-8	D	I	NA	NA	I	I	D	I	D	I	I
Mohammad, Y., et.al.(2016)	MMAS-8	D	I	NA	NA	I	I	A	A	VG	VG	A
Garay-Sevilla, M., et.al.(1995)	no name	D	I	NA	NA	I	I	D	I	D	I	I
Bruce, S.P, et.al.(2015)	MMAS-4	D	NA	I	NA	I	I	A	A	VG	VG	A
Karakurt, et.al.(2012)	no name	D	I	NA	NA	I	I	D	I	D	I	I
Alhaddad, et.al.(2016)	MMAS-4	D	NA	I	NA	I	I	D	I	D	I	I
Kretchy, I.A., et.al.(2014)	MMAS-8	D	I	NA	NA	I	I	D	I	D	I	I
Mekonnen, H.S., et.al.(2017)	MMAS-8	D	I	NA	NA	I	I	A	A	VG	VG	A

Mwangasha, et.al.(2021)	MMAS-8	D	I	NA	NA	I	I	D	I	D	I	I
Balasubramaniam, S., et.al.(2019)	MMAS-8	VG	VG	NA	NA	VG	VG	VG	VG	VG	VG	VG
Vancini-Campanharo, et.al.(2015)	MMAS-4 and BMQ	D	I	NA	NA	I	I	D	I	D	I	I
Hassan, N.B, et.al.(2006)	no name	VG	VG	NA	NA	VG	VG	VG	VG	VG	VG	VG
Nazir, S.U.R, et.al.(2016)	MMAS-8	VG	VG	NA	NA	VG	VG	VG	VG	VG	VG	VG
Khadka, et.al.(2021)	MMAS-4	D	I	NA	NA	I	I	D	I	D	I	I
Kretchy, et.al.(2020)	MARS-5	VG	VG	NA	NA	VG	VG	A	A	VG	VG	A
Sartori, et.al.(2020)	MMAS-4	D	VG	NA	NA	D	D	A	A	VG	VG	A
Owolabi, et.al.(2020)	no name	VG	VG	NA	NA	VG	VG	A	A	VG	VG	A
Alfian, S.D, et.al.(2021)	MARS-5	VG	VG	NA	NA	VG	VG	VG	VG	VG	VG	VG
Cani, et.al.(2015)	MMAS-4 and MAQ	VG	VG	NA	NA	VG	VG	VG	VG	VG	VG	VG
Khudair, et.al.(2021)	ARMS	VG	VG	NA	NA	VG	VG	I	A	VG	D	I
Chung, W.W, et.al.(2014)	MALMAS	VG	VG	NA	NA	VG	VG	VG	VG	VG	VG	VG
Shen, Y., et.al.(2019)	no name	VG	VG	NA	NA	VG	VG	VG	VG	VG	VG	VG
Delavar, F., et.al.(2020)	MMAS-8	VG	VG	NA	NA	VG	VG	VG	VG	VG	VG	VG
Barikani, A., et.al.(2021)	MARS-10	VG	VG	NA	NA	VG	VG	A	A	VG	VG	A
Sheilini, et.al.(2019)	MMAS-8	D	D	NA	NA	D	D	A	A	VG	VG	A

Nazir, S.U.R, et.al.(2020)	MMAS-8	VG	VG	NA	NA	VG	VG	VG	VG	VG	VG	VG
Nesari, M., et.al.(2010)	no name	VG	VG	NA	NA	VG	VG	A	A	VG	VG	A
Bijam, et.al.(2020)	no name	D	I	NA	NA	I	I	D	I	D	I	I
Chow, E.P., et.al.(2015)	MMAS-8	VG	VG	NA	NA	VG	VG	VG	VG	VG	VG	VG
Supachaipachnipong, S., et.al.(2018)	MMAS-8	VG	VG	NA	NA	VG	VG	VG	VG	VG	VG	VG
Shi, et.al.(2019)	MMAS-8	VG	VG	NA	NA	VG	VG	VG	VG	VG	VG	VG
Ashur, S.T., et.al.(2015)	MMAS-8	VG	VG	NA	NA	VG	VG	VG	VG	VG	VG	VG
Mukora-Mutseyekwa, F.N., et.al.(2013)	MMAS-4	D	NA	I	NA	I	I	D	I	D	I	I
Hou, Y., et.al.(2016)	MMAS-8	VG	VG	NA	NA	VG	VG	VG	VG	VG	VG	VG
Mebrahtu, et.al.(2021)	MARS-10	D	I	NA	NA	I	I	D	I	D	I	I
Wang, W., et.al.(2014)	MMAS-4	VG	NA	VG	NA	VG	VG	VG	VG	VG	VG	VG
Mohamad, et.al.(2021)	MMAS-8	D	I	NA	NA	I	I	VG	VG	VG	VG	VG
Wei.L, et.al.(2017)	MARS-5	D	VG	NA	NA	D	D	VG	VG	VG	VG	VG
Al-Daken, et.al.(2017)	Hill-Bone medication adherence scale	VG	VG	NA	NA	VG	VG	VG	VG	VG	VG	VG
Gomes, et.al.(2016)	MMAS-4	VG	NA	VG	NA	VG	VG	VG	VG	VG	VG	VG
Gu, L., et.al.(2017)	MMAS-8	VG	VG	NA	NA	VG	VG	VG	VG	VG	VG	VG
Jarab, et.al.(2019)	MMAS-4	D	I	NA	NA	I	I	D	I	D	I	I
Samu, et.al.(2017)	MMAS-8	D	I	NA	NA	I	I	D	I	D	I	I

Saleem, et.al.(2015)	DAI	VG	VG	NA	NA	VG	VG	VG	VG	VG	VG	VG
Abdulsalim, et.al.(2018)	MAQ	D	I	NA	NA	I	I	D	I	D	I	I
Alfian, S.D, et.al.(2020)	MARS-5	VG	VG	NA	NA	VG	VG	VG	VG	VG	VG	VG
Akrom, A., et.al.(2018)	MMAS-8	D	I	NA	NA	I	I	D	I	D	I	I
Al-kazaaz, H.K., et.al.(2011)	MMAS-8	VG	VG	NA	NA	VG	VG	VG	VG	VG	VG	VG
Bizu, G., et.al.(2016)	MMAS-8	VG	VG	NA	NA	VG	VG	VG	VG	VG	VG	VG
Teklay, G., et.al.(2013)	MMAS-4	D	I	NA	NA	I	I	D	I	D	I	I
Ali, M. A, et.al.(2014)	MMAS-8	D	I	NA	NA	I	I	D	I	D	I	I
Ramli, A., et.al.(2012)	MCQ	VG	VG	NA	NA	VG	VG	VG	VG	VG	VG	VG
Ungari, A.Q., et.al.(2010)	MMAS-4	D	I	NA	NA	I	I	D	I	D	I	I
Saleem, F., et.al.(2012)	DAI	VG	VG	NA	NA	VG	VG	VG	VG	VG	VG	VG
Saleem, F., et.al.(2011)	DAI	VG	VG	NA	NA	VG	VG	VG	VG	VG	VG	VG
Nandini, H.C, et.al.(2020)	MARS-5	D	I	NA	NA	I	I	D	I	D	I	I
Demoz, G.T, et.al.(2020)	BMQ	D	I	NA	NA	I	I	D	I	D	I	I
Kavitha, S, et.al.(2017)	MMAS-8	D	I	NA	NA	I	I	D	I	D	I	I
Abebaw, M., et.al.(2016)	MMAS-4	D	NA	I	NA	I	I	A	A	VG	VG	A

Adisa, R., et.al.(2010)	MMAS-4	D	NA	I	NA	I	I	D	I	D	I	I
Ogheonovo, A. , et.al.(2016)	MMAS-8	D	I	NA	NA	I	I	D	I	D	I	I
Divya, S., et.al.(2015)	MMAS-8	D	I	NA	NA	I	I	D	I	D	I	I
Gelaw, B.K, et.al.(2014)	no name	D	I	NA	NA	I	I	D	I	D	I	I
Gimenes, H.T, et.al.(2009)	MTA	D	D	NA	NA	D	D	A	A	VG	VG	A
Heissam, K., et.al.(2015)	MTA	D	I	NA	NA	I	I	D	I	D	I	I
Istili, P.T., et.al.(2015)	BMQ	D	I	NA	NA	I	I	D	I	D	I	I
Jemal, A., et.al.(2017)	MMAS-4	D	I	NA	NA	I	I	D	D	D	D	D
Omar, M.S., et.al.(2014)	MMAS-4	D	NA	I	NA	I	I	D	I	D	I	I
Iloh, G.U.P, et.al.(2012)	no name	D	I	NA	NA	I	I	D	I	D	I	I
Sajith, M., et.al.(2014)	MMAS-4	D	NA	I	NA	I	I	D	I	D	I	I
Sankar, U.V, et.al.(2015)	MMAS-8	D	D	NA	NA	D	D	VG	VG	VG	VG	VG
Shams, M.E.E., et.al.(2010)	MTA	D	I	NA	NA	I	I	D	I	D	I	I
Tsehay, T., et.al.(2016)	MMAS-4	D	NA	I	NA	I	I	D	I	D	I	I
MR, Almadhoun, et.al.(2018)	MMAS-8	VG	VG	NA	NA	VG	VG	VG	VG	VG	VG	VG

Faria, H.T.G, et.al.(2013)	MTA	D	NA	I	NA	I	I	D	I	D	I	I
Okoro, R.N., et.al.(2012)	no name	D	NA	I	NA	I	I	D	I	D	I	I
Olorunfemi, O., et.al.(2019)	MMAS-4	VG	NA	VG	NA	VG	VG	A	A	VG	VG	A
Son, N.E., et.al.(2017)	MMAS-6	D	D	NA	NA	D	D	D	D	D	D	D
Wu, P., et.al.(2016)	MMAS-8	VG	VG	NA	NA	VG	VG	VG	VG	VG	VG	VG
Aflakseir, A., et.al.(2011)	MARS-10	VG	VG	NA	NA	VG	VG	D	D	D	D	D
Atulomah, N.O, et.al.(2010)	no name	VG	VG	NA	NA	VG	VG	VG	VG	VG	VG	VG
Faisal, K., et.al.(2022)	Hill-Bone medication adherence scale	D	NA	I	NA	I	I	D	I	D	I	I
Inbaraj, L.R, et.al.(2016)	MMAS-4	D	NA	I	NA	I	I	D	I	D	I	I
Wong, M.C.S, et.al.(2015)	MMAS-8	VG	VG	NA	NA	VG	VG	VG	VG	VG	VG	VG
Kusa, W., et.al.(2019)	MMAS-8	D	I	NA	NA	I	I	D	I	D	I	I
Bhattarai, B., et.al.(2019)	MMAS-4	D	NA	I	NA	I	I	D	I	D	I	I
Boima, V., et.al.(2015)	MMAS-8	D	I	NA	NA	I	I	D	I	D	I	I
Sontake, S., et.al.(2015)	MMAS-8	D	I	NA	NA	I	I	D	I	D	I	I
Cai, Q., et.al.(2020)	MMAS-8	VG	VG	NA	NA	VG	VG	VG	VG	VG	VG	VG

Mwenee, M.D, et.al.(2010)	Hill-Bone medication adherence scale	VG	VG	NA	NA	VG	VG	A	A	A	A	A
Sharma, T., et.al.(2014)	MMAS-4	D	NA	I	NA	I	I	D	I	D	I	I
Arias, M.F., et.al.(2014)	MMAS-8	D	D	NA	NA	D	D	A	A	A	A	A
Ledur, P.S., et.al.(2013)	MMAS-4	VG	NA	VG	NA	VG	VG	VG	VG	VG	VG	VG
Saarti, S., et.al.(2016)	MMAS-8	D	NA	I	NA	I	I	D	I	D	I	I
Asgari, M.R., et.al.(2019)	MMAS-8	VG	VG	NA	NA	VG	VG	VG	VG	VG	VG	VG
Bhandari, B., et.al.(2015)	MMAS-4	VG	NA	VG	NA	VG	VG	VG	VG	VG	VG	VG
Shams, N., et.al.(2016)	MMAS-8	VG	VG	NA	NA	VG	VG	VG	VG	VG	VG	VG
Teshome, D.F., et.al.(2017)	MMAS-4	D	NA	I	NA	I	I	VG	VG	VG	VG	VG
Yue, Z., et.al.(2015)	MMAS-8	VG	VG	NA	NA	VG	VG	VG	VG	VG	VG	VG
Zhang, Y., et.al.(2018)	no name	VG	VG	NA	NA	VG	VG	VG	VG	VG	VG	VG

VG= Very Good; A= Adequate; D= Doubtful; I= Inadequate; NA= Not Applicable

Internal Consistency

1. Was an internal consistency statistic calculated for each unidimensional scare or subscale separately?
2. For continuous score: was Cronbach's alpha or omega calculated?
3. For dichotomous scores: was Cronbach's alpha or KR-20 calculated?

4. For IRT-based score: was standard error of the theta or reliability coefficient of estimated latent trait value (index of subject or item separation) calculated?
5. Were there any other important flaws in the design or statistical method of the study?

Cross Cultural Validity

1. Were the sample similar for relevant characteristic except for the group variable?
2. Was an appropriate approach used to analyse the data?
3. Was the sample size included in the analysis adequate?
4. Were there any other important flaws in the design or statistical method of the study?
